# Dietary Phenolics against Breast Cancer. A Critical Evidence-Based Review and Future Perspectives

**DOI:** 10.3390/ijms21165718

**Published:** 2020-08-10

**Authors:** María Ángeles Ávila-Gálvez, Juan Antonio Giménez-Bastida, Juan Carlos Espín, Antonio González-Sarrías

**Affiliations:** Laboratory of Food and Health, Research Group on Quality, Safety and Bioactivity of Plant Foods, Dept. Food Science and Technology, CEBAS-CSIC, P.O. Box 164, Campus de Espinardo, 30100 Murcia, Spain; mavila@cebas.csic.es (M.Á.Á.-G.); jgbastida@cebas.csic.es (J.A.G.-B.)

**Keywords:** breast cancer, polyphenols, in vitro, animal models, clinical trials, dietary phenolics

## Abstract

Breast cancer (BC) is the most common malignancy and the leading cause of cancer-related death in adult women worldwide. Over 85% of BC cases are non-hereditary, caused by modifiable extrinsic factors related to lifestyle, including dietary habits, which play a crucial role in cancer prevention. Although many epidemiological and observational studies have inversely correlated the fruit and vegetable consumption with the BC incidence, the involvement of their phenolic content in this correlation remains contradictory. During decades, wrong approaches that did not consider the bioavailability, metabolism, and breast tissue distribution of dietary phenolics persist behind the large currently existing gap between preclinical and clinical research. In the present review, we provide comprehensive preclinical and clinical evidence according to physiologically relevant in vitro and in vivo studies. Some dietary phenolics such as resveratrol (RSV), quercetin, isoflavones, epigallocatechin gallate (EGCG), lignans, and curcumin are gaining attention for their chemopreventive properties in preclinical research. However, the clinical evidence of dietary phenolics as BC chemopreventive compounds is still inconclusive. Therefore, the only way to validate promising preclinical results is to conduct clinical trials in BC patients. In this regard, future perspectives on dietary phenolics and BC research are also critically discussed.

## 1. Breast Cancer: General Aspects

Cancer is one of the world’s most substantial health problems and the second leading cause of death globally, with 9.6 million cancer-related deaths in 2018 [1,2]. Among women, breast cancer (BC) is the first cause of cancer-associated death and the most commonly diagnosed malignant tumour worldwide, accounting for 2.1 million new BC diagnoses in 2018 [3]. Although the advances in diagnosis, treatment and intensive research have significantly increased the survival rates among BC subjects in the past years, its incidence has increased worldwide, especially in developing countries. According to U.S. Breast Cancer Statistics, it is estimated that BC impacts nearly 1 in 8 women in their lifetime [4]. Only 10%–15% of all BC cases are hereditary, meaning that the vast majority are caused by modifiable risk factors, playing a central role in cancer prevention through lifestyle improvement, including dietary habits [5,6]. Apart from the dietary and lifestyle habits, other related factors to BC include age, ethnicity, hormonal status, hormonal therapy, ultraviolet radiation, and circadian disruption [7]. Regarding genetic risk factors, some mutations such as breast cancer susceptibility gene 1 (*BCRA1*), *BCRA2*, and *TP53* are generally accepted, although these mutations are rare and only constitute approximately 5%–10% of all BC rates incidences [5,8,9].

BC is a compilation of distinct malignancies that usually begins in the terminal duct lobular unit of the mammary gland and progresses in a stepwise manner by multiple molecular alterations. BC is highly heterogeneous, encompassing from some cases with excellent prognosis to very aggressive tumours, which usually develop over a long time [10,11]. Clinically, BC is classified into different grades and types based on histological characteristics. The histological grade is a well-established prognostic tool based on the degree of aggressiveness or differentiation of the tumour tissue, including a combined score for several parameters such as the microscopic evaluation of tubule or gland formation, nuclear pleomorphism, and the mitotic count (i.e., determination of the proliferation marker Ki-67 by immunohistochemistry). Therefore, BCs with a high histological grade are generally large and associated with metastasis, rather than those with low histological grade [12,13,14]. On the other hand, the histological type is based on the growth pattern of the tumours including different subtypes, presenting a higher incidence (around 75%), the so-called invasive or infiltrating ductal carcinomas of no particular type, compared with other different subtypes such as invasive lobular carcinoma, mucinous, tubular, medullary, etc. Thus, BCs with smaller tubular carcinomas are generally associated with an earlier stage of the tumour, compared with invasive ductal carcinomas [13,14]. Besides, the TNM is a well-accepted classification to designate the BC stage at the time of diagnosis refers to the size and invasiveness of the tumour (T), lymph node involvement (N), and presence of distant metastasis (M) [15]. 

Regarding molecular basis, BC is broadly categorized into different subtypes based on the combination (presence or absence) of three receptors for oestrogens (ER), progesterone (PR) and human epidermal growth factor receptor 2 (HER2). The differential expression of these receptors strongly determines the prognosis and therapeutic strategies against BC [11,14]. In this regard, the incidence of hormone receptor-positive (ER+ PR+) is the most common BC subtype, representing around 70%. It exhibits a better prognosis with high survival and low recurrence rates, compared with hormone receptor-negative tumours. Moreover, according to the elevated expression of HER2 and related genes, BC is further categorized into different molecular subtypes, such as luminal A (ER+ PR+ HER2-) and(or) luminal B (ER+ PR+ HER2+). HER2+ tumours represent a 15%–20% of BC cases. Interestingly, although this type is more aggressive, associated with an increase in Ki-67 protein, it responds better to the current therapy resulting in high survival rates [11,16,17]. Finally, the triple-negative BC for these three biomarkers (TNBC) accounts for about 15% of all BCs. It is characterized by the worst prognosis involving high invasive properties, high relapse rate, and high resistance to different therapies, what make this subtype highly intractable and invasive (mainly with the presence of metastasis in other organs) [11,18]. Six molecular subtypes of TNBC have been reported according to their gene ontologies and differential gene expression in specific genes involved in cell cycle, DNA damage response genes, androgen receptors, epidermal growth factor receptor, and signalling pathways, among others. This highlights the significance of molecular heterogeneity in BC [19].

Currently, beyond the prevention and early diagnosis, the therapeutic options for BC include surgical resection, radiation, as well as chemotherapeutic supplementation according to the molecular subtypes and the BC stage. Thus, for early-stage non-metastatic BC, surgical resection, together with postoperative adjuvant chemotherapy, radiotherapy, and(or) hormone therapy depending on the BC subtype, represent the main treatment options. On the other hand, treatments for metastatic BC (about 20%–45%) generally consist of conventional chemotherapy, including cytotoxic drugs, such as the anthracycline (doxorubicin), and(or) taxane (paclitaxel) families, combined with cyclophosphamide. Other treatments include 5-fluorouracil, cisplatin derivatives, gemcitabine, or vinorelbine, as well as monoclonal antibody therapy, such as bevacizumab that targets the vascular endothelial growth factor receptor (VEGFR), what in turn inhibits angiogenesis processes [11,20,21]. Fortunately, the knowledge of predictive biomarkers in the different BC subtypes has led to the use of combined treatments with adjuvant chemotherapy, allowing for a personalized approach in each patient that increases the effectiveness of the therapy. In this regard, the main treatments for hormone-dependent BC (ER+/PR+) combined with adjuvant chemotherapy are based on hormonal therapy, which is based on the use of oestrogen antagonists, such as tamoxifen or fulvestrant and aromatase inhibitors like anastrozole, letrozole, and exemestane [22]. In HER2 overexpressing tumours, the primary treatment involves monoclonal antibodies such as trastuzumab and pertuzumab and specific HER2 pathway inhibitors like lapatinib. This treatment could be combined with hormonal therapy, depending on the hormone receptors’ expression [20,21,23]. As mentioned above, the lack of biomarkers expression in TNBC is addressed through classical chemotherapy (anthracyclines, taxanes, platinum-containing chemotherapeutic agents, etc.) as the unique therapeutic option [11,20,21]. However, adverse side effects of these anticancer drugs alone or in combination are relatively frequent. Furthermore, the further development of drug resistance processes leads to tumour recurrences after long-time administration.

Therefore, beyond the currently approved chemotherapeutics, natural products (including dietary compounds) exhibit pleiotropic, multi-target activities and are emerging as possible complementary chemopreventive molecules against BC with fewer side effects than conventional therapy [6,24].

## 2. Epidemiological Evidence for Dietary (Poly)Phenols

As stated above, it is well-established that lifestyle improvement, including dietary habits, is the primary prevention strategy against cancer. Indeed, improper dietary habits (high amounts of fat, sugar, red meat, and alcohol) may be behind 30%–35% of all the cancer types [25,26]. In contrast, epidemiological and observational studies have described the inverse association between high intake of plant foods and low BC risk [27,28], or high vegetable intake and low ER-BC risk [29,30]. Nevertheless, several studies indicate that this inverse correlation for high consumption of fruit and vegetables remains still inconsistent or contradictory [6,29,31], and limited evidence has been found according to the recent European Prospective Investigation into Cancer and Nutrition (EPIC) data [31,32].

Over the past decades, meta-analyses, observational, and epidemiological studies have suggested the potential role of dietary phenolics in the chemoprevention and chemosensitization in BC [33,34,35]. In this regard, significant protective effects against BC have been attributed to some dietary patterns that involve high intake of phenolics-rich foods, mainly phytoestrogens that can bind to ER, such as isoflavones in soy-based products in Asian countries [36,37,38]. Mediterranean dietary patterns implicate higher intake of polyphenols than Western diets, especially in postmenopausal women and against TNBC [39,40].

Many dietary phenolics have been reported to exert anticancer activities by playing pleiotropic multi-target activities on BC cells and animal models related to cell viability, proliferation, differentiation, and invasion, as well as by modulating signalling pathways, BC markers, gene, and protein expression, etc. [41,42]. However, human evidence remains still inconclusive and controversial.

## 3. Bioavailability of Dietary Phenolics and Their Occurrence in Human Breast Tissues

The bioavailability of dietary phenolics is an essential factor to consider, either increasing or limiting the overall anticancer effects against systemic cancers such as BC. In line with this, understanding the metabolic/molecular forms and concentrations of those phenolics that can reach the human breast tissue, is crucial in order to suggest their possible role in the in vivo beneficial effects, as well as to (re)design physiologically relevant in vitro studies, thus increasing the knowledge of their possible chemopreventive mechanisms of action.

Without considering the food matrix and(or) food processing related factors, the rate and extent of intestinal absorption of each phenolic compound depend on the chemical structure that determines its solubility, membrane permeability, and its microbial and(or) phase-II metabolism [43,44,45].

Dietary phenolics are usually characterized by their poor bioavailability, limiting their distribution in systemic tissues in their native form, mainly as glycosides and complex oligomeric structures. Once ingested, they are transformed into a variety of diverse bioavailable metabolites that can remain in the systemic circulation for a few days [46]. Most phenolics are hydrolyzed and further metabolized by either the intestinal enzymes or by the gut microbiota. After absorption, the resulting polyphenol-derived metabolites undergo extensive phase-II metabolism inside the enterocytes to form sulphates, glucuronides, methyl-derivatives, or other conjugates. Therefore, in contrast to their parental molecules that rarely appear in the systemic circulation, significant concentrations of phenolic-derived conjugated metabolites can be detected in plasma and target systemic tissues where might trigger biological effects, including anticancer activity [46,47,48].

However, it was found that conjugated forms of phenolics or microbial metabolites (phase-II derivatives) display much lower or even no anticancer effects than their corresponding precursor unconjugated forms [49,50,51]. Therefore, to date, whether dietary phenolics may protect against the progression of BC (despite the low bioavailability and high metabolism of these compounds) is unclear, and several fundamental questions about their mechanism of action remain unanswered.

Regarding the occurrence of phenolic-derived metabolites in human breast tissue, the evidence is limited to a low number of human intervention studies, in which the sample size is also small (from 3 to 31 volunteers) and the characteristic of the volunteers are different between the studies (Table 1). In contrast, the number of animal studies is higher, but the results reported are contradictory. The methodologies used for quantifying the metabolites in most of these studies showed low accuracy and should be considered with caution (Table S1).

Similar to animal studies, the most robust human evidence is reflected in investigations with isoflavones. Four clinical studies, conducted in women undergoing aesthetic breast, have allowed us to establish the identity and quantity of the isoflavone metabolites that can occur in human breast tissues after the intake of soy-based foods. Overall, these studies showed that unconjugated daidzein, genistein, and the daidzein microbial-derived metabolite, equol, reached concentrations in the low nanomolar range, reporting higher levels for equol (in those equol-producer individuals) and daidzein than genistein [52,53]. However, no significant differences between daidzein and genistein were observed in another study in women undergoing breast biopsy or cancer surgery [54]. Notwithstanding, it should be noted that the breast tissues were hydrolyzed by enzymatic treatment in these studies. Thus, the levels of these phenolics were overestimated, i.e., the unconjugated phenolics did not reach the tissue as such, but the enzymatic treatment in the tissue generated them.

In this regard, Bolca et al. identified and quantified the distribution of isoflavones in non-hydrolyzed and enzymatically hydrolyzed adipose and glandular breast tissues after the intake soy milk or supplement by healthy women undergoing an aesthetic breast reduction [55]. As expected, the results showed overall total glucuronidation of 98% (900–1150 pmol/g total isoflavone glucuronides), mainly genistein-7-*O*-glucuronide and daidzein-7-*O*-glucuronide, whereas only traces of free genistein and daidzein were observed (20–25 pmol/g), but not for the microbial daidzein-derived metabolites (equol or *O*-DMA). However, after enzymatic hydrolysis, equol and dihydrodaidzein were also detected in a few subjects, in addition to high levels of unconjugated daidzein and genistein.

According to this fact, in those animal studies where non-hydrolyzed samples were also analyzed compared with hydrolyzed ones, isoflavones-derived metabolites were mostly detected (>60%) in their physiologically conjugated forms, mostly glucuronides, and the unconjugated forms were even undetectable in some cases (Table S1) [56,57,58]. Besides, it should be considered that only an animal study reported the distribution in breast tumour tissues of isoflavones metabolites, and described higher levels in tumour than in normal (liver and colon) tissues, for both total and free genistein, and daidzein to a lesser extent [59]. Bolca et al., following the same approach and methodology, also observed extensive glucuronidation (>90%) for prenylflavonoids in breast adipose/glandular tissues [60]. However, low concentrations (picomolar, ranging from 0.8 to 83.7) of total isoxanthohumol (IX), xanthohumol (XN) and 8-prenylnaringenin (8-PN, only in those 8-PN producers) were quantified in hydrolyzed tissues from healthy women undergoing an aesthetic breast reduction after hop intake for 5 days [60]. This result was in agreement with those reported in rodent models where 8-PN and 6-PN glucuronides were predominantly found in the mammary gland tissue after intraperitoneal administration of prenylflavonoids or hop extract (Table S1) [61,62].

In recent years, new human studies have further advanced in the identification of metabolites in human breast tissue, through the combination of the analysis in hydrolyzed and non-hydrolyzed samples and the evaluation of the distribution in both normal and malignant tissues. This fact is especially relevant since breast tumour and normal tissues have been reported to show differences in phase-II enzymes expression such as lower and higher activities of UDP-glucuronosyltransferases and glutathione S-transferases, respectively [63], as well as increased the β-glucuronidase activity when exposed to inflammatory or hypoxic conditions within the tumour microenvironment [63,64].

Thus, in two pre-surgery non-controlled dietary interventions with silybin and tea extracts conducted in patients with newly diagnosed BC, higher concentrations of total silybin and EGCG were detected in tumour tissues than in the adjacent normal tissues. In both studies, similar to other phenolics, the total concentration of conjugated forms was higher compared with free silybin and EGCG (4- and 2-fold, respectively), suggesting a high rate of phase-II metabolism, although free EGCG was not detected in tumour rather than normal tissues. However, the quantification of conjugates was not performed due to the lack of conjugated standards [65,66]. Similar results (considering the limitations of the conjugated forms quantification) related to the identification of methyl and sulphate of catechins have been observed in two animal studies conducted in mice fed green tea extracts. However, it is noteworthy that besides the higher quantification of EGCG, other catechins such as epicatechin and epigallocatechin were also detected in hydrolyzed samples (Table S1) [67,68].

Finally, a recent pre-surgery-controlled intervention study with a higher number of BC patients that consumed a cocktail of plant extracts (cocoa, pomegranate, lemon, orange, grapeseed, olive, and RSV) identified a total of 39 and 33 metabolites in normal and tumour tissues, respectively. However, although the qualitative and quantitative metabolic profiling of phenolics was quite similar in normal and tumour tissues, higher levels were found for most phenolics in tumour tissues.In both tissues, phenolic-derived metabolites, including RSV, urolithins, and hesperetin, showed levels in the range of the low nM, and were mainly glucuronidated and(or) sulphated (>85%). Among these conjugates, the percentage of sulphated was slightly lower in normal tissues (31%) than in tumour (42%) [69].

More recently, the same authors tried to overcome one of the main limitations of these human studies, i.e., the long pre-surgery fasting that could hamper: (i) the detection of other phenolic-derived metabolites (including different metabolic forms), (ii) the occurrence of higher concentrations that could be reached in the breast tissues without fasting period. In this study, conducted in rats fed equivalent doses to those used in the human study, the analyses at shorter times described the presence of conjugated, but not free metabolites, derived from RSV, hesperetin, urolithins. These results are in agreement with the data described in the previous human study, although some differences were observed: higher concentrations (range of low µM), a higher proportion of RSV sulphates than glucuronides (opposite to what is observed in humans), as well as the occurrence of other metabolites at shorter times such as hydroxytyrosol glucuronide and sulphate [70]. These results corroborate some limitations of human studies (associated with hospital protocols) and confirm that human and animal studies carried out under similar conditions contribute to increasing our knowledge on the bioavailability of dietary phenolics.

Regarding other phenolics with high potential anticancer activity like curcumin (diferuloylmethane) or lignans, the evidence is even more limited, with insufficient studies. Only studies with rodent models have detected their presence in breast tissues (Table S1). Like other phenolics, the accumulation of free and conjugated gut microbiota-derived enterolignan metabolites, mainly enterolactone and enterodiol glucuronides, has been identified in the mammary tissues of rats and pigs (Table S1) [71,72].

Overall, based on the current evidence, the distribution of phenolic-derived metabolites in breast tissue is similar to that observed in plasma, yet at lower concentrations. However, in contrast to most phenolics, curcumin, beyond its poor bioavailability, remains a challenge. Thus, despite curcumin is rapidly metabolized to conjugated forms, mainly glucuronides, recent animal studies have identified free curcumin at significantly higher concentrations (up to 15-fold) than curcumin glucuronide in breast tumour tissues from tumour-bearing mice models, after oral and intravenous administration [73]. Besides, similar to that observed for other phenolics, curcumin levels in healthy mammary tissues were significantly lower than tumours tissues [73]. This is in agreement with another recent animal study where the concentration of free curcumin in serum was lower than its glucuronide. However, the absolute amount and the percentage of total curcumin was significantly increased in bones (up to 3 µM), whereas curcumin glucuronide was barely detectable due to the high glucuronidase activity [74]. At present, this fact has not been confirmed in human studies hitherto.

On the other hand, it is also important to note that recent preclinical studies have explored (non-nutritional) strategies to increase polyphenols’ bioavailability and stability to overcome the fast clearance of most phenolic-derived metabolites [46]. Different techniques are included within these alternative approaches, such as encapsulation (micelles, liposomes, nanoparticles, etc.) and phenolic loaded self-microemulsifying drug delivery system (SMEDDS) or self-nanoemulsifying drug delivery system (SNEDDS), which are used for different phenolics (curcumin, quercetin, RSV, etc.) in both bioavailability and chemopreventive animal studies (Tables S1 and S2). Similar approaches to increase cytotoxicity and bioavailability have also been assayed in BC cell models [75,76,77].

## 4. Evidence of the Chemopreventive Potential of Phenolic Compounds in Physiologically Relevant Preclinical Studies

Following the statement “First in vivo and then in vitro” [78], we must be cautious with all the accumulated evidence on in vitro polyphenols’ effects against BC. In this regard, innumerable in vitro studies have been carried out with doubtful physiological relevance, i.e., regardless of bioavailability, metabolism, and tissue distribution of dietary phenolics or their derived metabolites. Therefore, in the present review, we focus only on those physiologically relevant in vitro and in vivo studies conducted with dietary phenolics that could elucidate whether they are responsible for the effects attributed to plant-based foods.

In the last decades, in vivo animal studies have been an essential tool in investigating the biological effects of natural compounds against BC. More than 200 in vivo studies, included in this review (Table S2), describe the effects of different phenolic compounds on BC. Isoflavones (genistein and daidzein), as well as lignans (flaxseed and secoisolariciresinol diglucoside), encompass the highest number of studies (almost 50%), whereas the effects of other classes, including anthocyanins or flavones have been less investigated so far. In general, diets supplemented with phenolics (anthocyanins, flavanones, flavones, flavonols, flavan-3-ols, RSV, curcumin, lignans, and hydrolysable tannins) were associated with reduction of tumour growth, incidence (percentage of animals that develop breast tumours), multiplicity (tumours per mouse), metastasis (mainly to lungs), and higher tumour latency period (time between carcinogen exposure and tumour detection), which in turn are related to a higher survival rate. However, these effects are less clear regarding the consumption of isoflavones.

A significant number of investigations challenges the reported evidence concerning the benefits of soy products and isoflavones consumption in the prevention or treatment of BC. At least 15 studies (out of 52 included in Table S2) described the absence of protection or even the increase of tumour growth, incidence, metastasis, or reduction of tumour latency. These contradictory results have led the food scientists to search for new approaches (i.e., a combination of different phenolic compounds) to reduce the stimulatory effects of isoflavones on human breast tissue. In this regard, the combination of flaxseed, enterolactone, and(or) enterodiol with soy protein or genistein has demonstrated to be effective attenuating tumour growth promotion in animal models [79,80].

On the other hand, the combination of phenolics is not limited to lignans-rich foods and isoflavones. Different mixtures such as quercetin-RSV-genistein, curcumin-EGCG, and curcumin-RSV have been shown to be effective in reducing tumour growth and modulating apoptosis and angiogenesis signalling pathways [81,82,83]. In line with this, emerging strategies using self-nanoemulsifying drug delivery systems (SNEDDS) showed higher plasma concentrations of mixtures of phenolics related to more efficient anti-breast cancer effects [84].

Regarding the mechanism of action, the reduction on tumour growth associated with the consumption of the different dietary phenolics has been reported to be closely related to higher levels of apoptosis and lower cell proliferation and angiogenesis (together with the modulation of molecular mechanisms associated). Additional mechanisms described in the numerous preclinical studies included in Table S2 support the potential protective role of the phenolics against BC.

One of these mechanisms is the modulation of morphological changes in mammary tissue structures. Isoflavones [85,86] and resveratrol [87] have shown the capacity to reduce the number of terminal end buds (TEBs). The reduction of the TEBs density is considered a protective mechanism since these structures are targets of carcinogens like DMBA. Thus, the reduction of tumour growth in DMBA-treated rats fed grape juice concentrate was related to a decrease in the formation of DMBA-DNA adducts [88]. RSV also exhibited protection through the reduction of lipid peroxidation and DNA damage related to its antioxidant activity [89]. Further mechanisms by which phenolics exert their effects on breast tumours involve the regulation of oestrogen/progesterone levels and receptors, including ER, PR, and HER2. The reduction of 17β-oestradiol and progesterone levels was described in breast cancer animal models fed diets containing hesperetin and soy protein isolated [90,91] as well as luteolin and genistein in the presence of letrozole and tamoxifen, respectively [92,93]. Likewise, pomegranate emulsion, flaxseed, and secoisolariciresinol modulated the expression of ER, PR, and HER2 [94,95,96].

Moreover, the benefits of the phenolic compounds against BC are also linked to their anti-inflammatory effects. Dual inhibition of 5-LOX and COX-2 [97] and the modulation of cytokines regarding BC [98] are interesting strategies in the treatment or prevention of this disease. Inhibition of 5-LOX (and its product LTB4) and COX-2 via NF-κB inhibition by resveratrol consumption has been reported to be involved in BC prevention in DMBA-treated rats [89,99]. Naringenin and soy protein modulated the expression of genes involved in regulating the inflammatory response and cytokine signalling, including MCP-1 and IL-6 [90,100], while wogonoside and the mix resveratrol-quercentin-genistein reduced the level of IL-6, TNF-α, and the TNF receptor-associated factors TRAF2 and TRAF4 [84,101]. Several studies have highlighted the role of the immune system regarding the protective effects of phenolic compounds against BC. Thus, quercetin and silybin failed to protect against tumour development in immunodeficient mice. However, they were able to regulate the accumulation of immune cells (i.e., T-cells) as well as the biosynthesis of cytokines (i.e., TNF-α, IL-1β, IL-2, IL-4, IL-10, and INF-γ) in immunocompetent BALB/c mice [102,103]. Further evidence is supported by experimental animal models fed EGCG and naringenin-enriched diet showing accumulation and activation of T-cells in response to tumour-promoting stimuli [104,105].

Regarding in vitro studies, Table 2 highlights the limited current evidence on the anticancer effects for relevant phenolic-derived metabolites against different BC cell models. All in vitro studies corroborated that phase-II conjugation reduced the cytotoxic and(or) antiproliferative effect against different BC cell models and oestrogenic/anti-oestrogenic effects in ER+ MCF-7 cells. The most robust evidence has been reported in few studies for phase-II metabolites of RSV, quercetin, and isoflavones indicating potential effects but lower than their counterpart unconjugated forms. Besides, a few in vitro studies made a comparison between BC and non-tumourigenic cells reporting the lack of cytotoxic or antiproliferative effects in normal cells, unlike BC cell lines, at the tested concentrations and times (Table 2) [106,107,108,109]. On the other hand, to date, no effect has been reported for phase-II metabolites to other phenolic groups such as flavanones, urolithins and catechins, although the number of in vitro studies conducted is still scarce. However, these results should be interpreted with caution since the concentrations of conjugates in most in vitro studies are supra-physiological (>10 µM) (Table 2).

Although the conjugated metabolites are less bioactive than their free forms, and it remains difficult to attribute their role in the chemopreventive in vivo effects, new approaches are being investigated to confirm their possible in vitro anticancer activity. For instance, recent results have been obtained assaying concentrations close to those detected in vivo (up to 10 µM) of RSV-derived phase-II conjugates [109]. This study attributed the anticancer activity of RSV conjugates to cellular senescence induction in BC cells and, therefore, deserves further investigation.

To the best of our knowledge, there are no in vitro studies conducted with phase-II curcumin and enterolignans metabolites despite the large number of studies conducted with their unconjugated compounds or enriched extracts. The lack of availability of these relevant metabolites may be behind this gap.

On the other hand, it should not be discarded the potential role of phase-II metabolites (mainly glucuronides and sulfates) as a source of bioactive aglycones in breast tumour tissues. This process might occur via deconjugation under specific stimuli such as inflammation within the tumour microenvironment, where the ratio glucuronyltransferase/glucuronidase is lower than in normal tissues [64]. However, results that support this hypothesis are still limited to a few in vitro and animal studies [74,110].

## 5. Dietary (Poly)Phenols and Clinical Studies in Breast Cancer Patients

Table 3 summarizes the current evidence regarding the effects of several dietary (poly)phenols or phenolic-containing products in breast cancer patients. The main search criteria used were as follows: (i) clinical trials dealing with BC patients (either with active disease or cured patients to prevent recurrence), (ii) evaluation of the effects of orally administered (poly)phenols or phenolic-containing products (pure standards, plant extracts or foodstuffs) on tumour biomarkers or other clinically-relevant outcomes related to BC, including the decrease of side-effects associated with chemo- or radiotherapy (i.e., intravenous administration or articles dealing with only ‘hot flashes’, ‘wellness’ or other general determinations were excluded). Articles dealing with either healthy or at-risk volunteers (including postmenopausal women) were omitted, as were those studies conducted with non-dietary sources. Trials, where metabolites disposition was evaluated in malignant breast tissue from patients, were also included only when some effects on tumour-related biomarkers were evaluated.

As expected, the evidence on the anticancer effects of dietary phenolics in BC patients is limited, fragmented, and inconclusive. In this context, the most studied polyphenols are EGCG, as a component of green tea extracts, and curcuminoids present in curcumin extracts.

The pioneer pilot study of Thompson et al. revealed the increase of apoptosis, and the reduction of both Ki-67 (a cell proliferation marker) and c-erB2 (also known as HER-2) expression in 19 BC patients that consumed 25 g/day flaxseed vs. 13 patients that consumed placebo for approximately 37 days from the diagnosis until the resection of the tumour [125]. Despite the promising results, it took almost 10 years for a new study on flaxseed to be published by McCann et al. [126]. This new study was also a pre-surgery pilot study in which postmenopausal women diagnosed with ER+ BC consumed flaxseed (25 g/day) alone or combined with the aromatase inhibitor anastrozole or placebo, between tumour biopsy and resection (mean of 19 days). The authors found a reduction of endogenous oestrogen production (androstenedione in the flaxseed + anastrozole group, and dehydroepiandrosterone in the flaxseed group) (Table 3) [126]. Aromatase inhibitors block the enzyme aromatase that catalyzes the conversion of androgens into oestrogens, the primary source of endogenous oestrogens in postmenopausal women. This is relevant since anti-estrogen therapy aims to prevent access of the tumour to oestrogen and has been considered the standard of care in ER+ tumours [127].

Green tea extracts are the most widely tested polyphenol-containing products to date in BC patients (Table 3). The main active component is supposed to be EGCG, but the specific association between EGCG and the reported outcomes has not been unequivocally proven so far. Green tea extracts have been assayed to explore the effects on cancer biomarkers after short treatments (pre-surgery) [66,128] as well as after longer treatments (6 months) to assess the maximum tolerability dose (MTD) [129] and effects on some cancer-related biomarkers [129,130]. The MTD was established at 1200 mg/day for 6 months in patients with ER- and PR- breast tumours, but no significant changes were observed on ER, Ki-67, IGF-1, IGFBP-3, and mammographic density vs. placebo after 6 months [129]. The same authors explored the effects on more biomarkers in the same group of patients, but no effects were observed on VEGF, oxidative damage, and inflammatory markers, among others, but only a transient decrease of serum HGF in green tea consumers vs. placebo [130]. The other pre-surgery studies reported a significant decrease of Ki-67 (only in healthy breast tissue, but not in the malignant one), while no effects were observed on apoptosis (caspase-3) and angiogenesis (CD34) in both healthy and malignant breast tissues after consuming green tea extract (2.2 g/day for 35 days) [128]. More recently, Lazzeroni et al. evaluated the disposition of EGCG in normal and malignant tissues from 12 patients with ER+ tumours, but the authors did not find any effect on cell proliferation, angiogenesis, oxidative stress, inflammation or other markers [66]. However, they found a significant increase (36%) in testosterone levels. Although testosterone can be aromatized to oestradiol, which increases proliferation and hence, BC risk, testosterone has also been associated with BC prevention [131,132].

Curcumin extracts, also containing the related curcuminoids, demethoxycurcumin and bisdemethoxycurcumin, have also been assayed in BC patients. The first trial was conducted by Bayet-Robert et al. in 14 patients with metastatic breast cancer to evaluate the MTD or oral curcumin in combination with intravenous administration of docetaxel (Table 3) [133], a chemotherapeutic drug used in metastatic BC. The authors found a high MTD of oral curcumin (8 g/day) in combination with the drug (100 mg/m^2^), administered every 3 weeks for six cycles, with a total of 63 cycles, and observed a significant decrease of plasmatic CEA and VEGF from the 3rd cycle. However, no clear association was found between markers decrease and the occurrence of curcumin in plasma [133]. Later, Ryan et al. observed the protection of oral curcumin (6 g/day) administration vs. placebo after 6 months against radiation dermatitis in BC patients (n = 30) with prescribed radiation therapy (RT) [134]. The same authors tried to confirm their previous exploratory results in an ambitious trial that enrolled 686 patients with prescribed RT [135]. Nevertheless, curcumin did not reduce radiation dermatitis severity at the end of RT compared to placebo after 6 months (30 RT sessions). Overall, this is a warning to consider the minimum number of patients necessary to conclude the possible effects associated with the consumption of phenolics.

Other trials with BC patients and phenolics or phenolic-containing products, included the assay of grapeseed [136], milk thistle [65], and red clover [137] extracts, walnuts [138], and also a product containing a mixture of EPA, DHA hydroxytyrosol and curcumin [139]. In one of the pioneer studies dealing with polyphenols and BC, Brooker et al. did not find any effect on tissue hardness due to RT in 69 BC patients after consuming a grapeseed proanthocyanidins extract (300 mg/day) for 12 months vs. placebo [136]. Lazzeroni et al. described the disposition of some metabolites in breast tissue. However, they did not observe any effect on NO, IGF-1, and Ki-67 in 12 patients with ER+ breast cancer after consuming 2.8 g/day of a silybin-containing milk thistle extract in combination with phosphatidylcholine for 4 weeks between biopsy diagnosis and resection [65]. The only study dealing with food and BC has been conducted so far by Hardman et al. [138]. These authors conducted a pre-surgery trial in 10 BC patients that consumed around 30 g walnuts/day for 15 days (between tumour biopsy and resection). Surprisingly, with only 10 patients, and taking into account the huge inter-individual variability in gene expression and the previously reported artefacts [140,141], they observed the activation of genes participating in pathways that promote apoptosis and cell adhesion and inhibition of pathways that promote cell proliferation and migration (involving 456 genes in the tumour) due to walnut consumption [138]. Recently, Martínez et al. described the anti-inflammatory effect (decrease of plasma CRP vs. placebo) in BC patients with no active disease, and receiving adjuvant hormonal therapy, after consuming a mixture containing 460 mg fish oil (EPA, DHA), olive extract (125 mg hydroxytyrosol) and 50 mg curcumin extract for 30 days [139]. Although the authors described a relevant decreased of plasma CRP from 8.2 mg/L to 5.3 mg/L, unfortunately, the final CRP levels were in the boundary of those associated with patient’s survival [142] and still associated with high cardiovascular risk [143]. Besides, the responsible compound for the anti-inflammatory effect observed was not identified from such a complex mix. Finally, Ferraris et al. recently evaluated the effect of a red clover extract containing isoflavones (80 mg/day) to reduce side-effects of tamoxifen treatment in patients with ER+ BC under tamoxifen treatment for 2 years. Although the consumption of red clover extract was safe, no effects were observed in the targets related to tamoxifen treatment (menopausal rating score, sex hormone levels, endometrial thickness, and breast density, among others). Only BMI and waist circumference were significantly reduced in the red clover group vs. placebo [137].

## 6. Identifying the Existing Gaps to Address Future Preclinical and Clinical Research

As thoroughly detailed in the present review, despite substantial progress made during the last decades in advancing our knowledge of how dietary phenolics exert their biologic effects and, consequently, how they may be involved in the prevention and treatment of BC, to date, minimal success has been achieved.

Overall, several reasons have led to the vast gap between in vitro and in vivo evidence. Among the main reasons, it should be highlighted the low number of well-conducted human studies reported so far. Furthermore, it is essential to point on several central issues found in clinical trials, and pending aspects that should be addressed in future clinical studies:

(i) There is a need for multidisciplinary teams beyond researchers (oncologists, surgeons, pathologists, etc.) to conduct dietary interventions in BC patients. Indeed, this issue is inherent in every clinical trial dealing with cancer patients. In the case of ‘dietary interventions’, oncologists usually oppose the assay of high concentrations of dietary phenolics to patients undergoing chemotherapy due to the limited knowledge regarding the possible interactions between dietary compounds, including phenolics, and chemotherapy drugs. Overall, this challenging logistic yields a low number of recruited BC patients. Consequently, a restricted number of samples to explore or validate results, which is even more challenging when assaying molecules with relatively low anticancer activity compared to standard chemotherapy drugs.
(ii) The high heterogeneity of the results obtained. Inter-individual variability is perhaps one of the most critical aspects to establish definite conclusions in clinical studies. The low number of volunteers, as well as the specific selection criteria of the volunteers selected in the different studies, are key factors to understand the high variability reported. Genetic polymorphisms or differences in the composition or functionality of the gut microbiota will also contribute to the different response of individuals to interventions with polyphenol-rich foods [48,144,145,146]. In this regard, a highly variable metabolism between individuals has been described for some phenolic compounds. This allows for grouping the population according to the excretion of the metabolized compound into “high or low producers”, as it occurs in the metabolism of the phenolic precursor-derived metabolite pairs hesperidin-hesperetin [147], lignans-enterolignans [148], ellagic acid-urolithins [149] and procyanidins-valerolactones [150,151]. Besides, specific metabotypes, attributed to the different composition and functionality of the gut microbiota, have been described in the case of urolithins [146,152] or isoflavones [153], allowing the stratification of individuals based on the production of specific metabolites associated with a particular gut microbiota ecology. Thus, the inter-individual variability related to bioavailability and metabolism of dietary phenolics is essential to comprehend the results in clinical studies and to identify whether the possible beneficial effects can be extrapolated to the whole population or only to certain specific individuals [146]. Future research should be conducted to evaluate associations between specific phenolic-related gut microbiota metabotypes and protective effects against BC.
(iii) The difficulty of attributing chemopreventive effects to phenolics. Most human studies have been conducted with phenolic-containing products or extracts, which makes it challenging to identify whether single phenolics are (or not) actually responsible for the possible anti-tumour effect. Along this line, it is important to consider the differences in phenolic composition between plant foods, the difficulty in the estimation of the dietary intake of phenolics modulated by several factors such as food matrix, food processing-related factors, as well as the interaction between phenolics and the gut microbiota, and also other food components, such as proteins and carbohydrates that will undoubtedly influence the bioavailability and subsequent potential chemopreventive effects [45,154].

Another element that can help establish causality between effect and polyphenols is the determination of the derived metabolites in the target breast tissue. However, the characterization of the metabolic profiling of phenolic-derived metabolites in breast tissues remains poorly explored. Several reasons contribute to this gap: the lack of pure standards (mostly phase-II conjugates) that prevent their accurate quantification, and the accessibility to enough mammary biopsies to perform the analyses. Besides, the lack of standardized analytical protocols contributes to reporting incomplete or even misleading identifications/quantifications (heterogeneity of samples, hydrolysis vs. non-hydrolysis approaches, etc.).

Finally, it should be taken into account that phenolics generally exert relatively low biological activity, which makes difficult to discern their possible anticancer effects. In this regard, clinical studies should be focused on looking for effects upon long-term consumption of phenolic-rich foods. Therefore, in the coming years, there is a need to improve and re-design clinical trials to demonstrate the efficacy of dietary phenolics against BC unequivocally.

(iv) There is not enough evidence regarding the possible interaction between dietary phenolics and conventional chemotherapy drugs. Despite the preclinical evidence that supports the potential benefits of dietary phenolics as possible adjuvants in BC management [155,156], to date, only three clinical studies have been conducted using a combination of phenolics and chemotherapeutic drugs [126,137,139]. Therefore, there is a need to evaluate the potential interactions between phenolics and chemotherapeutic drugs to support phenolics’ usefulness as adjuvants in BC treatment and further follow-up of patients to prevent relapses.

As far as chemoprevention is concerned, the assayed conditions in animal models that cannot be extrapolated to humans, and the non-physiological in vitro approaches were predominant in the past. Nowadays, the trend is to perform physiologically relevant studies, as highlighted in recent guidelines [78,157]. Regarding BC, attention should be paid to the following gaps and pitfalls performed in the past strategies for phenolics’ chemoprevention determination in animal and cellular BC models:

(i) The difficulty of extrapolating the results obtained from animal research to humans. As in the case of human studies, several problems can be found in the BC animal models to evaluate the chemopreventive effect of phenolics: the methodology issues (low number of animals, and lack of controls, etc.), the heterogeneity of data, the difficulty of attributing chemopreventive effects to phenolics (use of whole-foods or extracts instead of single phenolics, determination of phenolics in breast tissues, etc.).

Besides, other intrinsic aspects should be considered and improved in animal research: any breast tumour-induction process (chemically-induced BC, murine mutant models, etc.) can reflect the heterogeneity of human BCs. It is habitual to use inadequate doses and exposure times for single phenolics, food, or extracts. Instead, doses should be equivalent to those achievable in humans after the regular intake of the phenolic-containing foods. The systemic administration of phenolics or food extracts (intravenous, intraperitoneal, etc.) is not representative of a dietary context and omits the significant handicaps of bioavailability and metabolism. Finally, the suitability of the specific animal model, according to the capability of each species to metabolize phenolic compounds, is of particular interest, especially in polyphenol-related gut microbiota metabotypes (urolithin and isoflavone metabotypes).

Besides, we must add to all the above logical inter-species differences (rats, mice, pigs, etc.), and even within strains from the same animal model, which contribute to the great challenge of extrapolating in vivo results to BC patients [158]. Nevertheless, animal research remains an essential step before clinical trials, mainly in evaluating the interaction with chemotherapeutics.

(ii) In vitro studies must have physiological relevance to establishing potential effects and conclusions. Different aspects of in vitro research should be avoided: the use of both unrealistic concentration and metabolic forms of phenolics; the unsuitability of BC cell models using a single cell line instead of considering the heterogeneity of cell lines using multiple cell lines with different mutations and other genetic characteristics (ER/PR positive or negative, etc.), or even more realistic advanced models such as primary cell cultures, organoids, etc.; and testing single phenolic or derived metabolite without considering the real mixture of compounds present in vivo, as well as the food matrix effect, avoiding the possible synergistic, antagonistic, or additive effects among them. Therefore, following these principles, transferring the information on the pharmacokinetic properties and bioavailability of phytochemicals in breast tissues plays one of the critical roles for a better evaluation of their mechanism of action involved in their BC chemoprevention.

## 7. Concluding Remarks

According to the detailed literature survey, including only physiologically relevant preclinical studies and clinical studies, we can conclude that some dietary phenolics, especially RSV, quercetin, isoflavones, EGCG, lignans, and curcumin, seem to be the most promising candidates. However, the clinical evidence is still minimal when compared with that obtained in preclinical studies.

As stated above, the increase and development of highly robust, well-designed clinical studies in BC patients and the quantitative and qualitative determination of phenolic-derived metabolites that reach breast tissues will make possible the continuous progress of the preclinical research. This approach will allow a more in-depth understanding of the molecular mechanisms involved in their chemopreventive and(or) chemotherapeutic properties and, perhaps, allowing the establishment of preventive nutritional strategies.

In the “omics” era, complementary approaches, including in silico and molecular docking studies, as well as high-throughput “omics” technologies, such as (epi)genomics, proteomics, and metabolomics are rapidly gaining interest to identify new ligands for specific cancer molecular targets in order to improve the therapeutic potential of both phenolics and their metabolic derivatives [159,160]. Besides, the analysis of the composition and functionality of the intra-tumour microbiota in breast cancer has been reported to be useful to predict different responses between tumour types and subtypes against immunotherapy [161]. Whether the microbiota of breast cancer, particularly rich and diverse, could modulate dietary phenolics’ effects deserves further research.

## Figures and Tables

**Table 1:** Dietary phenolics and derived metabolites identified in human breast tissues.

Group and Sample Size	Diet/Compound Administration	Extraction and Analytical Conditions	Identified and(or) Quantified Phenolic Metabolites	References
8 women undergoing breast biopsy or cancer surgery	Intake of four soy-supplemented bread rolls per day, providing approximately 45 mg of isoflavonoids, for 14 days prior to surgery (n = 4) Placebo group (n = 4).	Breast cancer tissues were extracted and further hydrolyzed by enzymatic treatment ^1^. Quantitative analyses were performed by GC/MS using specific standards.	The mean daidzein concentration in non-soy-supplemented patients was 0.021 (range 0.017–0.028) nmol/g compared with 0.145 (range 0.083–0.218) nmol/g in soy-supplemented patients.	[54]
3 healthy women undergoing breast reductions	Soy-based food supplement containing 100 mg of genistein/genistin, 37 mg of daidzein/daidzin, and 15 mg of glycitein/glycitin (more than 90% as glycosides (single and triple dose, n = 1 each) or a placebo tablet (n = 1) for 5 days before aesthetic breast surgery.	Breast tissues were extracted and further hydrolyzed by enzymatic treatment ^1^. Analyses were performed by HPLC-DAD and the compounds were identified by comparison of the retention time with the respective standards (UV spectra).	Genistein and equol concentrations were 4.16 µg/g and 52.98 µg/g, respectively, after soy-based food supplement (single dose), whereas daidzein was below the limit of detection. Genistein, equol and daidzein concentrations were 35.1 µg/g, 681.7 µg/g and 16.0 µg/g, respectively, after soy-based food supplement (triple dose).	[52]
28 volunteers before aesthetic breast surgery	Soy-based food supplement containing 100 mg of genistein/genistin, 37 mg of daidzein/daidzin, and 15 mg of glycitein/glycitin (more than 90% as glycosides, for 5 evenings before aesthetic breast surgery (n = 9). Placebo group (n = 19).	Breast tissues were extracted and further hydrolyzed by enzymatic treatment ^1^. Analyses were performed by HPLC-MS and the compounds were quantified using specific standards.	The median daidzein and equol concentrations were 7.03 nmol/L and 2.44 nmol/L, respectively, in soy-supplemented subjects. Genistein was not detectable. The median daidzein concentration was 5.44 nmol/L in the placebo group. Equol and genistein were only detectable in some subjects of the placebo group, with equol ten-fold values higher than genistein. No significant differences were found between the 2 groups.	[53]
31 healthy women undergoing an aesthetic breast reduction	Soy milk (n = 11; 250 mL containing 16.98 mg genistein and 5.40 mg daidzein (per dose)), soy supplement (n = 10; 5.27 mg genistein and 17.56 mg daidzein aglycone (per dose), or control (n = 10, no soy product). 3 doses of soy milk or soy supplements were taken daily for 5 days before an aesthetic breast reduction.	Breast tissues were dissected into fractions (adipose and glandular tissue) and were both non-hydrolyzed and hydrolyzed by enzymatic treatment ^1^. Quantitative analyses were performed by HPLC-MS/MS and the compounds were quantified using specific standards of aglycones, but not conjugated forms.	Total isoflavones showed a breast adipose/glandular tissue distribution of 40:60. In hydrolyzed breast adipose and glandular tissues, total genistein and daidzein concentrations ranged between 92.33–493.8 pmol/g and 22.15–770.8 pmol/g. Total equol and dihydrodaidzein were only detected in few subjects (up to 559.4 pmol/g and up to 368.8 ± 171.1 pmol/g. In non-hydrolyzed breast adipose and glandular tissues only traces of genistein and daidzein aglycones were observed (20–25 pmol/g total isoflavone aglycones), whereas an overall total glucuronidation of 98% was estimated (900–1150 pmol/g total isoflavone glucuronides), mainly genistein-7-*O*-glucuronide and daidzein-7-*O*-glucuronide. Neither glucuronides nor aglycones of microbial daidzein metabolites (equol and *O*-DMA) were detected after isoflavone supplementation.	[55]
21 healthy women undergoing an aesthetic breast reduction	Hop-supplemented group (containing 2.04 mg xanthohumol (XN), 1.20 mg isoxanthohumol (IX), and 0.1 mg 8-prenylnaringenin (8-PN) per supplement) (n = 11) or control group (n = 10). Three supplements were taken daily for 5 days before surgery.	Breast tissues were dissected into fractions (adipose and glandular tissue) and were both non-hydrolyzed and hydrolyzed by enzymatic treatment ^1^. Qualitative analyses were performed by HPLC-MS/MS and the compounds were quantified using specific standards for aglycones, but not conjugated forms.	Total prenylflavonoids showed a breast adipose/glandular tissue distribution of 38:62. Total XN and IX concentrations ranged between 0.26 and 5.14 pmol/g and 1.16 and 83.67 pmol/g in hydrolyzed breast tissue, respectively. 8-PN was only detected in samples of moderate and strong 8-PN producers (0.78–4.83 pmol/g). An extensive glucuronidation was observed (>90%).	[60]
12 early breast cancer patients	Orally bioavailable complex of silybin–phosphatidylcholine (2.8 g/day) for 4 weeks prior to surgery.	Breast tissues (tumour and normal) were extracted and further hydrolyzed both with and without enzymatic treatment ^1^. Qualitative analyses were performed by HPLC-MS/MS and the free and total silybin were quantified using specific standard.	The median total and free silybin concentration in breast cancer tissues were 131 ng/mg (IQR, 35–869) and 33 ng/g (IQR, 4–158), respectively. Concentrations were higher in the tumour as compared with the adjacent normal tissue (total and free silybin concentration were 11 ng/mL (IQR, 0–34) and 0 ng/g (IQR, 0–19), respectively).	[65]
12 early breast cancer patients	Patients received 300 mg of a caffeine-free green tea catechin extract, equivalent to 44.9 mg of epigallocatechin-3-O-gallate (EGCG) daily, for 4 weeks prior to surgery.	Breast tissues (tumour and normal) were extracted and further both non-hydrolyzed and hydrolyzed by enzymatic treatment ^1^. Quantitative analyses were performed by HPLC-MS/MS and the compounds were quantified as free (unconjugated) EGCG, thereafter through enzymatic hydrolysis as total EGCG (free and conjugated) using EGCG standard.	Total EGCG was detectable in all tumour tissue samples (median total EGCG 3.18 ng/g (IQR, 2.76–4.58)) and higher amount than in adjacent normal tissue (under the limit of detectability, minimum, 0 ng/g; maximum, 2.85 ng/g). Free EGCG concentrations were under the limit of detectability in tumour tissue but present in adjacent normal breast tissue (median = 1.07 ng/g; IQR, 0–1.25).	[66]
27 breast cancer patients	Breast cancer patients consumed a cocktail of plant extracts (cocoa, pomegranate, lemon, orange, grapeseed, and olive) plus resveratrol, providing 37 different phenolics (473.7 mg), theobromine and caffeine (19.7 mg) (n = 19) from diagnosis to surgical resection (6 ± 2 days). Control group did not consume extracts (n = 8).	Normal and tumour glandular breast tissues were extracted and further both non-hydrolyzed and hydrolyzed by enzymatic treatment ^1^. Quantitative analyses were performed by UPLC-ESI-QTOF-MS and carried out by peak area integration of their EIC using calibration curves of specific (free and conjugated metabolites) standards.	A total of 39 and 33 metabolites were identified in normal and tumour tissues, respectively. Some representative metabolites detected in tumour tissues (median and range, pmol/g) were urolithin-A-3-*O*-glucuronide (26.2; 3.2–66.5), 2,5-dihydroxybenzoic acid (40.2; 27.7–52.2), RSV-3-*O*-sulphate (86.4; 7.8–224.4), dihydroRSV-3-*O*-glucuronide (109.9; 10.3–229.4), and HP 3′-*O*-glucuronide 12.9 (2.7–14.1). No significantly different conjugation profiling was found in tumour vs. normal tissues. Overall, all compounds were mostly glucuronidated and(or) sulphated in tumour and normal tissues, 85.5% and 86.6%, respectively. Among these conjugates, the percentage of sulphated was slightly higher in tumour (42%) than in normal tissues (31%). Quantitative analysis after hydrolysis was only possible for 2,5- and 2,6-dihydroxybenzoic acids, HP, urolithin A, isourolithin A, and urolithin B.	[69]

^1^ enzymatic treatment was performed using β-glucuronidase/sulfatase enzymes. Abbreviations: DAD, diode array detection; EGCG, epigallocatechin-3-gallate; EIC, extracted ion chromatogram; GC, gas chromatography; HP, Hesperetin; HPLC, high performance liquid chromatography; IQR, interquartile range; MS, mass spectrometry; *O*-DMA, *O*-desmethylangolensin; RSV, resveratrol; UPLC-ESI-Q-TOF, ultra-performance liquid chromatography coupled to electrospray ionization quadrupole time-of-flight; UV, ultraviolet.

**Table 2:** In vitro studies conducted with relevant phenolic-derived metabolites on breast cancer (BC) models.

Breast Cellular Model	Compound Assayed	Dose/Duration	Main Outcomes	References
**Resveratrol**				
MCF-7, ZR-75-1, MDA-MB-231 (breast cancer cell lines) and MCF-10A (normal cell line)	RSV, RSV 3-sulph, RSV 4′-sulph, RSV 3,4′-disulph.	1–350 µM; 48 h	RSV: IC_50_ = 67.6–82.2 µM against all breast cancer cells; IC_50_ = 20 µM against MCF-10A. RSV 3-sulph: IC_50_ = 189–258 µM against MCF-7 and MDA-MB-231; IC_50_ = 228.3 µM against MCF-10A. RSV 4′-sulph and RSV 3,4′-disulph: no cytotoxic effect against breast cancer cells; IC_50_ = 202.4–202.9 µM against MCF-10A.	[111]
MCF-7	RSV, RSV 3-sulph, RSV 4′-sulph, RSV 3,5 disulph, RSV 3,4′-disulph, RSV 3,4′,5-trisulph.	340 µM; 72 h	Cytotoxic effect (only RSV and RSV 3-sulph).	[112]
*Saccharomyces cerevisiae* strain Y190 co-transformed with ERα LBD and hTif2 coactivator and MCF-7	RSV, RSV 3-gluc, RSV 3-glur, RSV 3-sulph, RSV 4′-sulph.	0.05–100 µM; 18–24 h	RSV 3-sulph showed anti-oestrogenic activity at 10 and 50 µM (with a marked preference for ERα at 10–100 µM), and weak oestrogenic activity. RSV showed oestrogenic activity in MCF-7 (5–10 µM).	[113]
MCF-7 and MDA-MB-231	RSV, RSV 3-glur, RSV 3-sulph, RSV 4′-sulph, DH-RSV, DH-RSV 3-glur.	10 and 50 µM; 3 and 7 days	↓Proliferation in MDA-MB-231 cells (only RSV and DH-RSV). Oestrogenic/anti-oestrogenic effect in MCF-7 (only RSV and DH-RSV).	[51]
MCF-7, MDA-MB-231 and MCF-10A (normal cell line)	RSV, RSV 3-glur, RSV 3-sulph, RSV 4′-sulph, DH-RSV, DH-RSV 3-glur.	0.5, 1, and 10 µM; 1–14 days	The effects were only observed in MCF-7 cells for all compounds: ↓Clonogenicity; cell cycle arrest; senescence induction; modulation of p53/p21 and p16/Rb pathways. In MDA-MB-231 cells (only RSV at 10 µM): ↓clonogenicity.	[109]
**Flavanones**				
MCF-7 and normal mammary epithelial cells H184B5F5/M10	Baicalein, baicalein 7-*O*-sulph, and baicalein-8-sodium sulphonate.	50, 100, and 200 µM; 24 h	Effects in MCF-7 cells: ↓cell viability; ↑LDH release; induction of cell cycle arrest; induction of morphological changes; ↑apoptosis; ↑ROS; ↑caspase-3, -9 activity. Effects on H184B5F5/M10 cells: No cytotoxic.	[114]
MCF-7 and MDA-MB-231	HP, HP 3′-glur, HP 7-glur, HP 3′-sulph, HP 7-sulph.	10 and 50 µM; 3 and 7 days	↓Proliferation in MDA-MB-231 cells (only HP at 50 µM). Oestrogenic/anti-oestrogenic effect in MCF-7 (only HP at 10 and 50 µM).	[51]
**Quercetin**				
MCF-7	Quer, Quer-3-*O*-gluc, Quer 3-*O*-glur, Quer 4′-*O*-sulph, Tamarixetin, Isorhamnetin.	50 µM; 48 h	↓Cell proliferation (no effect of Quer-3-*O*-gluc and Quer 3-*O*-glur).	[115]
*Saccharomyces cerevisiae expressing* ERα-Tif2 or ERβ-Tif2 and MCF-7	Quer, rutin, isoquercitrin, Quer 3-*O*-glur, Quer 3-*O*-sulph.	0.1–100 µM; 18–24 h	Quer 3-*O*-glur (IC_50_ = 2.1 ± 0.38 µM) and Quer (IC_50_ = 2.4 ± 0.93 µM) showed oestrogenic activity in MCF-7 cells. Quer 3-*O*-glur showed ERα-Tif2 (IC_50_ = 103 ± 2.24 µM) and ERβ-Tif2 (IC_50_ = 96 ± 1.2 µM) agonistic activity. Isoquercitrin showed similar ERβ-Tif2 agonistic activity than Quer 3-*O*-glur. Quer showed weak ERβ-Tif2 (IC_50_ = 5.3 ± 0.9 µM) agonistic activity.	[116]
MDA-MB-231	Quer 3-*O*-glur (alone or together with A and NA).	10^−10^–10^−4^ M (binding assay) and 0.01–1 µM (cell assay); 1–24 h	Quer 3-*O*-glur showed competitive binding of [^3^H]-NA to β2-AR (10^-4^–10^2^ µM). ↓ROS formation; ↓HMOX1, MMP-2, and MMP-9 gene expression; ↓intracellular cAMP level; ↓p-ERK 1/2 and p-P38; ↓RAS activation; ↓invasive capacity of MDA-MB-231; ↓MMP-9 activity.	[117]
MCF-10A (normal cell line)	Quer and Quer 3-*O*-glur (alone or together with 4-OHE_2_ and NA).	10^−10^–10^−4^ M (binding assay) and 0.05–10 µM (cell assay); 2 h	Quer and Quer 3-*O*-glur showed competitive binding of [^3^H]-NA to α_2_-AR (10^−4^–10^2^ µM). ↓γ-H2AX and AP sites activation.	[118]
MCF-7 and normal mammary epithelial cells H184B5F5/M10	Quer, Isorhamnetin, and Isorhamnetin 3-*O*-glucuronide.	25, 50, and 100 µM; 24–48 h	Effects in MCF-7 cells: ↓Cell viability; ↑LDH release: induction of cell cycle arrest; induction of morphological changes; ↑apoptosis; ↑ROS. Effects on H184B5F5/M10 cells: No cytotoxic effects.	[107]
MCF-7 and normal mammary epithelial cells H184B5F5/M10	Quer, Quer 3-*O*-glur, and Quer 3-*O*-sulph.	25, 50 and 100 µM; 24–48 h	Effects in MCF-7 cells: ↓cell growth; ↑LDH release; ↑ROS; ↑apoptosis; induction of cell cycle arrest; induction of morphological changes. Effects on H184B5F5/M10 cells: No cytotoxic effects.	[108]
**Urolithins**				
MCF-7 and MDA-MB-231	Uro-A, Uro-A 3-glur, Uro-A 8-glur, Uro-A 3-sulph, IsoUro-A, IsoUro-A 3-glur, IsoUro-A 9-glur, Uro-B, Uro-B 3-glur, Uro-B 3-sulph.	10 and 50 µM; 3 and 7 days	↓Proliferation in MCF-7 (Uro-A and IsoUro-A) and MDA-MB-231 (free forms and conjugates at 50 µM). Oestrogenic activity in MCF-7 (only free forms at 10 and 50 µM). Anti-oestrogenic activity in MCF-7 (Uro-A and its conjugates, IsoUro-A and its conjugates, and Uro-B at 10 and 50 µM).	[51]
**Catechin and epicatechin**				
MCF-7	Epi, Epi-3′-*O*-sulph, 3′-*O*-methyl-Epi, 4′-*O*-methyl-Epi, catechin, 3′-*O*-methyl-catechin.	100 µM; 48 h	↓Cell proliferation (only 4′-*O*-methyl-Epi).	[115]
**Isoflavones**				
MCF-7	GEN, GEN 4′-*O*-glur, GEN 7-*O*-glur, GEN 4′-*O*-sulph-7-*O*-glur, GEN 4′-*O*-sulph, GEN 7-*O*-sulph, GEN 4′,7-di-*O*-sulph, DAZ, DAZ 4′-*O*-glur, DAZ 7-*O*-glur, DAZ 4′-*O*-sulph-7-*O*-glur, DAZ 4′-*O*-sulph, DAZ 7-*O*-sulph, DAZ 4′,7-di-*O*-sulph, Glycitein, Glycitein 7-*O*-glur, DH-DAZ, DH-DAZ 7-*O*-glur, *O*-DMA, *O*-DMA 7-*O*-glur.	10–1000 µM; 24 h	Low stimulatory MCF-7 growth, β-gal induction, and binding to ERs: sulphates of GEN and glucuronides of glycitein and DH-DAZ. Moderate binding, but low (or lack) MCF-7 growth and β -gal induction: glucuronides of GEN, DAZ, and *O*-DMA. Moderate MCF-7 growth and β-gal induction, but low (or lack) binding: DAZ 4′-*O*-sulph-7-*O*-glur. *O*-DMA was the most active compound stimulating MCF-7 growth, binding to hERβ, and inducing β-gal.	[119]
MCF-7	GEN, GEN 7-*O*-glur, DAZ, DAZ 7-*O*-glur.	16 µM; 72 h	↑Cytotoxicity (only DAZ).	[120]
**Isoflavones**				
MCF-7	GEN, GEN 7-*O*-sulph, GEN 4′-*O*-sulph, DAZ, DAZ 7-*O*-sulph, DAZ 4′-*O*-sulph, equol, equol-7-sulph (in the presence of 4 × 10^−10^ M [2,4,6,7-^3^H] oestradiol).	10^−7^–10^−4^ M; 18–24 h (binding and gene expression assay) and 7 or 14 days (proliferation assay)	The sulphation in position 7 reduced the oestrogen capacity of GEN and equol in all assays. GEN 4′-*O*-sulph showed lower proliferative activity (at 1 and 10 µM), and similar or even higher binding affinity to ER (compared to GEN). DAZ 4′-*O*-sulph showed lower binding affinity to ER and increased proliferative activity (at 10 µM). DAZ 7-*O*-sulph showed similar or even higher affinity to ER and similar proliferative activity (at 1 and 10 µM) (compared to DAZ).	[121]
HS578T, MDA-MB-231, and MCF-7	Puerarin, GEN, DAZ, and mix of DAZ glucuronides/sulphates.	12.5–100 µM (free compounds) and 2.35 µM the mix of DAZ conjugates; 24–72 h	Effect of only with free compounds: (IC_50_ = 29–71 µM) ↓cell viability; induction of cell cycle arrest; ↑apoptosis; ↑caspase-3 activity. Effect of mix of DAZ conjugates: (IC_50_ = 2.35 µM) ↓cell proliferation; induction of cell cycle arrest; ↑apoptosis; ↑caspase-9, p53, p21, and Bax.	[122]
MCF-7, T47D, and MCF-10A (normal cell line)	GEN, GEN 7-*O*-sulph, GEN 4′-*O*-sulph, GEN 7-*O*-glur.	5.12 × 10^−3^–80 µM for GEN and 2.2–4.5 µM for conjugates; 3 days	Dissimilar effects of GEN cell proliferation: ↑ at low concentrations and ↓ at higher concentrations. GEN 7-*O*-glur stimulated cells growth. This effect was related to deconjugation. GEN 7-*O*-sulph, GEN 4′-*O*-sulph exerted no effects.	[106]
T47D and T47D-ERβ (tetracycline dependent ERβ expression)	GEN, GEN 7-*O*-glur, DAZ, DAZ 7-*O*-glur.	10^−5^–1000 µM; 48 h	Dissimilar effects on proliferation: ↓ at low concentrations and ↑ at higher concentrations. Lower proliferative potency than 17β-oestradiol: PC_50_ in T47D-wt: 17β-oestradiol = 4.2×10^−6^ µM; GEN = 0.19 µM; GEN 7-*O*-glur = 21.4 µM; DAZ = 0.186 µM; DAZ 7-*O*-glur = 107 µM. PC_50_ in T47ERβ: 17β-oestradiol = 2.45×10^−7^ µM; GEN = 0.2 µM; GEN 7-*O*-glur = 7.8 µM; DAZ = 0.035 µM; DAZ 7-*O*-glur = >400 µM.	[123]
**Isoflavones**				
MCF-7 and T47D	DAZ, DAZ 4′-*O*-sulph, DAZ 7-*O*-sulph, DAZ 4′,7-di-*O*-sulph, equol, *O*-DMA.	0.1, 1, and 10 µM; 1–24 h	Different effects on NGB: ↑equol, O-DMA, DAZ 7-*O*-sulph, DAZ 4′,7-di-*O*-sulph and ↓DAZ and DAZ 4′-*O*-sulph. Effects of DAZ, DAZ 4′-*O*-sulph and equol on protein phosphorylation: ↑p-ERα/ERα (all compounds); ↑p-Akt/Akt (only equol; 1 h treatment); ↑p-p38/p38 (all molecules; 1 and 24 h treatment). DAZ, DAZ 4′-*O*-sulph and equol preserve PAX activity in MCF-7 cells (↓NGB, ↑PARP-1 cleavage, and ↓cell number) in the presence of 17β-oestradiol. Effects of the mix of metabolites: ^1^ Mix of sulphates metabolites: ↓NGB by in the presence or absence of 1 µM DAZ; the mix of sulphates together with 1 µM DAZ preserve PAX effects (↑PARP-1 cleavage) in the presence of 17β-oestradiol. ^2^ Mix of gut metabolites: ↑NGB by equol + *O*-DMA (1 µM each) in the presence or absence of 1 µM DAZ; ↓PAX effects (↑PARP-1 cleavage) in the presence of 17β-oestradiol.	[124]

^1^ Mix of sulphate metabolites composition: DAZ 4′-*O*-sulph + DAZ 7-*O*-sulph + DAZ 4′,7-di-*O*-sulph (1 µM each); ^2^ Mix of gut metabolites composition: equol + *O*-DMA (1 µM each). Abbreviations: A, adrenaline; α_2_-AR, α_2_-adrenergic receptor; β_2_-AR, β_2_-adrenergic receptor; cAMP, cyclic adenosine monophosphate; DAZ, daidzein; DH-DAZ, dihydrodaidzein; DH-RSV, dihydroresveratrol; Epi, epicatechin; ER+, oestrogen receptor+; ERE-CAT, oestrogen response element chloramphenicol acetyl transferase; γ-H2AX, H2A histone family member X phosphorylated on Ser139; GEN, genistein; gluc, glucoside; glur, glucuronide; hERα, human oestrogen receptor alpha; HMOX1, heme oxygenase 1; HP, hesperetin; hTif2, coactivator receptor-interacting domain; LBD, ligand binding domain; LDH, lactate deshydrogenase; NA, noradrenaline; NGB, neuroglobin; *O*-DMA, *O*-desmethylangolensin; PAX, paclitaxel; PC, potency concentration; PARP-1, poly [ADP-ribose] polymerase 1; Quer, quercetin; ROS, reactive oxygen species; RSV, resveratrol; 4-OHE_2_, 4-hydroxy-oestradiol; sulph, sulphate.

**Table 3:** Clinical trials with dietary (poly)phenols or phenolic-containing foodstuffs in patients with breast cancer.

Cohort and Sample Size	Trial Design	Objective	Outcomes	References
Newly biopsy-diagnosed breast cancer patients (n = 32), with no hormone therapy	Design: Pre-surgery, randomized double-blind, placebo-controlled clinical trial. Product and dose: Patients (n = 19) consumed flaxseed (25 g/d) or placebo (n = 13). Follow-up: 37 and 39 days in the flaxseed and placebo groups, respectively.	Effect of flaxseed on tumour biomarkers.	Significant reductions in Ki-67 labelling index (34.2%) and in c-erbB2 expression, (71%), and an increase in apoptosis (30.7%) were observed in the flaxseed, but not in the placebo group.	[125]
Patients (n = 66) with tissue hardness due to radiotherapy for early breast cancer (at least 24 months prior to trial entry), with no active disease	Design: Phase II, placebo-controlled, randomized trial. Product and dose: Capsules containing a grape seed proanthocyanidin extract 100 mg three times a day orally, or placebo. Follow-up: 6 months.	Effect on the surface area of palpable breast induration after 12 months. Secondary endpoints: change in photographic breast appearance and patient self-assessment of breast hardness, pain and tenderness.	No significant difference between treatment and control groups in terms of external assessments of tissue hardness, breast appearance or patient self-assessments of breast hardness, pain or tenderness.	[136]
Patients (n = 14) with metastatic breast cancer	Design: Open label, phase I, non-controlled trial. Product and dose: Intravenous docetaxel plus oral curcumin (escalated dose until toxicity limit is reached). Follow-up: Docetaxel 100 mg/m^2^ was administered every 3 weeks (w) for six cycles. Curcumin was given orally for 7 consecutive days (d) (from d-4 to d+2) for six cycles (a total of 63 cycles).	Establishment of the maximal tolerated dose (MTD) of oral curcumin plus intravenous docetaxel.	MTD of curcumin was 8 g/day, in combination with docetaxel 100 mg/m^2^ administered every 3 w for six cycles. Recommended curcumin dose: 6 g/d for 7 consecutive d every 3 w in combination with a standard dose of docetaxel. Decrease of plasmatic CEA and VEGF.	[133]
Patients (n = 40) with resected stage I-III ER- and PR- breast cancer with no active disease	Design: Randomized, phase IB, double-blinded, placebo-controlled, and dose-escalation study. Product and dose: Capsules (green tea extract) containing EGCG (treated group, n = 30). Daily oral dose was 800 mg (n = 16), 1200 mg (n = 11), and 1600 mg (n = 3) EGCG. Control group (n = 10): placebo. Follow-up: 6 months.	Establishment of the MTD for EGCG.	MTD was 1200 mg/d EGCG for 6 months. No significant change in SHBG, IGF-1, IGFBP-3, ER, Ki-67 proliferation index or mammographic density	[129]
Patients (n = 28) with ductal carcinoma in situ or primary invasive stage I or II breast cancer	Design: Pre-surgery, controlled study. Product and dose: 3 capsules/d (green tea extract) containing EGCG (treated group, n = 13). Daily oral dose was 940 mg EGCG. Control group (n = 15): no capsules. Follow-up: Average duration of green tea intake was 35 days.	Short-term effects of green tea supplementation on cancer biomarkers.	Significant decrease of Ki-67 in the tea group, but only in normal tissue. No effects on apoptosis (caspase-3), and angiogenesis (CD34) markers in benign or malignant tissue.	[128]
Patients (n = 30) with non-inflammatory breast cancer or carcinoma in situ and prescribed RT without concurrent chemotherapy	Design: Randomized, double-blind, placebo-controlled clinical trial. Product and dose: Patients (n = 14) consumed 6 g/d curcumin extract (a daily dose of 4.7 g curcumin, 0.9 g demethoxycurcumin, and 0.15 g bisdemethoxycurcumin) or placebo (n = 16). Follow-up: 6 months (30 RT sessions)	Effect of curcumin to reduce RDS.	Significant reduction of RDS and moist desquamation at the end of treatment vs. placebo (mean RDS = 2.6 vs. 3.4; and 28.6% vs. 87.5%; respectively).	[134]
Postmenopausal women (n = 24) with newly-diagnosed, and resectable, ER+ breast cancer	Design: Pre-surgery, 2 × 2 factorial, randomized, placebo-controlled trial. Product and dose: 25 g/d ground flaxseed + 1/d placebo pill (n = 6); 1 mg/d anastrozole (aromatase inhibitor) (n = 7); 25 g/d ground flaxseed + 1 mg/d anastrozole (n = 6); or 1/d placebo pill control (n = 5). Follow-up: Mean of 18.8 days.	Effects of flaxseed and the aromatase inhibitor, anastrozole, on steroid hormones and tumour-related biomarkers.	Mean ERβ expression was approximately 40% lower from pre- to post-intervention in the flaxseed (FS) + anastrozole (AI) group only. Significant negative association for androstenedione in the FS + AI group vs. placebo, and for dehydroepiandrosterone with AI treatment.	[126]
Patients (n = 34) with resected stage I-III ER- and PR- breast cancer with no active disease. (Ancillary study to that of Crew et al. [129]	Design: Randomized, phase IB, double-blinded, placebo-controlled, and dose-escalation study. Product and dose: Capsules (green tea extract) containing EGCG (treated group, n = 26). Daily oral dose was 800 mg (n = 14), 1200 mg (n = 11), and 1600 mg (n = 1) EGCG. Control group (n = 8): placebo. Follow-up: 6 months	Effect of EGCG on cancer biomarkers risk.	Significant transient decrease of serum HGF (only after 2 months of treatment) in EGCG consumers. No significant effects on VEGF, serum cholesterol and triglycerides, oxidative damage, and inflammatory biomarkers.	[130]
Patients (n = 12) with newly diagnosed breast cancer, ER+, not eligible for neoadjuvant treatment	Design: Pre-surgery (non-controlled) dietary intervention. Product and dose: Silybin-phosphatidylcholine complex (2.8 g/d) given orally. Follow-up: 4 w before surgery.	Effects on NO, IGF-1 and Ki-67.	No effects on NO, IGF-1 and Ki-67 were observed.	[65]
Patients (n = 12) with newly diagnosed breast cancer, not eligible for neoadjuvant treatment.	Design: Pre-surgery (non-controlled) dietary intervention. Product and dose: Catechin (65.1 mg/d) and EGCG (44.9 mg/d) given orally (300 mg tea extract). Follow-up: 4 w before surgery.	Effect on cell proliferation, angiogenesis, oxidative stress, chronic inflammation, and adiposity-related endocrine mechanism.	Significant increase of testosterone. No effect in the rest of markers.	[66]
Patients (n = 578) with non-inflammatory breast cancer or carcinoma *in situ*, and prescribed fractionated radiation therapy (RT) without concurrent chemotherapy	Design: Phase II, randomized, double-blind, placebo-controlled clinical trial. Product and dose: Patients (n = 283) consumed either 6 g/d curcumin extract (a daily dose of 5.4 g curcumin, 0.48 g dimethoxy curcumin, and 0.12 g bisdemethoxy curcumin), or placebo (n = 295). Follow-up: 6 months (30 RT sessions).	Confirmatory study on the effect of curcumin to reduce radiation dermatitis severity.	Curcumin did not reduce radiation dermatitis severity at the end of RT compared to placebo.	[135]
ER+ and(or) PR+ postmenopausal women (n = 45) with resected breast cancer at early stage (with no active disease), and receiving adjuvant hormonal therapy	Design: Open-label, single-arm (no placebo-controlled). Product and dose: Three daily capsules, containing 460 mg of fish oil (EPA and DHA), 125 mg of olive extract (12.5 mg hydroxytyrosol), and 50 mg extract of curcumin (47.5 mg curcuminoids) Follow-up: 30 days.	Effect on inflammation and pain.	Significant decrease of plasma CRP (from 8.2 ± 6.4 mg/L at baseline to 5.3 ± 3.2 mg/L), and pain (21.5%) after 30 days.	[139]
Breast cancer patients (n = 10)	Design: Pre-surgery, two arms, controlled study. Product and dose: Patients (n = 5) consumed walnuts (around 60 g/d) or not (i.e., controls, n = 5). Follow-up: About 15 days.	Effect of walnut consumption on gene expression in breast cancer tissue.	Significant change of 456 genes in the tumour due to walnut consumption. Activation of pathways that promote apoptosis and cell adhesion, and inhibition of pathways that promote cell proliferation and migration.	[138]
Patients (n = 81) with histologically confirmed operable ER+ breast cancer, with no distant metastasis, and receiving tamoxifen.	Design: Randomized, double-blind, and placebo-controlled trial. Product and dose: Patients (n = 42) consumed a red clover extract (80 mg isoflavones/d) or placebo (n = 39). Follow-up: 2 years	Effect of isoflavones from red clover extract and lifestyle change to reduce side-effects of tamoxifen treatment.	The reductions in BMI and waist circumference were significantly greater in the treatment than placebo group. No differences between groups in the rest of determinations: MRS, HDLc, insulin, total cholesterol, LDLc, triglycerides, insulin resistance, sex hormone levels, endometrial thickness and breast density.	[137]

Abbreviations: CEA, carcinoembryonic antigen; c-erbB2 (or HER2), Humanized epidermal growth factor receptor 2; DHA, docosahexanoic acid; EGCG, epigallocatechin gallate; EPA, eicosapentaenoic acid; ER: oestrogen receptor; HGF, hepatocyte growth factor; IGF-1, insulin-like growth factor 1; IGFBP-3, Insulin-like growth factor binding protein-3; MRS, menopausal rating score; MT, malignant tissue; NT, normal tissue; PR: progesterone receptor; RDS, Radiation Dermatitis Severity score; RT, radiation therapy; SHBG, Sex hormone binding globulin; VEGF, vascular endothelial growth factor.

## Supplementary Materials

Supplementary materials can be found at https://www.mdpi.com/1422-0067/21/16/5718/s1. Table S1: Dietary phenolic-derived metabolites identified in breast tissues in animal model studies. Table S2: Animal studies conducted with dietary phenolics and/or phenolic-rich extracts on BC models.

## Abbreviations

BC	Breast Cancer
DMBA	7,12-Dimethylbenz[a]anthracene
EGCG	Epigallocatechin Gallate
ER	Oestrogen Receptor
HER2	Human Epidermal growth factor Receptor 2
MTD	Maximum Tolerability Dose
PR	Progesterone Receptor
RSV	Resveratrol
TNBC	Triple Negative Breast Cancer
