# Supplementary material for: Dietary Phenolics against Breast Cancer. A Critical Evidence-Based Review and Future Perspectives
Source: Int J Mol Sci. 2020 Aug 10;21(16):5718. doi: 10.3390/ijms21165718 (PMC7461055; doi:10.3390/ijms21165718)
Supplement: Supplementary file 1 [file ijms-21-05718-s001.zip › ijms-890202-sup-proofed/Table S1.pdf]

Table S1. Dietary phenolic-derived metabolites identified in breast tissues in animal model studies.

Animal model	Diet/compound administration	Extraction and analytical conditions	Identified and(or) quantified phenolic metabolites	References
**Isoflavones**				
Female Sprague-Dawley rats (n=5 per group)	Genistein-supplemented diet (25 and 250 mg/kg) from conception to day 21 post-partum.	Mammary tissues were hydrolysed both with and without enzymatic treatment[1] for analyses of free or total genistein, respectively. Quantitative analyses were performed by HPLC/MS using genistein standard.	Free and total genistein (318±56 and 440±129 pmol/g, respectively) at 7 days, after 250 mg/kg of genistein in diet. Free and total genistein (304±13 and 370±36 pmol/g, respectively) at 21 days, after 250 mg/kg of genistein in diet.	[1]
Female Sprague-Dawley rats (n=6 per group)	Genistein-supplemented diet (5, 100 and 500 μg/g) for 7 days, since weaning to day 140.	Mammary glands tissues were hydrolysed both with and without enzymatic treatment[1] for analyses of free or total genistein, respectively. Quantitative analyses were performed by HPLC/MS-MS using genistein standard.	Free genistein: 0.12±0.02 (41% of total) and 1.18±0.22 (49% of total) pmol/mg, after 100 and 500 μg/g dose, respectively).	[2]
7-day-old offspring of dams Sprague-Dawley rats (n=6)	Daidzein-supplemented diet (250 mg/kg) for 7 days, starting at conception	Mammary glands tissues were hydrolysed both with and without enzymatic treatment[1] for analyses of free or total daidzein or equol, respectively. Quantitative analyses were performed by HPLC/MS using daidzein and equol standards.	Free and total daidzein (400±120 and 407±105 pmol/g, respectively). Total equol (27±8 pmol/g). No free equol was detected.	[3]
Female BALB/cA Jcl-nu athymic mice bearing MDA-MB-231 tumours (n=5)	Genistein combined polysaccharide (GCP™)-supplemented diet (1%) for 28 days after the tumour inoculation. GCP™ contained 116±8.4mg of genistein, 28.5±5.4mg of daidzein, 13.5 ± 2.6mg of glycitein and about 3% of insoluble polysaccharides.	Tumour and normal tissues were hydrolysed both with and without enzymatic treatment[1] for analyses of genistein content. Quantitative analyses were performed by HPLC using genistein, daidzein and glycitein standards.	The amount of aglycone genistein in GCPTM-treated tumour tissues (78.42±23.17 μg/mg protein) was significantly higher (around 3-fold) than those in normal liver and colon tissues. The percentages of aglycone genistein in total genistein (aglycone and conjugated) were about 80% in tumour tissues and about 20% in normal tissues. Other aglycone isoflavones, including daidzein and glycitein, were detectable in tumours, but their mean concentrations were lower than 1 μg/mg protein.	[4]

Female Sprague-Dawley rats (n=6)	Diet formulated with soy protein isolate (containing 0.27 mg/g of genistein and 0.21 mg/g of daidzein) for 4 days.	Mammary glands tissues were hydrolysed both with acid (ammonium acetate buffer plus hydrochloric acid) and enzymatic treatment[1] for analyses of total genistein and daidzein. Analyses were performed by LC-MS. Isoflavone concentrations in tissues were normalised with biochanin A.	Total genistein (0.11±0.01 and 0.03±0.01 nmol/g, after acid and enzymatic hydrolysis, respectively). Total daidzein (0.17±0.01 and 0.02±0.01 nmol/g, after acid and enzymatic hydrolysis, respectively).	[5]
3-year-old lactating ewe (n=2)	Diet containing 50% red clover silage (variety Pawera) for one month with a daily intake of 157.6 mg/kg bw of total isoflavones (81.8 formononetin, 64.8 biochanin A, 7.6 genistein, and 2.9 daidzein).	Mammary glands tissues were hydrolysed both with and without enzymatic treatment[1] to determine the fraction of equol and daidzein present as aglycones. Quantitative analyses were performed by HPLC-Coularray and LC/MS-MS using specific standards.	Total isoflavones (<10 nmol/g of daidezin and equol, mainly present as glucuronides). Formononetin and biochanin A were not detected. Free daidzein was not detected. Free equol (1.9±1.9% of total equol detected).	[6]
Female Sprague-Dawley rats (n=5)	Isoflavone-supplemented diet (containing an alcohol-washed soy protein isolate plus, providing a total of 1,047 mg/kg of total isoflavones) for 28 days.	Mammary gland tissues were hydrolysed by enzymatic treatment[1]. Quantitative analyses were performed by liquid Waters Acquity UPLC/MS using specific standards.	Total equol (1.12±0.06 nmol/g), total genistein (0.11±0.02 nmol/g) after enzymatic treatment. Daidzein and glycitein were not detected.	[7]
Female pigs (n=15 per group)	Soy-based commercial diet containing 582.8 mg/kg of isoflavones (soy diet) or soy diet supplemented with a daily dose of 2.3 g of genistein (soy+gen diet) for 93 days.	Mammary gland tissues were hydrolysed by enzymatic treatment[1]. Quantitative analyses were performed by Waters Acquity UPLC/MS using specific standards.	Total daidzein (0.07±0.02 and 0.13±0.02 nmol/g in soy and soy+gen diet, respectively). Total equol (0.12±0.03 and 0.19±0.04 nmol/g in soy and soy+gen diet, respectively). Total genistein (0.03±0.00 and 0.15±0.01 nmol/g in soy and soy+gen diet, respectively). Glycitein was not detected.	[7]
**Green tea catechins**				
Female nude mice (n=4 per group)	Green tea extract was assigned to drinking water (0.625 g/L, 1.25 g/L and 2.5 g/L) for 4 days.	Mammary fat pads were hydrolysed by enzymatic treatment[1]. Quantitative analyses were performed by HPLC using epicatechin (EC), epigallocatechin (EGC) and epigallocatechin-3-gallate (EGCG) standards.	EGCG (around 28, 38 and 45ng/g)[2] was the catechin type with the highest concentration, followed by EC (around 4, 8 and 30ng/g)[2], then ECG (around 2, 3 and 8ng/g)[2] after the 0.625, 1.25 and 2.5g/L dose, respectively.	[8]

Heterozygous transgenic female mice (Tag mice develop palpable tumours) (n=4)	Green tea catechins were assigned to drinking water either at 0.05% (w/v) for 7 weeks.	Mammary tumour tissues were hydrolysed both with and without enzymatic treatment[1]. Quantitative analyses were performed by HPLC/MS-MS using specific standards (EC, EGC and EGCG), but not for conjugated metabolites.	Only EGC was quantified (range 32-104 pmol/g) after hydrolysis, whereas its methyl and sulphate metabolites were also identified in non-hydrolysed samples. There was no evidence of the presence of EGCG and EC.	[9]
		**Prenylflavonoids**		
Female ovariectomized Sprague-Dawley rats (n=24)	8-Prenylnaringenin (8-PN) at three doses (0.4, 4 and 40 mg/kg) or isoxanthohumol (IX, control) were administered by intraperitoneal injection (n=6 each group).	Mammary glands (24 h post-dose) were non-hydrolysed by enzymatic treatment[1]. Qualitative and quantitative analyses were performed by LC/MS-MS using 8-PN standard.	8-PN was detected in mammary gland tissue from all animals administered 8-PN, but not from those given IX. 8-PN (0.014±0.01, 0.047±0.05 and 0.6±0.4 μg/g) at doses 0.4, 4, 40 mg/kg of 8-PN, respectively. 8-PN glucuronide was also detected, but not quantified. The relative levels of 8-PN glucuronides were considerably higher than free 8-PN.	[10]
Female Sprague-Dawley rats (n=5 per group)	Control diet plus xanthohumol (XN, 100 mg/kg bw per day) administered by subcutaneous injection or experimental diet containing powdered hop extract (7.5 g/kg bw per day) for 4 days.	Mammary glands were non-hydrolysed by enzymatic treatment[1]. Qualitative and quantitative analyses were performed by LC/MS-MS using XN standard but not its metabolites.	In general, 8-PN and 6-PN were higher than XN, predominantly found in the form of glucuronide conjugates. 8-PN glucuronide was higher than that of 6-PN glucuronide. The quantitative analysis of the free XN aglycones: 0.30±0.41 μg/g after XN injection and 0.05±0.04 μg/g after hop extract intake.	[11]
		**Flavanones**		
Female obese ovariectomized C57BL/6 mice bearing E0771 cells (n=10 per group)	High-fat diet with low naringenin (LN; 1% naringenin) or high-fat diet with high naringenin (HN; 3% naringenin) for 2 weeks and 3 more weeks after transplanted tumour in mammary adipose tissue.	Thoracic mammary adipose tissue and tumour were hydrolysed by enzymatic treatment[1]. Quantitative analyses were performed by LC/MS-MS using naringenin standard.	Total naringenin concentrations were about 3-fold higher in HN mice compared to LN mice. HN mice: total naringenin in tumour (5.68±1.45 μmol/kg) and mammary adipose tissue (2.49±0.34 μmol/kg). LN mice: total naringenin in (2.05±0.87 μmol/kg) and mammary adipose tissue (0.75±0.14 μmol/kg).	[12]
		**Resveratrol**		

Female Sprague-Dawley rats (n=15 per group)	Resveratrol (RSV, 100 mg/kg bw) alone or in combination with celecoxib (Cele, 0.167%) was orally administered daily for 2 weeks and 16 more weeks after first dose of N-methyl-N-nitrosourea to induce mammary carcinogenesis.	Breast tumour tissues were non-hydrolysed by enzymatic treatment[1]. Quantitative analyses were performed by HPLC using specific standards for RSV and its conjugated metabolites.	The concentration of free RSV did not differ between both groups (RSV group: 0.53±0.19 ng/mg vs. RSV+Cele group: 0.57±0.13 ng/mg).<br>RSV is metabolized into RSV-3-*O*-glur mainly, and RSV-4'-*O*-glur and RSV-3-*O*-sulph in lower amounts (not detailed).	[13]
Female BALB/c nude mice bearing MCF-7/Adr tumours (n=18)	Free RSV (20 mg/kg), RSV encapsulated in liposomes (RSV-LIP) or co-encapsulated with 8 mg/kg paclitaxel (RSV-PTX) were IV administered via the tail vein every two days.	Mice were sacrificed at 24 h, 48 h, and 2 weeks after treatment initiation (n = 6 at each time point). Tumours (volume, 50 mm^3 to 100 mm^3) were examined for the concentration RSV by HPLC (no detailed).	Free RSV concentration[2] (around 2, 3 and 5μg/g at 24 h, 48 h, and 2 weeks, respectively).<br>The RSV concentration in the tumour from the RSV-PTX or RSV-LIP treatment was 1.6-fold to 5-fold that of the free RSV.	[14]
Female ovariectomized C57BL/6 mice bearing M-Wnt murine mammary tumour cells (n=7)	Diet-induced obesity supplemented with RSV (5 g/kg diet, 0.5% wt/wt) for 6 weeks and 5 more weeks after transplanted tumour.	Tumours were non-hydrolysed by enzymatic treatment[1]. Quantitative analyses were performed by Waters Acquity UPLC using the peak area of the fluorescence response of RSV and piceatannol (as an internal standard).	RSV levels in tumours samples (range 157.7—661.5 nmol/g).	[15]
		**Lignans**		
Adult pigs (n=17)	Diet supplemented with high-fibre wheat breads low in lignans (124.0 μg/100g dry matter) (n=8) or high-fibre rye breads (n=9) rich in lignans (4152.7 μg/ 100g dry matter) for 9 days.	Breast tissues were hydrolysed by enzymatic treatment[1]. Quantitative analyses were performed by HPLC-CEAD using enterolactone standard for aglycones.	Total enterolactone was found in lower concentrations in the breast tissue of the wheat-fed pigs (0.3±0.2pmol/g) than rye-fed pigs (25.8±4.1 pmol/g).	[16]
Female wild-type and knockout Abcg2/- mice<br>(n=7-8 per group)	Diet supplemented with 1% of lignan-rich extract from flaxseed hulls (2 mg/g secoisolariciresinol diglucoside (Sec-Dig) (content in diet) for one week.	Mammary glands were non-hydrolysed by enzymatic treatment[1]. Quantitative analyses were performed by UPLC-QTOF-MS using specific standards for aglycones, but not conjugated forms.	Both enterodiol and secoisolariciresinol were similarly accumulated in the mammary gland in both mice groups (around 400 ng/g and 70 ng/g, respectively). Sec-Dig levels were not detected.<br>Enterolactone concentration was higher in wild-type that Abcg2-/- mice (around 180 ng/g and 80 ng/g, respectively). Enterodiol-glur, enterolactone-glur and secoisolariciresinol-glur were identified,	[17]

			higher in Abcg2-/- than in wild-type mice.	
**Curcumin**				
Female Kunming mice bearing EMT6 tumours (n=6 per group)	Curcumin-loaded micelles or free curcumin (CUR) were IV administered at doses of 10 mg/kg daily for nine day.	Kunming mice were monitored by fluorescence imaging of tumours after 2, 6, 12 and 24 h postinjection. Biodistribution information was semiquantitatively illustrated using the Winmi software.	Minor amount of free CUR was found at the tumour target site, which showed a moderate increase during the first 6 h, but the amount decreased rapidly thereafter, reaching trace level after 24 h. The CUR amounts were considerably higher at the target tumour site after the administration of CUR -micelles than free CUR administration.	[18]
BALB/c nude mice bearing MCF-7 tumours	CUR (10 mg) in combination with doxorubicin (2 mg/mL) alone or co-encapsulated in transferrin decorated nanoparticles (NPs) were administered by IV via the tail vein.	Mice were sacrificed at 10 min, 1, 8, 24 and 48 h after IV injection. Tissues were initially weighed and homogenized with physiological saline to determine the amount of CUR by HPLC (no detailed).	The CUR concentration in tumour was higher in NPs than alone and were relatively stable at all time-points from 1 to 48 h after injection (around 18-22 μg/g and 40-50μg/g, respectively)[2].	[19]
Female wild-type Balb/c and bearing 4T1 tumours mice (n=4-5 per group)	Oral administration of CUR self-microemulsifying drug delivery system (100mg/kg) (SMEDDS, 30 mg of CUR solubilized in 1 g of blank SMEDDS) once-daily for two weeks after the tumour reached 400 mm^3.	Mammary tissues were non-hydrolysed by enzymatic treatment[1]. Quantitative analyses were performed by HPLC/MS-MS using specific standards.	Nearly 15-fold–higher levels of CUR (149.8±18.0 ng/g) were present in 4T1 tumours compared with those in the mammary tissue of healthy mice (10.5±2.1 ng/g). CUR-glur concentrations in tumours were much lower (<15 ng/g) in both groups.	[20]

Female Balb/c bearing 4T1 or TuBo tumours mice (high and low β-glucuronidase activities, respectively) and wild-type Balb/c (n=15 per group)	Pharmacokinetics of CUR and CUR-glur were evaluated following either oral dosing of CUR SMEDDS (100mg/kg) in both tumour-bearing models, and IV injection of CUR-glur (2 mg/kg) in all mice models after the tumour volumes reached 400 mm^3.	Mammary tissues were non-hydrolysed by enzymatic treatment at 0.5, 1, 2, 4 and 8 h post oral dose and at 0.25, 0.5, 1, 2, 4, 8 and 24 h post IV dose (n=3-4 each time point). Quantitative analyses were performed by HPLC/MS-MS using specific standards.	Oral dose: The C_{max} for CUR in 4T1 tumours was significantly higher than that in TuBo tumours (105±18 ng/g vs. 63±18 ng/g). CUR was present at a significantly higher concentration than CUR-glur (<20 ng/g) in the tumour tissues in both tumour-bearing mice models.<br>IV delivery: In both models, CUR levels in tumours started off low at early time points (0.25–4 h) but then increased to a steady concentration that plateaued (>8h) at around 30-40 ng of CUR/g, whereas its glucuronide decreased (<10 ng/g). In wildtype Balb/c mice, the CUR levels in healthy mammary tissue were significantly lower (3–4-fold difference) than in 4T1 and TuBo tumours.	[20]
		**Mix of phenolics**		
Female Sprague-Dawley rats (n=56)	Group mix (n=28): one capsule containing 37 phenolics (6.7 mg pomegranate, 6.7 mg olive, 6.7 mg cocoa, 2.2 mg orange, 2.2 mg lemon, 2.2 mg grapeseed and 2.2 mg RSV, 0.3 mg theobromine and 0.007 mg caffeine) was administered by gavage to each fasting animal. RSV group (n=28): a capsule containing 2.2 mg RSV.	Mammary tissues were both non-hydrolysed and hydrolysed by enzymatic treatment[1] at 0.5, 1, 2, 4, 6, 10, and 16 h after the capsule administration. Quantitative analyses were performed by UPLC-ESI-QTOF-MS and carried out by peak area integration of their EIC using calibration curves of specific (free and conjugated metabolites) standards.	Conjugated derived (but not free) metabolites from RSV, dihydroresveratrol (DH-RSV), hesperetin (HP), urolithins (Uro), and hydroxytyrosol (Hytyr) were detected.<br>Cmax of Hytyr glur (77.2±38.7 pmol/g), HP-3-glur (2.1±0.7 pmol/g), HP-7-glur (2.3±0.3 pmol/g), Uro-A 3-glur (6.7±3.8 pmol/g), RSV-3-glur (668.4±126.9 and 1,262±597.0 pmol/g in group mix and RSV group, respectively), RSV-3-glur (61.6±28.3 and 112.2±40.9 pmol/g in group mix and RSV group, respectively), and DH-RSV (206.3±113.1 and 406.2±331.6 pmol/g in group mix and RSV group, respectively).	[21]

[1] enzymatic treatment was performed using β-glucuronidase/sulfatase enzymes; [2]Quantitative values were estimated from graphics present in the original paper.
Abbreviations: 8-PN, 8-Prenylnaringenin; CUR, curcumin; EC, epicatechin; EGC, epigallocatechin; EGCG, epigallocatechin-3-gallate; EIC, extracted ion chromatogram; glur, glucuronide; HPLC, high performance liquid chromatography; IV, intravenous; IX, isoxanthohumol; MS, mass spectrometry; RSV, resveratrol; Sec-Dig, secoisolariciresinol diglucoside; SMEDDS, self-microemulsifying drug delivery system; UPLC-ESI-Q-TOF, ultra-performance liquid chromatography coupled to electrospray ionization quadropole time-of-flight; sulph, sulphate; XN, xanthohumol.

References

1. Fritz, W. Dietary genistein: perinatal mammary cancer prevention, bioavailability and toxicity testing in the rat. *Carcinogenesis* **1998**, *19*, 2151–2158, doi:10.1093/carcin/19.12.2151.
2. Chang, H.C.; Churchwell, M.I.; Delclos, K.B.; Newbold, R.R.; Doerge, D.R. Mass spectrometric determination of genistein tissue distribution in diet-exposed Sprague-Dawley rats. *J. Nutr.* **2000**, *130*, 1963–1970, doi:10.1093/jn/130.8.1963.
3. Lamartiniere, C.A.; Wang, J.; Smith-Johnson, M.; Eltoum, I.E. Daidzein: bioavailability, potential for reproductive toxicity, and breast cancer chemoprevention in female rats. *Toxicol. Sci.* **2002**, *65*, 228–238, doi:10.1093/toxsci/65.2.228.
4. Yuan, L.; Wagatsuma, C.; Yoshida, M.; Miura, T.; Mukoda, T.; Fujii, H.; Sun, B.; Kim, J.H.; Surh, Y.J. Inhibition of human breast cancer growth by GCP (genistein combined polysaccharide) in xenogeneic athymic mice: involvement of genistein biotransformation by beta-glucuronidase from tumor tissues. *Mutat. Res.* **2003**, *523–524*, 55–62, doi:10.1016/s0027-5107(02)00321-4.
5. Gu, L.; Laly, M.; Chang, H.C.; Prior, R.L.; Fang, N.; Ronis, M.J.; Badger, T.M. Isoflavone conjugates are underestimated in tissues using enzymatic hydrolysis. *J. Agric. Food Chem.* **2005**, *53*, 6858–6863, doi:10.1021/jf050802j.
6. Urpi-Sarda, M.; Morand, C.; Besson, C.; Kraft, G.; Viala, D.; Scalbert, A.; Besle, J.M.; Manach, C. Tissue distribution of isoflavones in ewes after consumption of red clover silage. *Arch. Biochem. Biophys.* **2008**, *476*, 205–210, doi:10.1016/j.abb.2008.05.002.
7. Gilani, G.S.; Farmer, C.; Dyck, M.; Robertson, P.; Dahiya, J.; Sepehr, E.; Fan, L.; Nicolidakis, H.; Curran, I.; Cooke, G.M. Distribution of isoflavones in samples of serum, liver and mammary glands of rats or pigs fed dietary isoflavones. *Ann. Nutr. Metab.* **2011**, 58, 171–180, doi:10.1159/000328771.
8. Sartippour, M.R.; Pietras, R.; Marquez-Garban, D.C.; Chen, H.-W.; Heber, D.; Henning, S.M.; Sartippour, G.; Zhang, L.; Lu, M.; Weinberg, O.; et al. The combination of green tea and tamoxifen is effective against breast cancer. *Carcinogenesis* **2006**, *27*, 2424–2433, doi: 10.1093/carcin/bgl066.
9. Kaur, S.; Greaves, P.; Cooke, D.N.; Edwards, R.; Steward, W.P.; Gescher, A.J.; Marczylo, T.H. Breast Cancer Prevention by Green Tea Catechins and Black Tea Theaflavins in the C3(1) SV40 T,t Antigen Transgenic Mouse Model Is Accompanied by Increased Apoptosis and a Decrease in Oxidative DNA Adducts. *J. Agric. Food Chem.* **2007**, *55*, 3378–3385, doi:10.1021/jf0633342.
10. Overk, C.R.; Guo, J.; Chadwick, L.R.; Lantvit, D.D.; Minassi, A.; Appendino, G.; Chen, S.N.; Lankin, D.C.; Farnsworth, N.R.; Pauli, G.F.; et al. In vivo estrogenic comparisons of Trifolium pratense (red clover), Humulus lupulus (hops), and the pure compounds isoxanthohumol and 8-prenylnaringenin. *Chem. Biol. Interact.* **2008**, *176*, 30–39, doi: 10.1016/j.cbi.2008.06.005.
11. Dietz, B.M.; Hagos, G.K.; Eskra, J.N.; Wijewickrama, G.T; Anderson, J.R.; Nikolic, D.; Wright, B.; Chen, S-N.; Pauli, G.F.; van Breemen, R.B.; et al. Differential regulation of detoxification enzymes in hepatic and mammary tissue by hops (Humulus lupulus) in vitro and in vivo. *Mol. Nutr. Food Res.* **2013**, *57*, 1055–1066, doi:10.1002/mnfr.201200534.
12. Ke, J.-Y.; Banh, T.; Hsiao, Y.-H.; Cole, R.M.; Straka, S.R.; Yee, L.D.; Belury, M.A. Citrus flavonoid naringenin reduces mammary tumor cell viability, adipose mass, and adipose inflammation in obese ovariectomized mice. *Mol. Nutr. Food Res.* **2017**, *61*, 1600934, doi:10.1002/mnfr.201600934.
13. Kisková, T.; Jendželovský, R.; Rentsen, E.; Maier-Salamon, A.; Kokošová, N.; Papčová, Z.; Mikeš, J.; Orendáš, P.; Bojková, B.; Kubatka, P.; et al. Resveratrol enhances the chemopreventive effect of celecoxib in chemically induced breast cancer in rats. *Eur. J. Cancer Prev.* **2015**, *23*, 506–513, doi:10.1097/CEJ.0000000000000083.
14. Meng, J.; Guo, F.; Xu, H.; Liang, W.; Wang, C.; Yang, X.D. Combination Therapy using Co-encapsulated Resveratrol and Paclitaxel in Liposomes for Drug Resistance Reversal in Breast Cancer Cells in vivo. *Sci Rep.* **2016**, *6*, 22390, doi:10.1038/srep22390.
15. Rossi, E.L.; Khatib, S.A.; Doerstling, S.S.; Bowers, L.W.; Pruski, M.; Ford, N.A.; Glickman, R.D.; Niu, M.; Yang, P.; Cui, Z.; et al. Resveratrol inhibits obesity-associated adipose tissue dysfunction and tumor growth in a mouse model of postmenopausal claudin-low breast cancer. *Mol Carcinog.* **2018**, *57*, 393–407, doi:10.1002/mc.22763.
16. Lærke, H.N.; Mortensen, M.A.; Hedemann, M.S; Bach-Knudsen, K.E.; Penalvo, J.L.; Adlercreutz, H. Quantitative aspects of the metabolism of lignans in pigs fed fibre-enriched rye and wheat bread. *Br. J. Nutr.* **2009**, *102*, 985–994, doi:10.1017/S0007114509344098.
17. García-Mateos, D.; García-Villalba, R.; Marañón, J.A.; Espín, J.C.; Merino, G.; Álvarez, A.I. The Breast Cancer Resistance Protein (BCRP/ABCG2) influences the levels of enterolignans and their metabolites in plasma, milk and mammary gland. *J. Funct. Foods* **2017**, *35*, 648–654, doi:10.1016/j.jff.2017.06.038.

18. Lv, L.; Shen, Y.; Liu, J.; Wang, F.; Li, M.; Li, M.; Guo, A.; Wang, Y.; Zhou, D.; Guo, S. Enhancing curcumin anticancer efficacy through di-block copolymer micelle encapsulation. *J. Biomed. Nanotechnol*. **2014**, *10*, 179–193, doi: 10.1166/jbn.2014.1809.
19. Cui, T.; Zhang, S.; Sun, H. Co-delivery of doxorubicin and pH-sensitive curcumin prodrug by transferrin-targeted nanoparticles for breast cancer treatment. *Oncol. Rep*. **2017**, *37*, 1253–1260, doi:10.3892/or.2017.5345.
20. Liu, G.; Khanna, V.; Kirtane, A.; Grill, A.; Panyam, J. Chemopreventive efficacy of oral curcumin: a prodrug hypothesis. *FASEB J*. **2019**, *33*, 9453-9465, doi:10.1096/fj.201900166R.
21. Ávila-Gálvez, M.Á.; Romo-Vaquero, M.; González-Sarrías, A.; Espín, J.C. Kinetic disposition of dietary polyphenols and methylxanthines in the rat mammary tissue. *J. Funct. Foods* **2019**, *61*, 103516, doi:10.1016/j.jff.2019.103516.
