# Supplementary material for: Dietary Phenolics against Breast Cancer. A Critical Evidence-Based Review and Future Perspectives
Source: Int J Mol Sci. 2020 Aug 10;21(16):5718. doi: 10.3390/ijms21165718 (PMC7461055; doi:10.3390/ijms21165718)
Supplement: Supplementary file 1 [file ijms-21-05718-s001.zip › ijms-890202-sup-proofed/Table S2.pdf]

Table S2. Animal studies conducted with dietary phenolics and(or) phenolic-rich extracts on BC models.

Animal Model (female)	Extract/Compound assayed	Dose/Duration	Main Outcomes	References
		**Anthocyanins**		
Sprague-Dawley rats; DMBA-induced BC	Concord grape juice concentrate	Diluted in drinking water 1:2, 1:1, and 2:1 (phenolic level 346, 519, and 642 mg/dL, respectively); 19 weeks.	↓Tumour volume and multiplicity (only at 519 mg/dL); ↓tumour weight (at 346 and 519 mg/dL); ↓DMBA-DNA adducts formation; ↑hepatic GST and catalase; ↓XO.	[1]
Wistar-Furth rats; MT-450 xenograft	Delphinidin	Exp. 1: 1.18 × 10^{-5} mol (s.c. injection); 21 days<br>Exp. 2: 1.18 × 10^{-5} mol (p.o.); 28 days.	Results in Exp. 1: ↑tumour growth, lymph node weight and lung metastasis; ↓CD31 and LYVE-1.<br>Results in Exp. 2: ↑tumour growth, lymph node weight and lung metastasis.	[2]
Sprague-Dawley rats; NMU-induced BC	Delphinidin	100 mg/kg/day (diet); 18 weeks.	↓Tumour incidence; ↓Ki-67 (in pulmonary metastatic tumour tissue and BC tissue). ↓*HOTAIR* expression and ↑miR-34a (in normal and BC tissue).	[3]
Immunodeficient BALB/c nude mice; MDA-MB-453 xenograft	Anthocyanin-rich extract from black rice (in the presence or absence of VEGF)	100 mg/kg/day (p.o.); 28 days.	↓Tumour growth, proliferation and microvascular density; ↓MMP-2, MMP-9, and urokinase-type plasminogen activator protein level.	[4]
BALB/c nude (*nu*/*nu*) mice; MDA-MB-453 xenograft	Anthocyanin-rich extract from black rice	150 mg/kg/day (p.o.); 21 days.	↓Tumour growth, pulmonary metastasis, lung tumour nodes and Ki-67.	[5]
BALB/c nude mice; MDA-MB-231-Luc-GFP xenograft	Delphinidin-3-glycoside	40mg/kg/day (p.o.); 28 days.	↓Tumour growth, *HOTAIR* expression and p-Akt protein level; ↑IRF-1 protein level.	[6]
Crl:NU-*Foxn1*^{nu}; MDA-MB-231 xenograft	Cyanidin-3-glycoside	1.6 and 3.2 g/kg (p.o., daily two times); 25 days.	↓Tumour growth (dose-dependent effect).	[7]
		**Flavanones**		

Sprague-Dawley rats; DMBA-induced BC	Orange juice<br>Grapefruit juice<br>Naringin<br>Naringenin	Orange and grapefruit juices were administered *ad libitum*, whereas naringin and naringenin were administered at 500 and 240 mg/100 gr diet, respectively (diets containing 5% and 20% corn oil); 15 weeks.	↓Tumour weight and burden; ↑tumour latency (only naringin and orange juice containing 5% corn oil). ↑Tumour weight (only grapefruit juice containing 20% corn oil). ↓Tumour burden (only naringenin containing 20% corn oil).	[8]
Sprague-Dawley rats; DMBA-induced BC	Hesperidin	30 mg/kg b.w.; 45 days.	Attenuation of the DMBA-induced deregulation on carbohydrate metabolizing enzymes[1], lipid profile[2] (plasma and liver) and ATPases[3] (erythrocytes and liver).	[9]
Wistar rats; DMBA-induced BC	Naringenin-tamoxifen (TAM) and Naringenin-TAM-Self-nanoemulsifying drug delivery systems (SNEEDS)	20 mg/kg; 30 days (60 days for survival assay).	The main effects were exerted by Naringenin-TAM-SNEEDS: ↓tumour growth and burden; ↑survival rate.	[10]
Sprague-Dawley rats; DMBA-induced BC	NSD containing [4]Heperetin-Mannitol (MAN)-sodium lauryl sulfate (SLS)-dioctyl sodium sulphosuccinate-DOSS.<br>PM containing [4]Heperetin-MAN-SLS-DOSS.<br>PM containing MAN-SLS-DOSS (placebo).	20 mg/kg orally (with respect to hesperetin) for each assay; once daily for 106 days.	Effects of NSD and PM treatments: ↑tumour latency; ↓tumour volume and weight; hepatotoxicity attenuation.	[11]
Wistar rats; DMBA-induced BC	Naringenin	2.5, 5, and 10 mg/kg b.w.; 19 weeks.	↓Tumour incidence, growth and weight; ↓proliferation; ↑apoptosis, caspase-3 and -9; ↑Phase I and II enzymes and ATPases[3] activity. Hepatotoxicity attenuation; modulation carbohydrate metabolizing enzymes[1], antioxidant status[5], mitochondrial mediated-apoptosis markers[6], lipid profile[2], and glycoprotein levels.	[12]

BALB/c mice (4T1 xenograft) and C57BL/6 mice	Naringenin	100 mg/kg/day (p.o.); up to 35 days (160 days for survival assay).	↑Survival rate; ↓lung metastases; ↑T cells activation and INF-γ and IL-2 expressing T cells.	[13]
BALB/c athymic mice (ovariectomized); MCF-7aro[7] xenograft (and androstenedione)	Hesperetin	500, 1000 and 5000 ppm; up 13 weeks.	↓Tumour volume and weight; ↓17 β-oestradiol level in plasma; ↓*pS2* and *CYP1A1* mRNA expression; ↓cyclin D1, CDK4, and Bcl-xL protein level; ↑p57^{Kip2} protein level.	[14]
BALB/c athymic mice (ovariectomized); [8]MCF-7aro xenograft (and androstenedione)	Hesperetin (alone or together with letrozole)	5000 ppm; 84 days.	↓Tumour volume and weight; ↓17 β-oestradiol level in plasma. Prevention of letrozole side-effects regarding bone deterioration.	[15]
BALB/c athymic mice; 4T1/RFP or 4T1/TGF-β1	Naringenin	200 mg/kg; 30 days.	Amelioration of the tumour-mediated immuno-supressive environment; ↓pulmonary metastasis; ↓TGF-β1 level in serum and lungs; ↑survival rate.	[16]
Obese C57BL/6J mice (ovariectomized); E0771 xenograft	Naringenin	1 and 3% (w/w) diet; 33 days.	↓Tumour growth; ↑p-Akt/Akt (T308 and S473) and p-mTOR/m-TOR (S2448) (mainly at 3% in mammary tumor). ↓adipose mass, adipocyte size and *α-SMA*, *MCP-1*, *IL-6*, and *leptin* mRNA expression (in mammary adipose tissue, mainly at 3%).	[17]
Athymic nude mice (*nu/nu*); MCF-7 or SKBR3 xenograft	2-Hydroxyflavanone	25 mg/kg b.w.; p.o. every other day for 8 weeks.	↓Tumour weight; ↓RLIP76, Ki-67, CD31, p-Akt, surviving, and vimentin; ↓Bcl-2/Bax ratio; ↑E-cadherin	[18]
NOD scid gamma mice; TMD231-luc cells	2-Hydroxyflavanone (alone or together with RLIP antibody + RLIP antisense)	50 mg/kg b.w.; p.o. every other day for 51 days.	↓Tumour growth and lung metastasis; ↓RLIP, p-Akt (Ser473) and p-ERK (T202/204); ↓Bcl-2/Bax ratio; ↓Ki-67, CD31, vimentin, CDK4; ↑E-cadherin.	[19]
		**Flavones**		

Sprague-Dawley rats; BC induced by DMBA and MPA medroxyprogesterone acetate	Apigenin	0.02, 0.1, and 0.5% (w/w) diet; 56 days.	↓Tumour incidence (only at 0.1%); ↑tumour multiplicity (at 0.1 and 0.5%).	[20]
BALB/c nude mice; MDA-MB-231 xenograft	Apigenin	25 and 50 mg/kg (in drinking water); 2 weeks.	↓Tumour growth; ↓pSTAT3/STAT3, pERK/ERK, IL-6, PI3K and p-Akt, N-cadherin.	[21]
Wistar rats; DMBA-induced BC	Luteolin (alone or together with cyclophosphamide)	30 mg/kg (p.o.); 20 days.	↓Tumour incidence and volume; ↓lipid peroxide formation; ↑SOD, catalase, and GPx level and activity.	[22]
BALB/c and athymic nude mice; 4T1 and MCF-7 xenograft, respectivley	Luteolin (alone or together with DOX)	100 mg/kg (p.o.); 3 weeks.	↓Tumour growth; ↑survival rate; ↓MDA in serum and ↑MDA in tumour (after luteolin + DOX treatment). ↑SOD, catalase; ↓MDA in plasma and ↑MDA in tumour (after luteolin treatment).	[23]
Athymic nude mice; MDA-MB-231 xenograft	Luteolin	0.01 and 0.05% (w/w) diet; 13 weeks.	↓Tumour growth and proliferating cells.	[24]
Athymic mice (ovariectomized); MCF-7aro[7] xenograft (and androstenedione)	Luteolin (alone or together with letrozole)	5, 20, and 50 mg/kg (b.w.); 84 days.	↓Tumour growth; ↓17β-oestradiol (at 50 mg/kg and together with letrozole); ↓uterine weight; ↑Bax/Bcl-xL; ↑HDL level (at 50 mg/kg); ↓cyclin A, D1, E, CDK4, CDK2, and p57.	[25]
BALB/c nude mice; MDA-MB-231 xenograft	Baicalein	50 and 100 mg/kg (p.o.); 15 days.	↓Lung and liver metastases; ↓SATB1, Wnt1, vimentin, β-catenin, SNAIL; ↑E-cadherin.	[26]
BALB/c nude mice; MCF-7 or MDA-MB-231 xenograft	Baicalein	100 mg/kg (p.o.); 21 days.	↓Tumour growth; ↓p-Akt; ↑Bax and LC3 protein level.	[27]
BALB/c nude mice; T47D and MDA-MB-231 xenograft	Wogonin	0.1, 1, and 10 mg/kg (p.o. 6 days per week); 4 weeks.	↓Tumour growth.	[28]
BALB/c nude mice; MDA-MB-231 xenograft	Wogonoside	80 mg/kg (p.o. once every other day); 21 days	↓Tumour growth and metastases; ↓MMP-9, vimentin, and twist1; ↑E-cadherin; ↓TNF-γ, TRAF2, and TRAF4.	[29]

BALB/c nude mice; MDA-MB-231 xenograft	Wogonoside	80 mg/kg (p.o. once every other day); 10 – 15 days.	↓Tumour growth and angiogenesis; ↓CD31 expression; ↓hemoglobin.	[30]
		**Flavonols**		
Sprague-Dawley rats; DMBA-induced BC	Quercetin (Quer)	1% (w/w) diet; 51 weeks.	No effects.	[31]
Sprague-Dawley rats; DMBA- and NMU-induced BC	Quer	2% and 5% (w/w) diet; 20 weeks.	↓Tumour multiplicity, latency and incidence.	[32]
Sprague-Dawley rats; DMBA-induced BC	Quer (alone or together with diallyl sulfide)	10 g/kg diet; 24 weeks.	↓Cumulative number of tumours (only significant in the presence of diallyl sulfide).	[33]
Sprague-Dawley rats; DMBA-induced BC	Quer	10 or 20 g/kg diet; 107 days.	↑Tumour latency (only at 20 g/kg).	[34]
ACI rats; 17β-oestradiol-induced tumour	Quer	2.5 g/kg; 8 months.	No protection against 17β-oestradiol-induced tumour; ↓tumour latency; ↓COMT activity.	[35]
Wistar rats; DMBA-induced BC	Quer	50, 100, and 200 mg/kg b.w. (p.o.); 16 weeks.	↑SOD, GPx, and catalase activity in erythrocyte lysates, plasma and breast tissue; ↓TBARS in plasma and ↑ in breast tissue.	[36]
Sprague-Dawley rats; DMBA-induced BC	TAM + Quer and TAM-Quer-NPs	TAM + Quer 3 mg/kg + 6 mg/kg (1:2 w/w), TAM-Quer-NPs equivalent to 3 mg/kg TAM (p.o. in a repeated dose of once in 3 days); 30 days (up to 60 days for survival study).	↓Tumour growth; ↑survival rate (after TAM-Quer treatment). ↑Quer plasma level; ↓tumour growth; ↑survival rate; ↓MMP-2 and MMP-9 plasma level; ↓hepatotoxicity (after TAM-Quer-NPs treatment).	[37]
Sprague-Dawley rats; DMBA-induced BC	Quer and Quer-SNEDDS	50 and 100 mg/kg; 12 weeks.	↑Tumour latency; ↓MMP-2 and MMP-9 plasma level; ↓tumour burden; ↓hepatotoxicity (Quer-SNEDDS treatment was the most effective).	[38]
Sprague-Dawley rats; DMBA-induced BC	TAM + Quer and TAM-Quer-SNEDDS	TAM + Quer 3 mg/kg + 6 mg/kg (1:2 w/w), TAM-Quer-SNEDDS equivalent to 3 mg/kg TAM (p.o. in a repeated dose of once in 3 days); 30 days (up to 60 days for survival study).	↑Survival rate; ↓tumour growth and burden; ↓MMP-2 and MMP-9 plasma level; ↓hepatotoxicity (TAM-quer-	[39]

			SNEDDS treatment was the most effective).	
Wistar rats; DMBA-induced BC	Quer	100 mg/kg b.w.; 30 days.	Induction of tumour regression; ↓tumour volume; ↓cellular infiltration in breast tissue; Preservation of normal breast tissue architecture; ↑SOD, catalase, and GPx activity; ↓AFP, CA 15-3, and CEA; ↓Hsp70 protein level.	[40]
Sprague-Dawley rats; DMBA-induced BC	Quer-RSV-GEN and Quer-RSV-GEN SNEDDS	Quer-RSV-GEN (equivalent to 50 mg/kg/week of Quer) and Quer-RSV-GEN SNEDDS (equivalent to 50 mg/kg/week of Quer); 91 days.	↑Survival rate; ↓tumour incidence and growth; ↓MMP-2, MMP-9, IL-6 and TNF-α plasma level (Quer-RSV-GEN SNEDDS treatment was the most active).	[41]
Sprague-Dawley rats; NMU-induced BC	Rutin (with DOX)	50 mg/kg (p.o. 7 days per week for 50 days).	↓Tumour growth in the presence of DOX; ↓side-effects exerted by DOX regarding cognitive function, inflammation, oxidative stress, as well as blood and immune cells.	[42]
Athymic *nu/nu* mice; GFP-MDA-MB-231 xenograft	Quer + RSV + Cath	0.5, 5, and 25 mg/kg (b.w.) each (p.o., 3 times per week); 117 days.	↓Tumour growth (at 5 and 25 g/kg).	[43]
BALB\c and athymic nude mice; 4T1 xenograft	Quer (alone or together with DOX)	100 mg/kg; 4 – 6 weeks (up to 100 days for survival study).	The effects were mainly observed in BALB\c mice: ↓tumour growth and lung metastases; ↑survival rate; ↑INF-γ and IL-2 level; ↓IL-4 and IL-10 level; ↓apoptosis of CD4+ and CD8+ T cells; ↑CD8+ T cells cytotoxicity against 4T1 cells.	[44]
Pathogen-free BALB\c mice; 4T1 xenograft	Quer (alone or together with DOX)	100 mg/kg (p.o.); 3 weeks.	↓Tumour growth, lung metastases and metastastic tumour weight; ↑ survival rate. Abrogation of the DOX-induced toxicity and modulation of HIF-1α and VEGF expression (only Quer treatment).	[45]

BALB/c and SCDI mice; JIMT-1 xenograft	Quer, vincristine, vincristine + Quer (molar ratio 2:1) and co-encapsulated liposomal vincristine[8]+ Quer (molar ratio 2:1)	Quer (0.24 mg/kg), vincristine (1.33 mg/kg), vincristine + Quer (0.24 mg/kg + 1.33 mg/kg), and co-encapsulated liposomal vincristine + Quer (0.24 mg/kg + 1.33 mg/kg); 60 days.	↑Circulation time and release *in vivo;* ↓tumour regression; ↑tumour latency and survival rate (co-encapsulated liposomal vincristine + Quer was the most effective treatment).	[46]
C3(1)/SV40 Tag	Quer	0.02, 0.2, and 2% (w/w) diet; 16 weeks.	Dissimilar effects on tumour number: ↑ at 2%, whereas ↓ at 0.2%; ↓tumour volume (dose-dependent); modulation of genes expression related to BC.	[47]
Swiss albino mice; Ehrlich ascites carcinoma cells injection	Quer	1 mg/kg; 50 days (up to 250 days for survival assay).	↓Tumour growth and Ki-67 level; ↑survival rate; ↑p53 and p-p53 protein level; ↑apoptosis.	[48]
BALB/c nude mice; MCF-7 xenograft	Quer	34 mg/kg; 21 days.	↓Tumour volume and weight; ↑tumour necrosis; ↓number of oncocytes; ↓microvessel density in tumour; ↓Ki-67 and vWF level; ↓VEGF, VEGF-R2, and NFATc3 (protein and mRNA); ↓calcineurin activity.	[49]
BALB/c nude mice; MCF-7 xenograft	Quer and vanadium-Quer complex	Quer (100 mg/kg) and vanadium-Quer complex (20 and 45 mg/kg); 24 weeks.	Preservation of normal breast tissue architecture; ↓cell proliferation; ↑p53; ↑Bax/Bcl2 ratio; ↑apoptosis.	[50]
Wistar rats; DMBA-induced BC	Myricetin	50, 100, and 200 mg/kg; 120 days.	↓TBARS (plasma and breast tissue); ↑SOD (erythrocyte lysate and breast tissue).	[51]
		**Isoflavones**		
Sprague-Dawley rats; DMBA-induced BC	GEN	25 or 250 mg/kg diet. GD 0 (*In utero*) to PND 21 (weaning).	↓Number of tumours; ↓number of TEB and TD; ↓number of lobules type I.	[52]
Sprague-Dawley rats; DMBA-induced BC	SPI	30, 40, and 810 mg/kg diet; from PND 36 to the end of the study (PND 127).	No significant chemopreventive effects.	[53]
F-344 rats; NMU-induced BC	SP and IDSP	10 and 20% for each treatment (w/w) diet; 19 weeks.	No significant chemopreventive effects.	[54]
Sprague-Dawley rats; DMBA-induced BC	SPI	Total isoflavones: 430 mg/kg diet (276 mg GEN/kg and 132 mg DAZ/kg diet); lifetime exposure (from GD 0 and for 2 generations).	↓Tumour incidence (%).	[55]

Sprague-Dawley rats; DMBA-induced BC	SOYSELECT™ extract (12% isoflavones)	0.35 or 0.7% extract (w/w); from weaning (PND 21) to the end of the study (PND 218).	↑Degree of tumour differentiation; ↓percentage of ERα and PR positive tumour.	[56]
Sprague-Dawley rats; DMBA-induced BC	GEN, DAZ, GEN + DAZ, SPI and SPId	GEN or DAZ (200 mg/kg diet), GEN + DAZ (100 + 100 mg/kg diet), SPI or SPId (160 g/kg diet); from PND 43 to PND 170.	↓Tumour multiplicity (only DAZ, SPI, and SPId).	[57]
Sprague-Dawley rats; DMBA-induced BC	SPI	430 mg total isoflavones/kg diet (including 276 mg/kg GEN and 132 mg/kg DAZ equivalents); from GD 4 to PND 48.	↓Hepatic EROD and MROD; ↓mammary CYP1A1, CYP1A2, CYP1B1 (mRNA and protein); ↓AhR and ARNT protein level (although the mRNA was increased).	[58]
Sprague-Dawley rats; DMBA-induced BC	SPI	20% (w/w) diet; from GD 4 (in utero) to the end of the study (PND 21, 33, 50 or between PND 48-51 on metaestrous).	↓TEB density (not significant); ↑PgR positive cells in TEB.	[59]
Sprague-Dawley rats; DMBA-induced BC	GEN	15, 150, and 300 mg/kg diet; from GD 0 (in utero) to PND 0 (birth).	↓17β-oestradiol; ↑TEB; ↓lobule (in the offspring at 8 weeks).	[60]
Sprague-Dawley rats; DMBA-induced BC	DAZ	250 mg/kg diet; from PND 0 (birth) to PND 21 (weaning).	No significant chemopreventive effects.	[61]
Sprague-Dawley rats (ovariectomized); DMBA-induced BC	GEN	25 and 250 ppm; 36 weeks.	↑mean tumour volume (at 25 ppm).	[62]
Wistar rats (ovariectomized);	Exp. 1: Soylife, rutin and flaxseed<br>Exp. 2: Novasoy and Soylife<br>Exp. 3: Novasoy and α-oestradiol	Exp. 1: Soylife (0.5%), rutin (0.25%), and flaxseed (10%).<br>Exp. 2: Novasoy (20, 40, and 80 µg/g b.w.) and Soylife (4, 8, 16 µg/g b.w.).<br>Exp. 3: Novasoy (20, 40, and 80 µg/g b.w.) and α-oestradiol (30 µg/g b.w.).<br>90 days.	Exp. 1: ↑*BRCA-1* mRNA expression (only flaxseed).<br>Exp. 2: ↑*BRCA-2* mRNA expression (only at the highest doses). Exp. 3: ↑*BRCA-1* and *BRCA-1* mRNA expression (only at the highest doses).	[63]
Sprague-Dawley rats	GEN and SPI+	GEN (250 mg/kg diet), SPI+ total isoflavones: 394 mg/kg diet (216 and 160 mg/kg diet GEN and DAZ, respectively); from GD 4 (in utero) to PND 50.	↑Apoptosis in mammary glands; ↑PTEN; ↑*p21*, *Bax*, and *Bok* gene expression (only GEN).	[64]
Sprague-Dawley rats; DMBA-induced BC	DAZ and GEN	DAZ (140 mg/kg) and GEN (105 mg/kg (w/w) diet) (both compounds were investigated in the presence or absence of TAM); 114 days.	↓Tumour multiplicity and incidence; ↑latency; ↓8-OHdG level.	[65]
Sprague-Dawley rats; NMU-induced BC	SPI+ diet	Food and water provided *ad libitum* through the study; from GD4 (in utero) to the end of the study (PND165).	↓Tumour incidence; ↑latency; ↑*HRE2/neu* and *PR* gene expression; ↓progesterone in plasma.	[66]

Sprague-Dawley rats; DMBA-induced BC	SP	20% (w/w) diet; 172 days.	↓Tumours number and incidence; absence of aggressive grade II and III tumours; ↑tumour latency; ↓angiogenesis; ↓VEGF and bFGF level; ↓proliferation.	[67]
Big Blue ® (BB) rats (ovariectomized and normal); DMBA-induced BC	GEN, DAZ and GEN + DAZ	GEN or DAZ (0.25 or 1 mg/kg diet) or GEN + DAZ combined (1 mg/kg diet of each); 23 and 27 weeks.	↓DMBA-induced mutagenesis (*lac*I and *Hprt*); ↑cell proliferation.	[68]
Sprague-Dawley rats; NMU-induced BC	GEN and SPI	GEN (250 mg/kg diet) and SPI diet containing 394 mg isoflavones/kg diet (216 and 160 mg/kg GEN and DAZ, respectively); from GD4 to birth (PND 0) (in utero), and lifetime from GD4 to the final of the study (PND 149).	↑Tumour latency; ↓tumour multiplicity; ↓higher grade tumour; ↑E-cadherin expression (in utero after SPI treatment). ↑E-cadherin expression (in utero after GEN treatment). ↓Tumour incidence; ↑apoptosis in TEB; ↑PTEN expression; ↑p53 phosphorylation (lifetime after SPI treatment). No effects (lifetime after GEN treatment).	[69]
Sprague-Dawley rats; DMBA-induced BC	Soy milk	0.28 mg/mL isoflavones; 20 weeks.	↑Mammary tumour incidence and tumour multiplicity.	[70]
Sprague-Dawley rats;	Soya and soya + fish oil	Soya (0.5% (w/w) diet (705 mg/kg isoflavonoids)) or soya + fish oil (80 g/kg + 0.5% (w/w)) diet; 14 – 15 days.	↑TEB; ↑ER-*α* and ↓ER-β (soya diet). ↑Apoptosis (soya + fish diet).	[71]
Sprague-Dawley rats (ovariectomized); DMBA-induced BC	GEN, DAZ and SPE	GEN or DAZ (50 mg/kg) diets, and SPE (50 and 100 mg/kg) diet; 20 weeks.	↓Tumour weight (only after SPE diet); ↓angiogenesis; ↑apoptosis; ↓VEGF level; ↑endostatin level; distinct effect on proliferation: ↑ by GEN and ↓ by SPE.	[72]
Sprague-Dawley rats; DMBA-induced BC	IDSP	3.3 gr/rat/day (dietary administration); 18 weeks (4 weeks before and 14 weeks after DMBA administration).	↓Tumour growth and incidence; ↑latency; ↓HSP90 and NF-ƙB; ↑p53, p21, and caspase-3; ↑caspase-3 activity; ↓VEGF level.	[73]
Wistar rats; DMBA-induced BC	GEN and GEN + lycopene	GEN (2 mg/kg b.w.) and GEN + lycopene (2 + 20 mg/kg b.w.) (p.o. 3 times per week); 20 weeks.	↓Tumour incidence, growth and development; ↓MDA, 8-isoprostane and 8-OHdG.	[74]

Zucker rats (lean and obese); DMBA-induced BC	SPI	Isoflavones: 3.24 mg/g protein; 22 weeks.	↑Mammary tumour development (in obese rats); modulation of IGFBP-3 and IGF-1 levels	[75]
WKA rats; Ethyl methanesulphonate-induced mammary cancer	GEN and soy diet	GEN (0.03 and 1 mg/g) diet and soy diet (composition not provided); 12 weeks (up to 24 weeks for survival study).	No protective effect of GEN. ↑Latency periods after soy-enriched diet but compared to GEN-treated groups.	[76]
Sprague-Dawley rats; PhIP-induced BC	Soymilk (alone or together with 2 × 10^{11} CFU/kg *Lactobacillus casei* Shirota)	100 g/kg diet (total isoflavones: 335 mg/kg diet).	Effects of soymilk: ↓tumour incidence and multiplicity; ↓body weight (after soymilk treatment).<br>↓Tumour multiplicity and diameter; ↓body weight and liver relative weight; ↓ERα-positive and Ki-67-positive cells; ↓microvessel density (after soymilk + *L. casei*).	[77]
Ovariectomized athymic nude mice; MCF-7 xenografts	GEN	750 ppm; 5 days and 12 weeks.	↑Tumour growth; ↑uterine and mammary growth; ↑TEB.	[78]
Athymic nude mice (ovariectomized); MCF-7 xenografts	GEN and genistin	GEN (750 ppm) and genistin (1200 ppm); from PND 56 to the end of the study (PND 133).	↑Tumour growth and cell proliferation on MCF-7 tumour; ↑*pS2* gene expression.	[79]
Athymic nude mice (ovariectomized); MCF-7 xenografts	SPI and GEN	SPI and GEN diets contain 15, 150, and 300 ppm GEN; 29 weeks.	Dose-dependent effects: ↑tumour growth and cell proliferation on MCF-7 tumour; ↑*pS2* gene expression.	[80]
Wild-type (ERαWT) and ER-α knockout (ERαKO) mice; DMBA-induced BC	GEN	1 g/kg diet; ~34 weeks.	↑Malignant tumours (only in ERαWT)	[81]
Athymic BALB\c mice (ovariectomized); MCF-7 xenografts	GEN	0.125 – 1 mg/g diet; 22 weeks.	↑Tumour growth (dose-dependent) and cell proliferation; ↑*pS2* gene expression.	[82]
Athymic BALB\c mice (ovariectomized); MCF-7 xenografts	GEN (in the presence of TAM)	1000 ppm; 32 weeks.	GEN abrogated the effect of TAM: ↑tumour growth; ↑*pS2*, *PR receptor*, and *Cyclin D1* gene expression; ↓17β-oestradiol plasma level.	[83]

MMTV-neu transgenic mice	GEN, DAZ and isoflavone mixture (NovaSoy)	GEN or DAZ (250 mg/kg) diets, and NovaSoy contains 250 mg GEN equivalent/kg diet; from PND 42 to the final of the study (up to 37 weeks).	↑Tumour latency.	[84]
FVB/N-TgN (MMTV-*neu*) mice	Soy diet (Purina 5001)	Isoflavones content: 491 mg/kg (227 mg/kg DAZ and 214 mg/kg GEN); 15 – 60 weeks.	↑Tumour latency; ↑differentiation of the distal end buds.	[85]
Ovariectomized athymic nude mice; MCF-7 xenograft	Soy flour + mixed isoflavones, Molasses, Novasoy®, mixed isoflavones, and genistin	All the diets contained same amount of aglycones GEN equivalents 750 ppm; 11 weeks.	↑Tumour growth; ↑cell proliferation; ↑*pS2* and *cyclin D1* mRNA expression (after NovaSoy, mixed isoflavones, and genistin treatments). ↑*PgR* mRNA expression (only genistin treatment).	[86]
SCDI mice; MCF-7 xenograft	SPC, GSI, BT, GT, SPC + BT, and SPC + GT	SPC (0.1 and 0.5%), GSI (0.014 and 0.028), BT (1.5% (w/v)); GT (1.5% (w/v)); SPC + BT and SPC + GT (0.1% + 1.5% (w/v)); 10 weeks.	↓Tumour growth (except BT); ↓cell proliferation; ↑apoptosis (BT, GT, and mixtures); ↓vessel density (mixtures); ↓ER-α (BT and mixtures); ↓IGF-I (only SPC + GT).	[87]
FVB-Tg.NK (MMTV/c-*neu*) mice (wt-erbB2)	Soy meal, high-dose isolated soy and low-dose isolated-soy	Soy meal contains 491 μg/g isoflavones (214 and 277 μg/g GEN and DAZ, respectively), the high-dose isolated soy contains 491 μg/g isoflavones (214 and 277 μg/g GEN and DAZ, respectively), and the low-dose isolated-soy contains 211 μg/g isoflavones (137 and 74 μg/g GEN and DAZ, respectively); 60 weeks.	↓Tumour incidence (only soy meal). Low-dose diet blocked the TAM-preventive effects.	[88]
FVB-Tg.NK (MMTV/c-*neu*) mice	Isoflavone-concentrate Prevastein	High-fat (20% w/w) diet supplemented with 0.004, 0.02 and 0.06% (w/w) Prevastein; 24 weeks.	↑Tumour size and multiplicity (at the highest dose).	[89]
BALB\c (nude) mice (ovariectomized); MCF-7 xenografts	GEN (in the presence or absence of 17β-oestradiol)	GEN: 500 ppm; 17 weeks.	↑Tumour growth (in the presence or absence of 17β-oestradiol). ↑Uterine wet weight (in the absence of 17β-oestradiol).	[90]
Athymic BALB\c mice (ovariectomized); MCF-7 xenografts	DAZ and equol	125 – 1000 ppm; 21 and 37 weeks.	↑MCF-7 tumour growth and *pS2* expression (only DAZ treatment).	[91]

SCID mice; MCF-7 xenografts	GEN and SPC (alone or together with TAM)	GEN (0.07% w/w diet), SPC (0.2 and 0.5% w/w diet); ~80 days.	Effects of GEN: ↓tumour growth; ↓*pS2* and *EGFR* gene expression; ↓proliferation; ↓17β-oestradiol; ↑apoptosis (only with TAM); ↓*ER-α* gene expression; ↓angiogenesis. Effects of SPC: ↓tumour growth; ↑apoptosis; ↓proliferation (alone at 0.5% and with TAM); ↓angiogenesis (only with TAM).	[92]
Tg.NK (MMTV/c-*neu*) mice	Isoflavone-concentrate Prevastein	90 mg/kg mixed in high-fat (24% w/w) diet prepared with corn or fish oil; from 2 weeks before mating to PND 21 (perinatal), from PND21 to the end of the study (postweaning), and from 2 weeks before mating to the end of the study (lifetime).	↓Tumour incidence and delayed tumour onset.	[93]
BALB/c nude mice (ovariectomized); [9]MCF-7Ca xenograft	GEN (in the presence or absence of androstenedione and letrozole)	250, 500, and 1000 ppm; 19 and 31 weeks.	In absence of androstenedione and letrozole: ↑tumour growth; ↑*pS2* gene expression (at 250 and 500 ppm); ↓*aromatase* gene expression (at 1000 ppm); ↑proliferation. In presence of androstenedione and letrozole: ↑tumour growth (dose-dependent); ↑pS2 (mRNA and protein); ↑17β-oestradiol; ↑aromatase protein level; ↑proliferation; ↑uterine wet weight (dose-dependent).	[94]
BRCA-1^{tm1Cxd} heterozygous (+/-) and wild type mice; DMBA-induced BC	GEN	500 ppm; from PND 15 – PND 30.	Effects on wild type mice: ↓tumour incidence; ↓high-grade tumours; ↑*BRCA1* mRNA expression; ↓CK5 and CK18; ↓TEB and ductal elongation; ↓PR in mammary gland and tumour; ↓ER-*α* and HER2 in mammary tumour;	[95]

			↓*amphiregulin* and *RankI* gene expression. Effects on BRCA-1$^{+/-}$ mice: ↓CK5 and CK18; ↓TEB and ductal elongation; ↓HER2 in mammary tumour; ↓*amphiregulin* gene expression.	
Transgenic MMTV-erbB2 mice	Soybean-enriched diet (20% soybean)	Total isoflavones: 2.6 mg/g diet; from 7 weeks of age to the final of the study (up to 60 weeks).	↓Breast tumour incidence at high oestrogen level, but ↑breast tumour incidence at low oestrogen level.	[96]
MMTV-*Neu* and ERΔ3/*Neu*	Soy diet	Total isoflavones: 619 mg/kg diet (GEN: 191 mg/kg diet, DAZ: 143 mg/kg diet, GLY: 39 mg/kg diet); from 2 months of age to the final of the study.	No effect of soy diet on tumour development; ↑metastatic lesions in ERΔ3/*Neu* mice.	[97]
Nu/Nu Nude mice Crl:NU-*Foxn1nu* (MDA-MB-231 xenograft) and C3(1)-SV40 Tag mice	GEN (in the presence or absence of TAM)	250 mg/kg diet; 5–6 weeks.	Effects on Nu/Nu nude mice Crl:NU-*Foxn1nu*: ↓tumour growth (in absence or presence of TAM); ↑ER-α (gene and protein); ↓DNMT1 and HDAC1 (gene expression, protein level, and enzymatic activity). <br>Effects on C3(1)-SV40: ↓tumour growth (in absence or presence of TAM); ↑latency tumour; ↑ER-α (gene and protein); ↓DNMT1 (gene expression and activity); ↓HDAC1 (gene expression, protein level, and activity).	[98]
Transgenic Wnt1-Tg mice	SPI	Total isoflavones: 430 mg/kg diet; fom PND 21 (weaning) to the final of the study.	Modulation of epithelial subpopulations in mammary tissue; ↓progesterone; ↓17β-oestradiol; ↓IL-6; modulation of expression of genes related to inflammation, cytokine signaling, and proliferation;	[99]

			↓mammospheres formation in culture; ↓tumour latency and incidence.	
MTB-IGFIR transgenic mice (doxycycline supplemented diet)	SPI	Isoflavones: 1660 ppm (daidzin: 463 ppm, DAZ: 95 ppm, genistin: 933 ppm, GEN: 101 ppm, glycetin: 57 ppm, GLY: 9 ppm); from GD 0 (*in utero*) to the final of the study (up to PND 365).	↑Tumour incidence and onset; ↑ER signaling markers (*Pgr* and *Areg*); ↑CK5 and CK14; ↑p63; ↓lung metastasis.	[100]
Germ-free RAG2$^{-/-}$ athymic mice transplanted with breast cancer patients' fecal suspensions obtained before and after chemotherapy treatment (DOX and docetaxel); MDA-MB-231 xenografts	GEN	250 mg/kg; 8 weeks.	↓Tumour growth; ↑tumour latency; modulation of gut microbiota at Phylum (i.e., Phylum *Verrucomicrobia*), family (i.e., *Lachnospiraceae* and *Ruminococcaceae*) and genera level (*Lactococcus* and *Eubacterium*).	[101]
C3(1)-SV40 Tag transgenic mice	GEN and GEN + broccoli sprouts (BSp)	GEN (250 mg/kg), GEN + BSp (250 mg/kg + 13% (w/w)) diets; from 4 weeks of age to the final of the study.	↑Tumour latency.	[102]
BALB/c mice; 4T1 xenograft	DAZ	145 mg/kg (p.o.); 20 days of exercise training followed by 22 days of orally administration of DAZ.	Effects of DAZ: ↓tumour growth; ↑Fas, ↑FADD, ↑Bax, ↑Cyt-C, ↑Apaf-1 ↑cleaved caspase-3 and 9, and ↓Bcl-xL. Effects of DAZ + exercise: ↓tumour growth; ↑IL-6 and epinephrine; ↑infiltration of NK cells; ↑apoptosis; ↑Fas, ↑FADD, ↑Bax, ↑Cyt-C, ↑Apaf-1, ↑cleaved caspase-3 and 9, and ↓Bcl-xL.	[103]
		**Flavan-3-ols/Proanthocyanidins**		
Sprague-Dawley rats; DMBA-induced BC	GT Cath	1% (w/w) diet; PND 56 – PND 294.	↑Survival rate; ↓tumour size.	[104]
F344 rats; PhIP-induced BC	GT Cath	1% (w/w) diet; up to 52 weeks.	↓Tumour size	[105]
Sprague-Dawley rats; DMBA-induced BC	GT Cath	0.01, 0.1, or 1% (w/w) diet; 34 weeks.	↓tumour multiplicity at 0.01 and 1% (lack of dose-dependent effect).	[106]
Sprague-Dawley rats; DMBA-induced BC	Black tea	1.25, 2, and 2.5% (w/v) in water; up to 17 weeks.	↓Tumour burden and tumour weight per TBA (only 2%).	[107]
Sprague-Dawley rats; DMBA-induced BC	GT	0.3% (w/v) in water; PND 30 – PND 175.	↓Tumour burden; ↓invasive tumour; ↑latency.	[108]

Sprague-Dawley rats; dual organ tumour model: DMBA-induced BC and azoxymethane-induced colonic aberrant crypt foci formation	Grape seed proanthocyanidins	0.1, 0.5, and 1% (w/w) diet; 27 weeks.	No effect on mammary tumourigenesis.	[109]
F344 rats; PhIP-induced BC	GT Cath	1% (w/w) diet; 52 weeks.	↓Tumour size.	[110]
Sprague-Dawley rats; DMBA-induced BC	EGCG	0.065% (w/v) in drinking water; Lifetime: from birth (PND 0) to the end of the study (PND 176).	No effects on mammary tumourigenesis.	[111]
Fisher-344 and Sprague-Dawley rats; MNU-induced BC	Polyphenon E	333 and 1000 mg/kg b.w. (p.o. 7 times per week); 121 days of treatment.	No effects on mammary tumourigenesis.	[112]
BALB/c mice; 4T1 cells xenograft	GTP (60% EGCG)	0.2 and 0.5% (w/v) in drinking water; 36 – 60 days.	↓Tumour growth; ↓proliferation (PCNA); ↓lung metastasis; ↑survival time; ↑cleaved caspase 3 and Bax/Bcl-2.	[113]
C3(1) SV40 *T,t* antigen transgenic multiple mammary adenocarcinoma mice	GT Cath or BT theaflavins	0.01 or 0.05% (w/v) in water; from 4 weeks of age to the end of the study.	↑Survival rate; ↓tumour diameter; ↑cleaved caspase-3 level.	[114]
Athymic nude mice (NCr-nu/nu); MDA-MB-231 xenograft	GTP and EGCG	GTP (1% (w/v) in drinking water) and EGCG (1mg per animal (p.o. in 100 mL distilled water)); 70 days.	↓Tumour volume; ↓cell proliferation; ↑apoptosis.	[115]
C57BL\6 mice; E0771 xenograft	EGCG	0.5 g/L in drinking water; 24 days.	↓Tumour growth and angiogenesis; ↓VEGF level (plasma and tumour).	[116]
BALB/c mice; 4T1 cells xenograft	GT extract	0.6 g/kg (orally fed everyday); 4 weeks.	↓Tumour weight; ↓lung and liver metastasis; ↓breast cancer-induced bone destruction.	[117]
BALB/c mice; 4T1 cells xenograft	EGCG	0.25, 0.5, 1, and 2 mg/mL in drinking water; 51 days.	↓Tumour growth (dose-dependent); ↓accumulation of MDSCs in blood, spleen, and tumour; ↑CD4+ and CD8+ T cells.	[118]
Nude mice (ovariectomized); MCF-7 xenograft	GTE (alone or in combination with TAM)	2.5 g/L in water; 64 days.	↓ERα level; ↓tumour volume and angiogenesis; ↑necrosis and apoptosis (GTE + TAM was the most effective treatment).	[119]
Wistar rats; MNU-induced BC	GTE, Epican Forte, Epican Forte + nutrient mix, Epican Forte + Quer and Epican Forte + nutrient mix + Quer	GTE (30 mg), Epican Forte (30 mg), Epican Forte + nutrient mix (130 + 405 mg), Epican Forte + Quer (130 + 0.3 mg), and Epican Forte + nutrient mix + Quer (130 + 405 + 0.3 mg); 60 days.	↓Tumour volume, weight and multiplicity (Epican forte + nutrient mix + quercetin was the most effective treatment).	[120]

C3H/OuJ mice	GTE (alone or together with TAM)	0.1 or 1% (w/v) in water; PND 70 – PND 336 weeks.	↓Tumour incidence (no tumour development in green tea + TAM group); ↓proliferation.	[121]
		**Stilbenes**		
Sprague-Dawley rats; NMU-induced BC	RSV	10 and 100 mg/kg b.w. (p.o. 5 days/week); 127 days.	RSV at 100 mg/kg: ↓tumour incidence and multiplicity; ↑tumour latency; ↑alveolar and adipocyte differentiation.	[122]
Sprague-Dawley rats; DMBA-induced BC	RSV	100 μg/rat in the diet; 127 days.	↓Ductal carcinoma induction; ↓tumour incidence and multiplicity; ↑tumour latency; ↓NF-kB activation; ↓COX-2; ↓MMP-9 level.	[123]
Sprague-Dawley rats; DMBA-induced BC	RSV	1 g/kg diet; from PND 0 (birth) to the end of the study (PND ~180).	↓Number of tumours; ↑tumour latency; ↑differentiated lobular structures; ↓cell proliferation in terminal ductal structures; ↑apoptosis.	[111]
Sprague-Dawley rats; DMBA-induced BC	RSV	100 μg/rat in the diet; 24 weeks.	↓DNA damage; ↓4-hydroxynonenal level; ↓lipid peroxidation; ↑apoptosis; ↑caspase-3; ↓5-LOX expression; ↓LTB_4 formation; ↓NF-kB p65; ↑TGF-β1; ↓cell proliferation.	[124]
Sprague-Dawley rats; NMU-induced BC	RSV (alone or in combination with melatonin)	100 mg/kg diet; 18 weeks.	↓Tumour incidence; ↓invasive and *in situ* carcinomas.	[125]
ACI rats; subcutaneous administration of 17β-oestradiol	RSV	5 and 25 mg/kg/day; 21 weeks.	↓*DNMT 3b*; ↓*DNMT1* (only at 25 mg/kg/day); ↑miRNA-21, miRNA-129, miRNA-204, miRNA-489 (in breast tumour tissue).	[126]
Sprague-Dawley rats; NMU-induced BC	RSV (alone or in combination with celecoxib)	100 mg/kg b.w.; 16 weeks.	Effects of RSV + celecoxib: ↓tumour incidence; ↓tumour	[127]

			volume; ↓tumour frequency; ↓number of malignant tumours; ↑mammary tumour latency; ↑GDF15 protein expression; ↓*COX-2* gene expression; ↓ROS in lymphocytes.	
Sprague-Dawley rats; exposure to TCDD	RSV	7 ppm; from GD7 to PND 0 (birth).	↓TD + TEB; ↑ alveolar bud; ↑AhRR protein expression; ↑*BRCA-1* gene expression; ↓*BRCA-1* promoter CpG methylation; ↓*CDK4* gene expression; ↓*Cyclin D1* gene expression; Dissimilar effect regarding *CYP1A1* gene expression: down-regulation at PND 46, and up-regulation at PND 71.	[128]
Sprague-Dawley rats; DMBA-induced BC	RSV, RSV-CoenzQ10 SNEDDS, RSV-CoenzQ10-α-tocopherol SNEDDS	RSV (50 mg/kg), RSV-CoenzQ10 SNEDDS and RSV-CoenzQ10-α-tocopherol SNEDDS (equivalent to 50 mg/kg RSV); 91 days.	↓Tumour latency and growth; ↓MMP-2 and MMP-9; ↓IL-6 and TNF-α; ↑survival rate. RSV-CoenzQ10-α-tocopherol SNEDDS was the most effective treatment, what was related to the highest level of RSV and coenzQ10 achieved in plasma.	[129]
Wistar rats; NMU-induced BC	RSV	20 mg/kg b.w.; GD 10 – PND 21.	↓Number of tumours; ↓tumour weight; ↑apoptosis; modulation of genes related to apoptosis (i.e., *caspase-3* and *Bcl-2*); modulation of morphological changes in mammary tissue (such as ↓ TEB and TD).	[130]
Sprague-Dawley rats; NMU-induced BC	RSV	100 mg/kg b.w. (p.o.); 16 weeks.	↓Tumour volume, frequency and incidence; ↑latency; ↑number of leukocytes; ↑ROS in leukocytes.	[131]
Sprague-Dawley rats; DMBA-induced BC	RSV	50 mg/kg (p.o., every other day); 18 weeks.	Modulation of oestrogen level (serum and breast tissue).	[132]

FVB/N HER-2/*neu* mice	RSV	1 mg/L (diluted in water); 11 weeks.	↓Number and size mammary tumour; ↓lung metastases; ↑apoptosis; HER-2/*neu* down-regulation.	[133]
Athymic *nu/nu* mice; GFP-MDA-MB-231 xenografts	RQC	0.5, 5, and 20 mg/kg b.w. (3 times per week); 117 days.	↓Tumour area (at 5 and 20 mg/kg b.w.).	[43]
Athymic *nu/nu* mice; GFP-MDA-MB-435 xenografts	RQC	5 mg/kg b.w. (p.o. three times per week); 70 days.	↓Tumour area and metastasis; modulation of expression of PI3-kinase pathway genes.	[134]
Hairless SCDI mice; GFP-MDA-MB-231 xenograft	RQC (alone or together with gefitinib)	(i) 5 mg/kg b.w. each; 84 days.	↓Tumour growth and metastasis (RQC + gefitinib was the most effective treatment).	[135]
Hairless SCID mice (GFP-MDA-MB-231) or athymic nu/nu mice (MDA-MB-435 xenografts)	RSV	0.5, 5, 50 mg/kg b.w. (p.o. 5 days/week); 44 days (GFP-MDA-MB-435) or 108 days (GFP-MDA-MB-231).	↑Tumour growth; ↑lung metastasis (only significant at 5 and 50 mg/kg); ↑ Rac activation.	[136]
Δ16HER2 mice	RSV	1 mg/L in water (4 mg RSV/mouse was the daily dose assumed); 15 weeks.	↓tumour latency; ↑tumour multiplicity; ↑Δ16HER2 expression; ↓ER*α* expression; inhibition of proteasome activity.	[137]
BALB\c homozygous, Crl:NU(NCr)-Foxn1nu; MBCDF-T cells xenograft	RSV (alone or together with calcitriol)	1.2 g/kg (p.o. 3 times a week); 3 weeks.	↓Angiogenesis (RSV and calcitriol).	[138]
		**Curcumin**		
Sprague-Dawley rats; DMBA-induced BC	Curcumin	10 or 20 g/kg diet; 107 days.	↑Tumour latency (only at 20 g/kg).	[34]
Sprague Dawley rats; DMBA-induced BC	Curcumin, Dibenzoylmethane	*Mammary adduct assay:* 0.2 and 1% of both compounds; 10 days.<br>*Tumour inhibition:* curcumin (1% w/w diet) and dibenzoylmethane (0.1, 0.5, and 1% w/w diet); 14 days.	Only dibenzoylmethane: ↓tumour incidence (dose-dependent effect); ↓DMBA-DNA adducts formation (at 1%); ↑liver GST, quinone reductase, and EROD (at 1%).	[139]
Sprague Dawley rats; DMBA-induced BC	Turmeric, ethanolic turmeric extract,<br>curcumin-free aqueous turmeric extract	Turmeric (1% w/w diet), ethanolic turmeric extract (0.05% w/w diet), and curcumin-free aqueous extract (1% in water; w/v); 4 weeks (*initiation period)* and 20 weeks (p*ost-initiation period*).	Effects *initiation period*: ↓tumour incidence, burden and multiplicity (turmeric and ethanolic turmeric extract).<br>Effects *post-initiation period*: ↓tumour multiplicity (ethanolic turmeric extract and curcumin-free aqueous turmetic extract).	[140]

Wistar-MS rats; irradiation with γ-rays (gestational period) and subcutaneous implantation of diethylstilbestrol pellet	Curcumin	1% diet (w/w); 13 months.	↓Tumour incidence; ↓serum prolactin; ↓TBARS.	[141]
Wistar-MS rats; irradiation with γ-rays (gestational period) and subcutaneous implantation of diethylstilbestrol pellet	Curcumin	1% diet (w/w); GD 11 – PND 0 (birth).	↓Tumour incidence and latency; ↑LH level.	[142]
Athymic *nu/nu* mice; MCF-7 or BT-474 xenograft	Curcumin	25 gr/kg diet; 3 days.	↑Tumour growth (in mice treated with cyclophosphamide).	[143]
Athymic nude (*nu/nu*) mice; MDA-MB-435LVB xenograft	Curcumin (alone or together with PAX)	2% diet (w/w); 5 weeks.	↓BC metastases to the lungs; ↓NF-ƙB activation; ↓COX-2 and MMP-9 levels.	[144]
CD-1 Foxn1^{nu} mice; MDA-MB-231 xenograft	Curcumin	1% diet (w/w); 35 days.	↓Metastases.	[145]
Athymic nude mice; ENU1564 xenograft	Curcumin, meriva	Curcumin (2% w/w diet) and Meriva (6% w/w diet); 20 days.	Effects only in the Meriva-treated group: ↓breast cancer metastases to the lungs; ↓MMP-9 level.	[146]
Nude (Foxn1$^{nu/nu}$) mice; MBA-MB-231 xenograft	Curcumin	Curcumin (0.6% w/w diet); 6 weeks.	↓Tumour growth; ↓NF-kB; ↓PECAM-1; ↓cyclin D1; ↓p65.	[147]
CD1 athymic nude mice; MDA-MB-231 xenograft	Curcumin (alone or together with EGCG)	200 mg/kg/day; 10 weeks.	↓Tumour volume; ↓EGFR; ↓VEGFR-1; ↓Akt (synergic effects: curcumin and EGCG).	[148]
NCr-nu/nu mice; MDA-MB-231 xenograft	Curcumin (alone or together with PAX)	100 mg/kg; 5 weeks.	↓Tumour diameter; ↓PCNA-positive cells in tumour; ↑apoptosis; ↑MMP-9.	[149]
Swiss albino mice; Ehrlich ascites carcinoma cells xenograft	Curcumin (alone or together with DOX)	25 and 50 mg/kg b.w. (p.o., every oher day); 21 days.	↓Number of cancer cells in tumour; ↓DOX-induced toxicity; ↑survival rate.	[150]
Swiss albino mice; DMBA-induced BC	Curcumin (alone or together with cyclophosphamide or PAX)	40 mg/kg b.w. (p.o.); 4 weeks.	↓Tumour volume; ↓PKC; ↓NF-ƙB; ↓HDAC1 and 2 (curcumin enhanced effects of cyclophosphamide and PAX.	[151]
SENCAR (SENsitive to CARcinogen) mice; DMBA-induced BC	Curcumin (alone or together with DHA)	0.2% diet (w/w); 19 weeks.	↓Tumour incidence, burden and width (per gram body); ↑tumour differentiation and latency; ↑ER-α; ↑maspin protein level; ↓survivin protein level.	[152]

Athymic nu/nu mice; MDA-MB-231 xenograft	Curcumin (alone or together with ABT-888)	20 mg/kg b.w. (3 times per week); 7 weeks.	↓Tumour growth.	[153]
BALB\c mice; MCF-10A-Tr xenograft	Curcumin (alone or together with RSV)	20 mg/kg/day (each treatment); 14 days.	↓Cyclin D1; ↓p21; ↓Gli-1; ↓SHH (27 and 52 kDa); ↓c-Myc (synergic effects between curcumin and RSV).	[154]
BALB\c mice; EMT6/P mouse breast carcinoma cells xenograft	Curcumin (alone or together with metformin)	50 mg/kg (100 μL by oral gavage everyday); 14 days.	↓Tumour weigh; ↑necrosis; ↑apoptosis; ↑INF-γ and IL-4 (synergic effects between curcumin and metformin).	[155]
BALB\c mice; 4T1 xenograft	Curcumin (alone or together with arabinogalactan)	100 mg/kg/day; 32 days.	↓Tumour weight and volume; ↓Ki67; ↑p53.	[156]
BALB\c and athymic nude mice; BALB-neuT mammary cancer cells xenograft	Curcumin (alone or together with chloroquine)	2 mg/50 mL maize oil (3 times per week); Up to 35 days.	Effects of curcumin: ↓tumour volume (also in the presence of chloroquine); ↑mice survival; autophagy induction; ↓p62 protein level.	[157]
BALB/c nu/nu and athymic nude mice; MDA-MB-231, 4T1, JC, TuBo xenografts	Curcumin SMEDDS	100 mg/kg (p.o. 100 μL SMEDDS formulation); 1 – 2 months.	↓Tumour volumen.	[158]
BALB/c homozygous, Crl:NU(NCr)-Foxn1nu; MBCDF-T cells xenograft	Curcumin (alone or together with calcitriol)	40 mg/kg (p.o. every day); 3 weeks.	Effects of curcumin and calcitriol: ↓tumour volume ↓angiogenesis; ↓integrin expression.	[138]
Swiss albino mice; Ehrlich ascites carcinoma xenograft	Curcumin (alone or together with PAX or PAX + Vitamin D_3)	50 mg/kg (p.o. 3 times per week); 3 weeks.	↓Tumour volume and survival rate; ↑necrosis (after curcumin + PAX and Vitamin D3). ↓Tumour volume and survival rate; ↑necrosis; ↑MRD-1 (after curcumin + PAX).	[159]
		**Lignans/Flavonolignans**		
Sprague-Dawley rats; DMBA-induced BC	Flaxseed flour	5% (w/w) diet; PND 21–21 weeks after DMBA administration (PND 50).	↓Tumour size.	[160]
Sprague-Dawley rats; DMBA-induced BC	Flaxseed flour	5% (w/w) diet; 25 weeks on the diet.	Modulation of the n-3 and n-6 fatty acids in mammary gland and mammary tumour.	[161]
Sprague-Dawley rats; DMBA-induced BC	Sec-Dig, flaxseed oil and flaxseed	Sec-Dig (2.2 μmol/day (p.o. in water)), flaxeed oil (1.82% (w/w) diet), flaxeed (2.5 and 5% (w/w) diet); 7 weeks.	↓Tumour volume, incidence and number of tumours per rat; modulation of the n-3 and n-6	[162]

			fatty acids in mammary tumour.	
Sprague-Dawley rats; MNU-induced BC	Flaxseed and Sec-Dig	Flaxseed (2.5 and 5%) and Sec-Dig dose equivalent to that consumed in 2.5 (Low Sec-Dig) and 5% (High Sec-Dig) flaxseed groups; 22 weeks.	↓Tumour invasiveness; ↓poorly differentiated tumours. ↓Tumour multiplicity (high Sec-Dig) and ↑tumour multiplicity (low Sec-Dig).	[163]
Sprague-Dawley rats; MNU-induced BC	Flaxseed and Sec-Dig	Flaxseed (5% w/w diet) and Sec-Dig (1.5 mg equivalent to that consumed in flaxseed diet); 4 weeks.	↓IGF-1 level in plasma (effect in the presence or absence of NMU).	[164]
Sprague-Dawley rats; DMBA-induced BC	Flaxseed and Sec-Dig	Flaxseed (10% w/w diet) and Sec-Dig (20.1 mg/100 g diet; equivalent to that consumed in flaxseed diet); PND 0 – PND 21 (weaning).	↓Tumour incidence, burden, number, size, and weight.	[165]
Sprague-Dawley rats; DMBA-induced BC	Defatted flax flour	152 g/kg diet; GD 7 (in utero) – PND 0 (birth).	↑Apoptosis; ↑*p53* mRNA expression (but not protein).	[166]
Sprague-Dawley rats; DMBA-induced BC	Flaxseed	Flaxseed (5 and 10% w/w diet); 2 periods: GD 7 (in utero) – PND 0 (birth) and PND 5 – PND 25.	First period effects: ↑ER-α level (lobular and ductal tissue); ↓ER-β level (lobular tissue); ↑tumour multiplicity (at 10%); ↓latency. Second period effects: ↓ER-α level (lobular tissue); ↓ER-β level (TEB and lobular tissue); ↑tumour multiplicity and ↓latency (at 10%).	[167]
Germ-free Sprague-Dawley rats; DMBA-induced BC	Flaxseed	5% (w/w) diet; 13 weeks.	↓Tumour incidence and growth; ↓number of tumours per tumour bearing rat; ↓proliferation; ↑apoptosis; ↑enzyme activity in liver; and plasma: CAT, SOD, and GST (effects in gnotobiotic rats colonized with the lignan-converting bacteria[10] compared to germ-free rats).	[168]
Hemiovariectomized ACI rats; DMBA-induced BC	Sec-Dig	10 and 100 ppm; 3 months.	↑ESR1; ↑ESR2; ↑CDH2 and ↓*MKi-67* (only at 10 ppm).	[169]
TG.NK (MMTV/c-*neu*) transgenic mice	Flaxseed oil and Flaxseed oil + melatonin	Flaxseed oil (0.05, 0.1, and 0.2 mL; made up to 0.2 mL with corn oil) and flaxseed oil + melatonin (0.1 + 100 mg/kg b.w.; made up to 0.2 mL with corn oil); 30 weeks.	↓Tumour weight; ↓IGF-1 level in plasma; ↓number of tumours per mouse.	[170]

Athymic nude mice (Ncr *nu/nu*); MDA-MB-435 xenograft	Flaxseed	10% (w/w) diet; 8 weeks.	↓Tumour growth rate; ↓metastasis; ↓Ki-67 ↓IFG-I; ↓EGFR.	[171]
Athymic nude mice (Ncr *nu/nu*); MDA-MB-435 xenograft	Flaxseed	10% (w/w) diet; 7 weeks.	↓Tumour growth rate and metastasis; ↓VEGF.	[172]
Ovariectomized athymic mice BALB\c *nu/nu*; MCF-7 xenograft	Flaxseed (alone or together with TAM and in the presence of high/low doses of 17β-oestradiol)	Flaxseed (10% w/w diet); 6 and 7 weeks (at high and low doses of 17β-oestradiol, respectively).	↓Tumour volume, ↓Ki-67 level, and ↑apoptosis (flaxseed alone or together with TAM at 6 weeks); ↓tumour weight (together with TAM at 6 weeks). Attenuation of the ↑tumour volume and weight exerted by TAM at 7 weeks.	[173]
Athymic nude mice (Ncr *nu/nu*); MDA-MB-435 xenograft	Flaxseed, flaxseed oil, Sec-Dig, flaxseed oil + Sec-Dig	Flaxseed (10% w/w diet), flaxseed oil (36.53 g/kg diet), Sec-Dig (0.2 g/kg diet), flaxseed iol + Sec-Dig (36.53 + 0.2 g/kg diet); 6 weeks.	↓Metastasis incidence (including lung and lymph nodes). ↓tumour area; ↓apoptosis; ↓Ki-67 level; ↑MDA level in tumour.	[174]
Athymic nude mice (Ncr *nu/nu*); MDA-MB-435 xenograft	Flaxseed, flaxseed oil, Sec-Dig, flaxseed oil + Sec-Dig	Flaxseed (10% w/w diet), flaxseed oil (36.53 g/kg diet), Sec-Dig (0.2 g/kg diet), flaxseed iol + Sec-Dig (36.53 + 0.2 g/kg diet); 7 weeks.	↓Metastasis incidence (including lung and lymph nodes).	[175]
Ovariectomized athymic mice BALB\c *nu/nu*; MCF-7 xenograft	Flaxseed and Flaxseed + SPI	Flaxseed (10% w/w diet) and flaxseed + SPI (10% + 20% w/w diet); 19 weeks.	Flaxseed had no effect on tumour growth but attenuated the stimulating effect of SPI.	[176]
Ovariectomized athymic mice BALB\c *nu/nu*; MCF-7 xenograft	Flaxseed	10% (w/w) diet; 5 weeks.	↓Tumour growth and angiogenesis. ↓extracellular VEGF level.	[177]
Ovariectomized athymic mice BALB\c; MCF-7 xenograft	Flaxseed (alone or together with TAM)	5 and 10% (w/w) diet; 16 weeks.	Flaxseed alone exerted tumour size regression, whereas in the presences of TAM attenuated the stimulating growth effect of TAM; ↓cell proliferation; ↓apoptosis; ↓cyclin D1; ↓HER2; ↓IGF-IR; ↓ER-α.	[178]
Ovariectomized athymic mice BALB\c *nu/nu*; MCF-7 xenograft	Flaxseed (alone or together with TAM)	5 and 10% (w/w) diet; 8 weeks.	↓Tumour growth and proliferation; ↑apoptosis; ↑ER-α level; ↓PgR level; ↓IGF-I.	[179]

Ovariectomized athymic mice BALB\c *nu*/*nu*; MCF-7 xenograft	Flaxseed and Flaxseed + SPI	Flaxseed (10% w/w diet) and flaxseed + SPI (10% + 20% w/w diet); 2 or 25 weeks.	↓pMAPK level (at 2 weeks). ↑Proliferation; ↑cyclin D1; ↑ER-α level (effects at 25 weeks of flaxseed + SPI)	[180]
Athymic nude mice BALB\c; MDA-MB-231 xenograft	Flaxseed and Sec-Dig	Flaxseed (10% (w/w)) diet and Sec-Dig (dose equivalent to that consumed in flaxseed diet); 4 weeks.	↓Tumour growth. ↑Zn serum level (after flaxseed treatment); ↓Zn serum level (after Sec-Dig treatment); ↑gene expression of the Zn transporter *LIV-1* gene expression.	[181]
Ovariectomized athymic mice BALB\c *nu*/*nu*; MCF-7 xenograft	Flaxseed; Flaxseed hull; Sec-Dig	Flaxseed (10% w/w diet), flaxseed huul (1 g/kg diet), and Sec-Dig (18 g/kg diet); 7 weeks.	Effects of Flaxseed and Sec-Dig: Tumour regression; ↑apoptosis; ↓Bcl-2; ↓cyclin D1; ↓pS2; ↓ER-α and ER-β; ↓EGFR; ↓IGF-IR; ↓cell proliferation; ↓pMAPK (only Sec-Dig); ↓HER2 (only flaxseed). Effects of Flaxseed hull: ↓cell proliferation; ↑Bcl-2.	[182]
Athymic nude mice; MA782, MCF-7, T47D, MDA-MB-435s xenograft	Mix of neolignans isolated from *Vitex Negundo*	20, 40, and 80 mg/kg (p.o.); 16 or 20 days.	↓Tumour growth (dose-dependent).	[183]
Ovariectomized athymic mice BALB\c *nu*/*nu*; BT-474 xenograft	Flaxseed oil + trastuzumab	80 g/kg diet; 4 weeks.	↓Tumour growth; ↓proliferation; ↑apoptosis.	[184]
Ovariectomized athymic mice BALB\c *nu*/*nu*; MCF-7 xenograft	Flaxseed oil, Sec-Dig or flaxseed oil + Sec-Dig (all the treatments performed in the presence of TAM)	Flaxseed oil (38.5 g/kg diet), Sec-Dig (1 g/kg), and Flaxseed oil + Sec-Dig (38.5 and 1 g/kg diet); 7 weeks.	↓Tumour growth (only flaxseed oil); ↓proliferation; ↑apoptosis; modulation of ER- and growth factor mediated signaling pathways (mRNA and protein)[11].	[185]
Ovariectomized athymic mice BALB\c *nu*/*nu*; MCF-7 xenograft	Flaxseed oil, Sec-Dig or flaxseed oil + Sec-Dig	Flaxseed oil (38.5 g/kg diet), Sec-Dig (1 g/kg), and Flaxseed oil + Sec-Dig (38.5 and 1 g/kg diet); 7 weeks.	Tumour regression (in Sec-Dig and flaxseed oil treatments). ↓Tumour growth (only Sec-Dig treatmemt); ↓proliferation; modulation of ER- and growth factor mediated signaling pathways (mRNA and protein)[11].	[186]

Ovariectomized athymic mice BALB\c *nu/nu*; MCF-7 xenograft	Flaxseed oil	40 g/kg diet; 8 weeks.	↓Tumour growth; ↓proliferation; ↑apoptosis; ↓n-6/n-3 PUFA ratio; ↓p-MAPK, Akt and HER2 levels.	[187]
Tg.NK (MMTV/*c-neu*) transgenic mice	Flaxseed	60, 180, or 540 mg/kg diet; 2, 6 or 23 weeks.	↓Number of tumours per mouse; ↓tumour incidence (at 540 mg/kg diet); ↓GST (at 180 and 540 mg/kg diet); ↑cytochrome P450 enzymes CYP1A1 and 1A2 (at 60 mg/kg diet).	[188]
Ovariectomized athymic mice BALB\c *nu/nu*; MCF-7 xenograft	Cotyledon fraction isolated from flaxseed (alone or together with TAM)	82 g/kg diet; 7 weeks.	Induction of tumour regression; ↓cell proliferation; ↑AIB1 level; ↓HER2 and p-HER2 level; ↓ER*α* and p-ER*α* level; ↓*IGF-IR* mRNA expression; ↓*pS2* mRNA expression.	[189]
Athymic nude mice BALB\c *nu/nu*; MCF-7 xenograft	Flaxseed and enterolactone	Flaxseed (10% (w/w) diet) and enterolactone (100 mg/kg); ~20 days.	↓Tumour growth rate and angiogenesis; ↓murine IL-1β; ↑murine and human IL-1R*α*.	[190]
Ovariectomized athymic mice BALB\c *nu/nu*; MCF-7 xenograft	Sec-Dig and sesamin	Sec-Dig (1 g/kg) and sesamin (1 g/kg); 8 weeks.	↓Proliferation; ↓HER2 and EGFR (Sec-Dig treatment). ↓Tumour growth and proliferation; ↑apoptosis; ↓HER2, EGFR, and p-MAPK (sesamin treatment).	[191]
Ovariectomized athymic mice BALB\c *nu/nu*; BT-474 xenograft	Flaxseed (alone or together with trastuzumab)	10% (w/w) diet; 5 weeks.	Flaxseed + trastuzumab: ↓Tumour growth; ↑survival rate; ↓p-Akt1 protein level; ↓*HER2* mRNA expression ↓n-6/n-3 PUFA ratio.	[192]
Ovariectomized athymic mice BALB\c *nu/nu*; BT-474 xenograft	Flaxseed oil (alone or together with trastuzumab)	4% (w/w) diet; 4 weeks.	Effects of flaxseed oil + trastuzumab: ↓tumour growth and proliferation; ↑apoptosis; ↓*ERBB2*, *EGFR*, and *MAPK2*; mRNA expression; ↓p-HRE2 protein level; ↓n-6/n-3 PUFA ratio.	[193]

Swiss albino mice; Erlhlich ascites carcinoma	Flaxseed, flaxseed meal and flaxseed oil	10% each treatment; 3 weeks.	↑Tumour latency; ↓ER level; ↓Ki-67, PgR, IGF, VEGF and MMP-2; ↑caspase-3.	[194]
C57BL/6J mice; DMBA-induced BC	Flaxseed oil	78 g/kg diet; from gestation – PND 21.	↑Tumour incidence in presence of carcinogen. ↑TEB; accelerated puberty onset; ↑17β-oestradiol level; ↑mammary gland cell proliferation; modulation of cancer-associated KEGG pathways (in absence of carcinogen).	[195]
C57BL/6J mice; E0771 xenograft	Sec-Dig	25, 74, and 100 mg/kg diet; 11 weeks.	Non-tumour bearing mammary glan: ↓Crown-like structures; ↓*ADGRE1* and *Ccl2* mRNA expression. Tumour bearing mammary gland: ↓Tumour growth; ↓*ADGRE1* mRNA expression; ↓p-p65; modulation of NF-ƙB target genes expression.	[196]
Sprague-Dawley rats; MNU-induced BC and MMTV-neu-transgenic mice	Silymarin	Rats: 0.03, 0.1, 0.3, and 1% (w/w) diet; PND 21 – PND 161 (140 days). Mice: 0.3% (w/w) diet; PND 28 or 120 – PND ~10 months.	↑Number of tumours (in rats). ↑Breast tumour incidence and multiplicity (in mice).	[197]
FVB/N HER-2/neu transgenic mice	Silybin-phosphatidylcholine complex (IdB 1016)	450 mg/kg (equivalent to 414 μM/kg silybin); administered daily or twice a week for 1 month.	↓Tumour incidence, multiplicity and lung metastases; ↓HER/*neu* gene expression; ↑senescence; ↑infiltration of neutrophils, CD4 and CD8 cells.	[198]
C3(1) SV40 T,t antigen transgenic multiple mammary adenocarcinoma mouse	Silybin or Silipide	0.2% (w/w) diet; up to 177 days.	No effects described.	[199]
BALB\c and SCDI CB17-Prkdcscid/J mice; 4T1 xenograft	Silybin	150 mg/kg (p.o., 3 times per week); 4 – 5 weeks.	↑Survival rate; ↓tumour growth; ↓MDSC accumulation; ↑T-cell accumulation; ↑TNF-α and IL-1β; ↓IL-10 and CCR2 (in BALB\c). No effects described in SCDI mice.	[200]

BALB\c nude mice; MDA-MB-468	Silybin	200 mg/kg; 45 days.	↓Tumour volume; ↓*COX-2*, *MMP-9*, and *VEGF* gene expression; ↓COX-2 and VEGF protein level.	[201]
Sprague-Dawley rats; DMBA-induced BC	Silymarin	Basal diet supplemented with 0.5% (w/w) of silymarin nanoparticles; 6 months	↑8-OHdG; ↑apoptosis; ↓ErbB-2 level in plasma; ↓total antioxidant capacity.	[202]
C57BL/6; Ca 755 xenograft	Silybin (free or mixture with lecithin)	20 mg/kg b.w. (p.o.); 10 days.	↑Lifespan.	[203]
**Hydrolysable tannins**				
Sprague-Dawley rats; DMBA-induced BC	EA (alone or together with selenomethionine)	0.4 g/kg diet; 25 weeks.	↓Cumulative number of tumours (only significant in the presence of diallyl sulfide).	[204]
F344 rats; PhIP-induced BC	EA	0.1% (w/w) diet; up to 52 weeks.	No significant effects.	[105]
F344 rats; PhIP-induced BC	EA	0.1% (w/w) diet; up to 52 weeks.	No significant effects.	[110]
ACI rats; 17β-oestradiol-induced BC	Dehydrated powered blue berry, freeze-dried black raspberry and EA	Dehydrated powered blue berry (1 and 2.5% (w/w) diet); freeze-dried black raspberry (1 and 2.5% (w/w) diet) and EA (400 ppm); up to 26 weeks.	↓Tumour incidence and multiplicity; ↓*CYP1A1* and *17β-HSD7* expression; ↓COMT (in blue berry treatment). ↓Tumour incidence, growth and incidence; ↑mammary and pituitary weight; ↓*CYP1A1* and *CYP1B1* expression; ↓COMT (in black raspberry treatment). ↓Tumour incidence, volume and multiplicity; ↓*17β-HSD7* expression; ↓COMT (in EA treatment).	[205]
ACI rats; 17β-oestradiol-induced BC	EA (alone or together with 17β-oestradiol)	400 ppm; up to 28 weeks.	Amelioration of the dysregulation induced by 17β-oestradiol on miRNA expression (miR-206, miR-182, miR-375, miR-127, miR-183, miR-122) and their targets at gene and protein level (ERα, cyclin D1, RASD1, FoxO3a, FoxO1, cyclin G1, Bcl-w, and Bcl-2).	[206]

Sprague-Dawley rats; DMBA-induced BC	Pom emulsion	0.2, 1, and 5 g/Kg (p.o. 3 times a week); 18 weeks.	↓ER-α, ER-β, and ER-α/ER-β; ↓β-catenin expression and cytoplasmic accumulation; ↓cyclin D1 expression.	[207]
Sprague-Dawley rats; DMBA-induced BC	Pom emulsion	0.2, 1, and 5 g/Kg (p.o. 3 times a week); 18 weeks.	↓Tumour incidence and weight; ↓proliferation; ↑apoptosis, Bax/Bcl-2 (mRNA and protein), Bad, caspase-3, caspase-7, caspase-9, PARP, and cytochrome-c.	[208]
Swiss albino mice; Ehrlich ascites carcinoma cells injection	EA	3 mg/kg; 50 days.	↓Tumour growth.	[48]
BALB/c athymic nude mice; MDA-MB-231 xenograft	Penta-*O*-galloyl-β-D-glucose	20 mg/kg/day b.w. (p.o.); 36 days.	↓Tumour growth.	[209]
BALB/c athymic nude mice; MDA-MB-231 xenograft	Penta-*O*-galloyl-β-D-glucose	10 mg/kg/day b.w. (p.o.); 36 days.	↓Tumour growth and metastasis; ↓Ki-67, CD34, p-STAT, and VEGF level; ↑apoptosis.	[210]
BALB/c athymic mice (*nu/nu*); MDA-MB-468	Gallotannin	0.5% in drinking water (w/v) or daily i.p. injections (10 mg/kg, 5 injections per week); 30 days.	↓Tumour growth; ↓CcnD1 protein level; ↑p-Chk2.	[211]
SCDI mice; MDA-MB-231 luciferase 2 xenograft	EA (alone or together with MRS2179)	120 μg/mL in drinking water; 16 weeks.	↓Tumour growth and lung metastasis.	[212]
MMTV-PyMT transgenic mice	EA	50 mg/kg/day (p.o.); 15 weeks.	↓Tumour growth, burden and lung metastasis; ↑tumour latency and survival rate; ↓cancer stem cells population.	[213]

[1]Hexokinase, phosphoglucoisomerase, aldolase, glucose-6-phosphate, fructose-1,6-diphosphate, and fructose-6-phosphate; [2]Total, free and ester cholesterol, phospholipids, triglycerides, free fatty acids; [3]Na^+K^+-, Ca^{+2}-, and Mg^{+2}-ATPase; [4]Hesperetin:MAN:SLS:DOSS (2.00:7.74:0.20:0.06); [5]TBARS, GSH, SOD, nitrate, protein carbonyl, catalase, GR, vitamin C and E; [6]Bcl-xL, Bcl-2, Bad, cytochrome C, VDAC, Bax, Apaf-1, and pro-caspase-9; [7]MCF-7aro: MCF-7 breast cancer cells transfected with human CYP19; [8]Vincristine was loaded into ESM/cholesterol/PEG_{2000}-ceramide/Quer (72.5:17.5:5:5); [9]MCF-7Ca: aromatase-expressing MCF-7 cells; [10]*Clostridium saccharogumia*, *Eggerthella lenta*, *Blautia producta*, and *Lactonifactor longoviformis*; [11]mRNA: *pS2*, *PgR*, *ERα*, *ERβ*, *CD1*, *EGFR*, *IGF-IR*, *Bcl2*, *HER2*; proteins: ERα, HER2, MAPK, AIB1, Akt.

Abbreviations: ADGRE1, adhesion G protein-coupled receptor 1; AFP, alpha-pheto protein; AIB1, amplified in breast 1; AhRR, aromatic hydrocarbon receptor repressor; α-SMA, alpha smooth muscle actin; Bad, Bcl-2 associated agonist of cell death; BC, breast cancer; bFGF, basic fibrobast growth factor; BRCA1, Breast Cancer Type 1 susceptibility protein; BT, black tea; b.w., body weight; CA 15-3, breast cancer specific marker; Cath, catechin; CCR2, C-C chemokine receptor type 2; CEA, carcinoembryonic antigen; CK, cytokeratin; CoenzQ10, co-enzyme Q10; COMT, cathecol-*O*-methyltransferase; COX-2, cyclooxygenase-2; DAZ, Daidzein; DHA, docosahexahenoic acid; DMBA, 7,12-dimethylbenz[α]antracen; DNMT, DNA methyltransferase; DOX, doxorubicin; EA, ellagic acid; EGCG, epigallocatechin gallate; EGFR, epithelial growth factor receptor; ER, oestrogen receptor; ERα, oestrogen receptor alpha; ERβ, oestrogen receptor beta; EROD, Ethoxyresorfurin-*O*-deethylase; ESM, egg sphingomyelin; EXM, exemestrane; Exp., experiment; FoxO, Forkhead box; GD, gestation day; GEN, genistein; Gli-1, Zinc finger protein; GLY, glycetein; GPx, glutathione peroxidase; GSI, genistin-rich soy isoflavone mixture; GST, glutathione S-transferase; GT,

green tea; GTE, greent tea extract; 17β-HSD7, 17β-dehydrogenase 7; HER2, human epidermal growth factor receptor 2; HDAC: histone deacetylase; 8-OHdG, 8-hydroxy-2'-deoxyguanoside; HOTAIR, HOX transcript antisense RNA; IDSP, isoflavone-depleted soy protein; IGF-1, insulin-like growth factor 1; IGF-IR, insulin-like growth factor receptor; IPSP, isoflavone-poor soy protein; IRF-1, interferon regulatory factor 1; LH, luteinizing hormone; LTB4, leukotriene B4; LYVE-1, lymphatic vessel endothelial hyaluronan receptor 1; MDA, malondialdehyde; MDSC, myeloid-derived suppressor cells; MMP, metalloproteinase; MPA, medroxyprogesterone acetate; MROD, methoxyresorufin-*O*-demethylase; NMU, N-nitroso-N-methylurea; NPs, nanoparticles; NSD, Nanocrystalline solid dispersions; PARP, poly(ADP) ribose polymerase; PAX, paclitaxel; PCNA, proliferating cell nuclear antigen; PECAM-1, platelet endothelial adhesion molecule 1; PEG2000, polyglycerol stearate; pER*α*, phospho-oestrogen receptor alpha; PgR, progesterone receptor; PhIP, 2-amino-1-methyl-6-phenylimidazo[4,5-b]pyridine; PKC, protein kinase C; PM, physical mixture; pMAPK, phospho-specific mitogen-activated protein kinase; PND, postnatal day; Pom, pomegranate; pS2, marker for hormone-dependent breast cancer; p.o., *per os* (oral administration); PTEN, phosphatase and tensin homolog; PUFA, polyunsaturated fatty acid; Quer, quercetin; RASD1, RAS dexamethasone-induced 1; RQC, RSV + Quer + Cath; s.c., subcutaneous; RSV, resveratrol; Sec-Dig, secoisolariciresinol diglycoside; SCID, Severe Combined Immune-Deficient; SMEDDS, self-microemulsifying drug delivery system; SNEDDS, self-nanoemulsifying drug delivery system; SP, soy protein; SPC, soy phytochemical concentrate; SPE, soy phytochemical extraction; SPI, soy protein isolate; SPId, soy protein isolate depleted of isoflavones; SSH, sonic hedgehog; SOD, superoxide dismutase; TAM, tamoxifen; TBARS, thiobarbituric acid reactive substances; TCDD, 2,3,7,8-tetrachlorodibenzo-p-dioxin; TD, terminal duct; TEB, terminal end bud; TGF-β1, transforming growth factor-β1; TNF-*α*, tumour necrosis factor alpha; TRAF, TNF receptor associated factors; VEGF, vascular endothelial growth factor; XO, xanthine oxidase.

References

1. Jung, K.-J.; Wallig, M.A.; Singletary, K.W. Purple grape juice inhibits 7,12-dimethylbenz[a]anthracene (DMBA)-induced rat mammary tumorigenesis and in vivo DMBA-DNA adduct formation. *Cancer Lett.* **2006**, *233*, 279–288, doi:10.1016/j.canlet.2005.03.020.
2. Thiele, W.; Rothley, M.; Teller, N.; Jung, N.; Bulat, B.; Plaumann, D.; Vanderheiden, S.; Schmaus, A.; Cremers, N.; Göppert, B.; et al. Delphinidin is a novel inhibitor of lymphangiogenesis but promotes mammary tumor growth and metastasis formation in syngeneic experimental rats. *Carcinogenesis* **2013**, *34*, 2804–2813, doi:10.1093/carcin/bgt291.
3. Han, B.; Peng, X.; Cheng, D.; Zhu, Y.; Du, J.; Li, J.; Yu, X. Delphinidin suppresses breast carcinogenesis through the HOTAIR /micro RNA -34a axis. *Cancer Sci.* **2019**, *110*, 3089–3097, doi:10.1111/cas.14133.
4. Hui, C.; Bin, Y.; Xiaoping, Y.; Long, Y.; Chunye, C.; Mantian, M.; Wenhua, L. Anticancer Activities of an Anthocyanin-Rich Extract From Black Rice Against Breast Cancer Cells In Vitro and In Vivo. *Nutr. Cancer* **2010**, *62*, 1128–1136, doi:10.1080/01635581.2010.494821.
5. Luo, L.-P.; Han, B.; Yu, X.-P.; Chen, X.-Y.; Zhou, J.; Chen, W.; Zhu, Y.-F.; Peng, X.-L.; Zou, Q.; Li, S.-Y. Anti-metastasis Activity of Black Rice Anthocyanins Against Breast Cancer: Analyses Using an ErbB2 Positive Breast Cancer Cell Line and Tumoral Xenograft Model. *Asian Pac. J. Cancer Prev.* **2014**, *15*, 6219–6225, doi:10.7314/APJCP.2014.15.15.6219.
6. Yang, X.; Luo, E.; Liu, X.; Han, B.; Yu, X.; Peng, X. Delphinidin-3-glucoside suppresses breast carcinogenesis by inactivating the Akt/HOTAIR signaling pathway. *BMC Cancer* **2016**, *16*, 423, doi:10.1186/s12885-016-2465-0.
7. Cho, E.; Chung, E.Y.; Jang, H.-Y.; Hong, O.-Y.; Chae, H.S.; Jeong, Y.-J.; Kim, S.-Y.; Kim, B.-S.; Yoo, D.J.; Kim, J.-S.; et al. Anti-cancer Effect of Cyanidin-3-glucoside from Mulberry via Caspase-3 Cleavage and DNA Fragmentation in vitro and in vivo. *Anticancer Agents Med. Chem.* **2017**, *17*, doi:10.2174/1871520617666170327152026.
8. So, F.V.; Guthrie, N.; Chambers, A.F.; Moussa, M.; Carroll, K.K. Inhibition of human breast cancer cell proliferation and delay of mammary tumorigenesis by flavonoids and citrus juices. *Nutr. Cancer* **1996**, *26*, 167–181, doi:10.1080/01635589609514473.
9. Nandakumar, N.; Rengarajan, T.; Balamurugan, A.; Balasubramanian, M. Modulating effects of hesperidin on key carbohydrate-metabolizing enzymes, lipid profile, and membrane-bound adenosine triphosphatases against 7,12-dimethylbenz(*a*)anthracene-induced breast carcinogenesis. *Hum. Exp. Toxicol.* **2014**, *33*, 504–516, doi:10.1177/0960327113485252.
10. Sandhu, P.S.; Kumar, R.; Beg, S.; Jain, S.; Kushwah, V.; Katare, O.P.; Singh, B. Natural lipids enriched self-nano-emulsifying systems for effective co-delivery of tamoxifen and naringenin: Systematic approach for improved breast cancer therapeutics. *Nanomedicine Nanotechnol. Biol. Med.* **2017**, *13*, 1703–1713, doi:10.1016/j.nano.2017.03.003.
11. Sheokand, S.; Navik, U.; Bansal, A.K. Nanocrystalline solid dispersions (NSD) of hesperetin (HRN) for prevention of 7, 12-dimethylbenz[a]anthracene (DMBA)-induced breast cancer in Sprague-Dawley (SD) rats. *Eur. J. Pharm. Sci.* **2019**, *128*, 240–249, doi:10.1016/j.ejps.2018.12.006.
12. Zhao, Z.; Jin, G.; Ge, Y.; Guo, Z. Naringenin inhibits migration of breast cancer cells via inflammatory and apoptosis cell signaling pathways. *Inflammopharmacology* **2019**, *27*, 1021–1036, doi:10.1007/s10787-018-00556-3.
13. Qin, L.; Jin, L.; Lu, L.; Lu, X.; Zhang, C.; Zhang, F.; Liang, W. Naringenin reduces lung metastasis in a breast cancer resection model. *Protein Cell* **2011**, *2*, 507–516, doi:10.1007/s13238-011-1056-8.
14. Ye, L.; Chan, F.L.; Chen, S.; Leung, L.K. The citrus flavonone hesperetin inhibits growth of aromatase-expressing MCF-7 tumor in ovariectomized athymic mice. *J. Nutr. Biochem.* **2012**, *23*, 1230–1237, doi:10.1016/j.jnutbio.2011.07.003.
15. Li, F.; Chow, S.; Cheung, W.; Chan, F.L.; Chen, S.; Leung, L.K. The citrus flavonone hesperetin prevents letrozole-induced bone loss in a mouse model of breast cancer. *J. Nutr. Biochem.* **2013**, *24*, 1112–1116, doi:10.1016/j.jnutbio.2012.08.010.
16. Zhang, F.; Dong, W.; Zeng, W.; Zhang, L.; Zhang, C.; Qiu, Y.; Wang, L.; Yin, X.; Zhang, C.; Liang, W. Naringenin prevents TGF-β1 secretion from breast cancer and suppresses pulmonary metastasis by inhibiting PKC activation. *Breast Cancer Res.* **2016**, *18*, 38, doi:10.1186/s13058-016-0698-0.
17. Ke, J.-Y.; Banh, T.; Hsiao, Y.-H.; Cole, R.M.; Straka, S.R.; Yee, L.D.; Belury, M.A. Citrus flavonoid naringenin reduces mammary tumor cell viability, adipose mass, and adipose inflammation in obese ovariectomized mice. *Mol. Nutr. Food Res.* **2017**, *61*, 1600934, doi:10.1002/mnfr.201600934.

18. Singhal, J.; Chikara, S.; Horne, D.; Salgia, R.; Awasthi, S.; Singhal, S.S. 2′-Hydroxyflavanone inhibits in vitro and in vivo growth of breast cancer cells by targeting RLIP76. *Mol. Carcinog.* **2018**, *57*, 1751–1762, doi:10.1002/mc.22894.
19. Singhal, J.; Chikara, S.; Horne, D.; Salgia, R.; Awasthi, S.; Singhal, S.S. RLIP inhibition suppresses breast-to-lung metastasis. *Cancer Lett.* **2019**, *447*, 24–32, doi:10.1016/j.canlet.2019.01.023.
20. Mafuvadze, B.; Cook, M.; Xu, Z.; Besch-Williford, C.L.; Hyder, S.M. Effects of Dietary Apigenin on Tumor Latency, Incidence and Multiplicity in a Medroxyprogesterone Acetate- Accelerated 7,12-Dimethylbenz(a)anthracene-Induced Breast Cancer Model. *Nutr. Cancer* **2013**, *65*, 1184–1191, doi:10.1080/01635581.2013.833637.
21. Lee, H.H.; Jung, J.; Moon, A.; Kang, H.; Cho, H. Antitumor and Anti-Invasive Effect of Apigenin on Human Breast Carcinoma through Suppression of IL-6 Expression. *Int. J. Mol. Sci.* **2019**, *20*, 3143, doi:10.3390/ijms20133143.
22. Samy, R.P.; Gopalakrishnakone, P.; Ignacimuthu, S. Anti-tumor promoting potential of luteolin against 7,12-dimethylbenz(a)anthracene-induced mammary tumors in rats. *Chem. Biol. Interact.* **2006**, *164*, 1–14, doi:10.1016/j.cbi.2006.08.018.
23. Du, G.-J.; Song, Z.-H.; Lin, H.-H.; Han, X.; Zhang, S.; Yang, Y. Luteolin as a glycolysis inhibitor offers superior efficacy and lesser toxicity of doxorubicin in breast cancer cells. *Biochem. Biophys. Res. Commun.* **2008**, *372*, 497–502, doi:10.1016/j.bbrc.2008.05.080.
24. Lee, E.-J.; Oh, S.-Y.; Sung, M.-K. Luteolin exerts anti-tumor activity through the suppression of epidermal growth factor receptor-mediated pathway in MDA-MB-231 ER-negative breast cancer cells. *Food Chem. Toxicol.* **2012**, *50*, 4136–4143, doi:10.1016/j.fct.2012.08.025.
25. Li, F.; Wong, T.Y.; Lin, S.; Chow, S.; Cheung, W.; Chan, F.L.; Chen, S.; Leung, L.K. Coadministrating Luteolin Minimizes the Side Effects of the Aromatase Inhibitor Letrozole. *J. Pharmacol. Exp. Ther.* **2014**, *351*, 270–277, doi:10.1124/jpet.114.216754.
26. Ma, X.-C.; Yan, W.; Dai, Z.; Gao, X.; Ma, Y.; Xu, Q.; Jiang, J.; Zhang, S. Baicalein suppresses metastasis of breast cancer cells by inhibiting EMT via downregulation of SATB1 and Wnt/β-catenin pathway. *Drug Des. Devel. Ther.* **2016**, 1419, doi:10.2147/DDDT.S102541.
27. Yan, W.; Ma, X.; Zhao, X.; Zhang, S. Baicalein induces apoptosis and autophagy of breast cancer cells via inhibiting PI3K/AKT pathway in vivo and vitro. *Drug Des. Devel. Ther.* **2018**, *Volume 12*, 3961–3972, doi:10.2147/DDDT.S181939.
28. Chung, H.; Jung, Y.; Shin, D.-H.; Lee, J.-Y.; Oh, M.-Y.; Kim, H.-J.; Jang, K.S.; Jeon, S.J.; Son, K.H.; Kong, G. Anticancer effects of wogonin in both estrogen receptor-positive and -negative human breast cancer cell lines *in vitro* and in nude mice xenografts. *Int. J. Cancer* **2008**, *122*, 816–822, doi:10.1002/ijc.23182.
29. Yao, Y.; Zhao, K.; Yu, Z.; Ren, H.; Zhao, L.; Li, Z.; Guo, Q.; Lu, N. Wogonoside inhibits invasion and migration through suppressing TRAF2/4 expression in breast cancer. *J. Exp. Clin. Cancer Res.* **2017**, *36*, 103, doi:10.1186/s13046-017-0574-5.
30. Huang, Y.; Fang, J.; Lu, W.; Wang, Z.; Wang, Q.; Hou, Y.; Jiang, X.; Reizes, O.; Lathia, J.; Nussinov, R.; et al. A Systems Pharmacology Approach Uncovers Wogonoside as an Angiogenesis Inhibitor of Triple-Negative Breast Cancer by Targeting Hedgehog Signaling. *Cell Chem. Biol.* **2019**, *26*, 1143-1158.e6, doi:10.1016/j.chembiol.2019.05.004.
31. Hirose, M.; Masuda, A.; Fukushima, S.; Ito, N. Effects of subsequent antioxidant treatment on 7,12-dimethylbenz[a]anthracene-initiated carcinogenesis of the mammary gland, ear duct and forestomach in Sprague-Dawley rats. *Carcinogenesis* **1988**, *9*, 101–104, doi:10.1093/carcin/9.1.101.
32. Verma, A.K.; Johnson, J.A.; Gould, M.N.; Tanner, M.A. Inhibition of 7,12-dimethylbenz(a)anthracene- and N-nitrosomethylurea-induced rat mammary cancer by dietary flavonol quercetin. *Cancer Res.* **1988**, *48*, 5754–5758.
33. Ip, C.; Ganther, H.E. Combination of blocking agents and suppressing agents in cancer prevention. *Carcinogenesis* **1991**, *12*, 365–367, doi:10.1093/carcin/12.2.365.
34. Pereira, M.A.; Grubbs, C.J.; Barnes, L.H.; Li, H.; Olson, G.R.; Eto, I.; Juliana, M.; Whitaker, L.M.; Kelloff, G.J.; Steele, V.E.; et al. Effects of the phytochemicals, curcumin and quercetin, upon azoxymethane-induced colon cancer and 7, 12-dimethylbenz[*a*]anthracene-induced mammary cancer in rats. *Carcinogenesis* **1996**, *17*, 1305–1311, doi:10.1093/carcin/17.6.1305.
35. Singh, B.; Mense, S.M.; Bhat, N.K.; Putty, S.; Guthiel, W.A.; Remotti, F.; Bhat, H.K. Dietary quercetin exacerbates the development of estrogen-induced breast tumors in female ACI rats. *Toxicol. Appl. Pharmacol.* **2010**, *247*, 83–90, doi:10.1016/j.taap.2010.06.011.
36. Reddy, N.S.; Nirmala, P.J.; Chidambaram, N.; Kumar, P.A. Quercetin in Dimethyl Benzanthracene Induced Breast Cancer in Rats. **2012**, doi:10.3844/ajptsp.2012.68.72.

37. Jain, A.K.; Thanki, K.; Jain, S. Co-encapsulation of Tamoxifen and Quercetin in Polymeric Nanoparticles: Implications on Oral Bioavailability, Antitumor Efficacy, and Drug-Induced Toxicity. *Mol. Pharm.* **2013**, *10*, 3459–3474, doi:10.1021/mp400311j.
38. Jain, A.K.; Thanki, K.; Jain, S. Novel self-nanoemulsifying formulation of quercetin: Implications of pro-oxidant activity on the anticancer efficacy. *Nanomedicine Nanotechnol. Biol. Med.* **2014**, *10*, 959–969, doi:10.1016/j.nano.2013.12.010.
39. Jain, A.K.; Thanki, K.; Jain, S. Solidified self-nanoemulsifying formulation for oral delivery of combinatorial therapeutic regimen: part II in vivo pharmacokinetics, antitumor efficacy and hepatotoxicity. *Pharm. Res.* **2014**, *31*, 946–958, doi:10.1007/s11095-013-1214-1.
40. Santhalakshmi, R.; Ramakrishna, Y.G. PROTECTIVE EFFECT OF QUERCETIN AGAINST 7, 12 DIMETHYLBENZ (A)-ANTHRACENE INDUCED BREAST CANCER IN RATS Available online: /paper/PROTECTIVE-EFFECT-OF-QUERCETIN-AGAINST-7%2C-12-BREAST-Santhalakshmi-Ramakrishna/84495822add2bebdd20da05cf0aa3b3d0fad7f02 (accessed on Jul 8, 2020).
41. Tripathi, S.; Kushwah, V.; Thanki, K.; Jain, S. Triple antioxidant SNEDDS formulation with enhanced oral bioavailability: Implication of chemoprevention of breast cancer. *Nanomedicine Nanotechnol. Biol. Med.* **2016**, *12*, 1431–1443, doi:10.1016/j.nano.2016.03.003.
42. Ramalingayya, G.V.; Gourishetti, K.; Nayak, P.G.; Rao, C.M.; Kishore, A.; Alnaseer, S.M.; Hussain, S.M.; Nandakumar, K. Rutin Protects against Doxorubicin-Induced Cognitive Dysfunction While Retaining the Anticancer Potential of Dox in a Murine Model of N-Methyl-N-Nitrosourea – Induced Mammary Carcinoma. *J. Environ. Pathol. Toxicol. Oncol.* **2019**, *38*, 153–163, doi:10.1615/JEnvironPatholToxicolOncol.2019028294.
43. Schlachterman, A.; Valle, F.; Wall, K.M.; Azios, N.G.; Castillo, L.; Morell, L.; Washington, A.V.; Cubano, L.A.; Dharmawardhane, S.F. Combined Resveratrol, Quercetin, and Catechin Treatment Reduces Breast Tumor Growth in a Nude Mouse Model. *Transl. Oncol.* **2008**, *1*, 19–27, doi:10.1593/tlo.07100.
44. Du, G.; Lin, H.; Yang, Y.; Zhang, S.; Wu, X.; Wang, M.; Ji, L.; Lu, L.; Yu, L.; Han, G. Dietary quercetin combining intratumoral doxorubicin injection synergistically induces rejection of established breast cancer in mice. *Int. Immunopharmacol.* **2010**, *10*, 819–826, doi:10.1016/j.intimp.2010.04.018.
45. Du, G.; Lin, H.; Wang, M.; Zhang, S.; Wu, X.; Lu, L.; Ji, L.; Yu, L. Quercetin greatly improved therapeutic index of doxorubicin against 4T1 breast cancer by its opposing effects on HIF-1α in tumor and normal cells. *Cancer Chemother. Pharmacol.* **2010**, *65*, 277–287, doi:10.1007/s00280-009-1032-7.
46. Wong, M.-Y.; Chiu, G.N.C. Liposome formulation of co-encapsulated vincristine and quercetin enhanced antitumor activity in a trastuzumab-insensitive breast tumor xenograft model. *Nanomedicine Nanotechnol. Biol. Med.* **2011**, *7*, 834–840, doi:10.1016/j.nano.2011.02.001.
47. Steiner, J.; Davis, J.; McClellan, J.; Enos, R.; Carson, J.; Fayad, R.; Nagarkatti, M.; Nagarkatti, P.; Altomare, D.; Creek, K.; et al. Dose-dependent benefits of quercetin on tumorigenesis in the C3(1)/SV40Tag transgenic mouse model of breast cancer. *Cancer Biol. Ther.* **2014**, *15*, 1456–1467, doi:10.4161/15384047.2014.955444.
48. Srivastava, S.; Somasagara, R.R.; Hegde, M.; Nishana, M.; Tadi, S.K.; Srivastava, M.; Choudhary, B.; Raghavan, S.C. Quercetin, a Natural Flavonoid Interacts with DNA, Arrests Cell Cycle and Causes Tumor Regression by Activating Mitochondrial Pathway of Apoptosis. *Sci. Rep.* **2016**, *6*, 24049, doi:10.1038/srep24049.
49. Zhao, X.; Wang, Q.; Yang, S.; Chen, C.; Li, X.; Liu, J.; Zou, Z.; Cai, D. Quercetin inhibits angiogenesis by targeting calcineurin in the xenograft model of human breast cancer. *Eur. J. Pharmacol.* **2016**, *781*, 60–68, doi:10.1016/j.ejphar.2016.03.063.
50. Roy, S.; Banerjee, S.; Chakraborty, T. Vanadium quercetin complex attenuates mammary cancer by regulating the P53, Akt/mTOR pathway and downregulates cellular proliferation correlated with increased apoptotic events. *BioMetals* **2018**, *31*, 647–671, doi:10.1007/s10534-018-0117-3.
51. Jayakumar, J.; Nirmala, P.; Praveen Kumar, B.; Kumar, A. Evaluation of protective effect of myricetin, a bioflavonoid in dimethyl benzanthracene-induced breast cancer in female Wistar rats. *South Asian J. Cancer* **2014**, *3*, 107, doi:10.4103/2278-330X.130443.
52. Fritz, W. Dietary genistein: perinatal mammary cancer prevention, bioavailability and toxicity testing in the rat. *Carcinogenesis* **1998**, *19*, 2151–2158, doi:10.1093/carcin/19.12.2151.
53. Appelt, L.C.; Reicks, M.M. Soy Induces Phase II Enzymes But Does Not Inhibit Dimethylbenz[a]anthracene-Induced Carcinogenesis in Female Rats. *J. Nutr.* **1999**, *129*, 1820–1826, doi:10.1093/jn/129.10.1820.

54. Cohen, L.A. Effect of intact and isoflavone-depleted soy protein on NMU-induced rat mammary tumorigenesis. *Carcinogenesis* **2000**, *21*, 929–935, doi:10.1093/carcin/21.5.929.
55. Hakkak, R.; Korourian, S.; Shelnutt, S.R.; Lensing, S.; Ronis, M.J.; Badger, T.M. Diets containing whey proteins or soy protein isolate protect against 7,12-dimethylbenz(a)anthracene-induced mammary tumors in female rats. *Cancer Epidemiol. Biomark. Prev. Publ. Am. Assoc. Cancer Res. Cosponsored Am. Soc. Prev. Oncol.* **2000**, *9*, 113–117.
56. Gallo, D.; Giacomelli, S.; Cantelmo, F.; Zannoni, G.F.; Ferrandina, G.; Fruscella, E.; Riva, A.; Morazzoni, P.; Bombardelli, E.; Mancuso, S.; et al. Chemoprevention of DMBA-induced mammary cancer in rats by dietary soy. *Breast Cancer Res. Treat.* **2001**, *69*, 153–164, doi:10.1023/A:1012414119944.
57. Constantinou, A.I.; Lantvit, D.; Hawthorne, M.; Xu, X.; van Breemen, R.B.; Pezzuto, J.M. Chemopreventive Effects of Soy Protein and Purified Soy Isoflavones on DMBA-Induced Mammary Tumors in Female Sprague-Dawley Rats. *Nutr. Cancer* **2001**, *41*, 75–81, doi:10.1080/01635581.2001.9680615.
58. Rowlands, J.C.; He, L.; Hakkak, R.; Ronis, M.J.J.; Badger, T.M. Soy and Whey Proteins Downregulate DMBA-Induced Liver and Mammary Gland CYP1 Expression in Female Rats. *J. Nutr.* **2001**, *131*, 3281–3287, doi:10.1093/jn/131.12.3281.
59. Rowlands, J.C. Altered Mammary Gland Differentiation and Progesterone Receptor Expression in Rats Fed Soy and Whey Proteins. *Toxicol. Sci.* **2002**, *70*, 40–45, doi:10.1093/toxsci/70.1.40.
60. Hilakivi-Clarke, L.; Cho, E.; Cabanes, A.; DeAssis, S.; Olivo, S.; Helferich, W.; Lippman, M.E.; Clarke, R. Dietary modulation of pregnancy estrogen levels and breast cancer risk among female rat offspring. *Clin. Cancer Res. Off. J. Am. Assoc. Cancer Res.* **2002**, *8*, 3601–3610.
61. Lamartiniere, C.A. Daidzein: Bioavailability, Potential for Reproductive Toxicity, and Breast Cancer Chemoprevention in Female Rats. *Toxicol. Sci.* **2002**, *65*, 228–238, doi:10.1093/toxsci/65.2.228.
62. Ueda, M.; Niho, N.; Imai, T.; Shibutani, M.; Mitsumori, K.; Matsui, T.; Hirose, M. Lack of Significant Effects of Genistein on the Progression of 7,12-dimethylbenz(a)anthracene-Induced Mammary Tumors in Ovariectomized Sprague-Dawley Rats. *Nutr. Cancer* **2003**, *47*, 141–147, doi:10.1207/s15327914nc4702_6.
63. Vissac-Sabatier, C.; Coxam, V.; Déchelotte, P.; Picherit, C.; Horcajada, M.-N.; Davicco, M.-J.; Lebecque, P.; Bignon, Y.-J.; Bernard-Gallon, D. Phytoestrogen-rich diets modulate expression of Brca1 and Brca2 tumor suppressor genes in mammary glands of female Wistar rats. *Cancer Res.* **2003**, *63*, 6607–6612.
64. Dave, B.; Eason, R.R.; Till, S.R.; Geng, Y.; Velarde, M.C.; Badger, T.M.; Simmen, R.C.M. The soy isoflavone genistein promotes apoptosis in mammary epithelial cells by inducing the tumor suppressor PTEN. *Carcinogenesis* **2005**, *26*, 1793–1803, doi:10.1093/carcin/bgi131.
65. Constantinou, A.I.; White, B.E.P.; Tonetti, D.; Yang, Y.; Liang, W.; Li, W.; van Breemen, R.B. The soy isoflavone daidzein improves the capacity of tamoxifen to prevent mammary tumours. *Eur. J. Cancer* **2005**, *41*, 647–654, doi:10.1016/j.ejca.2004.12.005.
66. Simmen, R.C.M.; Eason, R.R.; Till, S.R.; Chatman, L.; Velarde, M.C.; Geng, Y.; Korourian, S.; Badger, T.M. Inhibition of NMU-induced mammary tumorigenesis by dietary soy. *Cancer Lett.* **2005**, *224*, 45–52, doi:10.1016/j.canlet.2004.11.009.
67. Mukhopadhyay, S.; Ballard, B.R.; Mukherjee, S.; Kabir, S.M.; Das, S.K. Beneficial effects of soy protein in the initiation and progression against dimethylbenz [a] anthracene-induced breast tumors in female rats. *Mol. Cell. Biochem.* **2006**, *290*, 169–176, doi:10.1007/s11010-006-9184-9.
68. Manjanatha, M.G.; Shelton, S.; Bishop, M.E.; Lyn-Cook, L.E.; Aidoo, A. Dietary effects of soy isoflavones daidzein and genistein on 7,12-dimethylbenz[a]anthracene-induced mammary mutagenesis and carcinogenesis in ovariectomized Big Blue(R) transgenic rats. *Carcinogenesis* **2006**, *27*, 2555–2564, doi:10.1093/carcin/bgl195.
69. Su, Y.; Eason, R.R.; Geng, Y.; Till, S.R.; Badger, T.M.; Simmen, R.C.M. In utero exposure to maternal diets containing soy protein isolate, but not genistein alone, protects young adult rat offspring from NMU-induced mammary tumorigenesis. *Carcinogenesis* **2007**, *28*, 1046–1051, doi:10.1093/carcin/bgl240.
70. Qin, L.-Q.; Xu, J.-Y.; Tezuka, H.; Wang, P.-Y.; Hoshi, K. Commercial Soy Milk Enhances the Development of 7,12-Dimethylbenz(a)anthracene-induced Mammary Tumors in Rats. *In Vivo* **2007**, 5.
71. Kramer, F.; Johnson, I.T.; Doleman, J.F.; Lund, E.K. A comparison of the effects of soya isoflavonoids and fish oil on cell proliferation, apoptosis and the expression of oestrogen receptors α and β in the mammary gland and colon of the rat. *Br. J. Nutr.* **2008**, *102*, 29–36, doi:10.1017/S0007114508137862.

72. Kang, X.; Jin, S.; Zhang, Q. Antitumor and Antiangiogenic Activity of Soy Phytoestrogen on 7,12-Dimethylbenz[α]anthracene-Induced Mammary Tumors Following Ovariectomy in Sprague-Dawley Rats. *J. Food Sci.* **2009**, *74*, H237–H242, doi:10.1111/j.1750-3841.2009.01278.x.
73. Park, K.; Choi, K.; Kim, H.; Kim, K.; Lee, M.H.; Lee, J.-H.; Kim Rim, J.C. Isoflavone-deprived soy peptide suppresses mammary tumorigenesis by inducing apoptosis. *Exp. Mol. Med.* **2009**, *41*, 371, doi:10.3858/emm.2009.41.6.042.
74. Sahin, K.; Tuzcu, M.; Sahin, N.; Akdemir, F.; Ozercan, I.; Bayraktar, S.; Kucuk, O. Inhibitory Effects of Combination of Lycopene and Genistein on 7,12- Dimethyl Benz(a)anthracene-Induced Breast Cancer in Rats. *Nutr. Cancer* **2011**, *63*, 1279–1286, doi:10.1080/01635581.2011.606955.
75. Hakkak, R.; Shaaf, S.; Jo, C.H.; Macleod, S.; Korourian, S. Effects of high-isoflavone soy diet vs. casein protein diet and obesity on DMBA-induced mammary tumor development. *Oncol. Lett.* **2011**, *2*, 29–36, doi:10.3892/ol.2010.202.
76. Ono, M.; Koga, T.; Ueo, H.; Nakano, S. Effects of Dietary Genistein on Hormone-Dependent Rat Mammary Carcinogenesis Induced by Ethyl Methanesulphonate. *Nutr. Cancer* **2012**, *64*, 1204–1210, doi:10.1080/01635581.2012.718035.
77. Kaga, C.; Takagi, A.; Kano, M.; Kado, S.; Kato, I.; Sakai, M.; Miyazaki, K.; Nanno, M.; Ishikawa, F.; Ohashi, Y.; et al. *Lactobacillus casei* Shirota enhances the preventive efficacy of soymilk in chemically induced breast cancer. *Cancer Sci.* **2013**, *104*, 1508–1514, doi:10.1111/cas.12268.
78. Hsieh, C.Y.; Santell, R.C.; Haslam, S.Z.; Helferich, W.G. Estrogenic effects of genistein on the growth of estrogen receptor-positive human breast cancer (MCF-7) cells in vitro and in vivo. *Cancer Res.* **1998**, *58*, 3833–3838.
79. Allred, C.D.; Ju, Y.H.; Allred, K.F.; Chang J.; Helferich, W.G. Dietary genistin stimulates growth of estrogen-dependent breast cancer tumors similar to that observed with genistein. *Carcinogenesis*. **2001**, 22, 1667-1673.
80. Allred, C.D.; Allred, K.F.; Ju, Y.H.; Virant, S.M.; Helferich, W.G. Soy diets containing varying amounts of genistein stimulate growth of estrogen-dependent (MCF-7) tumors in a dose-dependent manner. *Cancer Res.* **2001**, *61*, 5045–5050.
81. Day, J.K.; Besch-Williford, C.; McMann, T.R.; Hufford, M.G.; Lubahn, D.B.; MacDonald, R.S. Dietary Genistein Increased DMBA-Induced Mammary Adenocarcinoma in Wild-Type, but Not ERαKO, Mice. *Nutr. Cancer* **2001**, *39*, 226–232, doi:10.1207/S15327914nc392_11.
82. Ju, Y.H.; Allred, C.D.; Allred, K.F.; Karko, K.L.; Doerge, D.R.; Helferich, W.G. Physiological Concentrations of Dietary Genistein Dose-Dependently Stimulate Growth of Estrogen-Dependent Human Breast Cancer (MCF-7) Tumors Implanted in Athymic Nude Mice. *J. Nutr.* **2001**, *131*, 2957–2962, doi:10.1093/jn/131.11.2957.
83. Ju, Y.H.; Doerge, D.R.; Allred, K.F.; Allred, C.D.; Helferich, W.G. Dietary genistein negates the inhibitory effect of tamoxifen on growth of estrogen-dependent human breast cancer (MCF-7) cells implanted in athymic mice. *Cancer Res.* **2002**, *62*, 2474–2477.
84. Jin, Z.; MacDonald, R.S. Soy Isoflavones Increase Latency of Spontaneous Mammary Tumors in Mice. *J. Nutr.* **2002**, *132*, 3186–3190, doi:10.1093/jn/131.10.3186.
85. Yang, X.; Edgerton, S.M.; Kosanke, S.D.; Mason, T.L.; Alvarez, K.M.; Liu, N.; Chatterton, R.T.; Liu, B.; Wang, Q.; Kim, A.; et al. Hormonal and dietary modulation of mammary carcinogenesis in mouse mammary tumor virus-c-erbB-2 transgenic mice. *Cancer Res.* **2003**, *63*, 2425–2433.
86. Allred, C.D. Soy processing influences growth of estrogen-dependent breast cancer tumors. *Carcinogenesis* **2004**, *25*, 1649–1657, doi:10.1093/carcin/bgh178.
87. Zhou, J.-R.; Yu, L.; Mai, Z.; Blackburn, G.L. Combined inhibition of estrogen-dependent human breast carcinoma by soy and tea bioactive components in mice. *Int. J. Cancer* **2004**, *108*, 8–14, doi:10.1002/ijc.11549.
88. Liu, B.; Edgerton, S.; Yang, X.; Kim, A.; Ordonez-Ercan, D.; Mason, T.; Alvarez, K.; McKimmey, C.; Liu, N.; Thor, A. Low-Dose Dietary Phytoestrogen Abrogates Tamoxifen-Associated Mammary Tumor Prevention. *Cancer Res* **2005**, 9.
89. Thomsen, A.R.; Mortensen, A.; Breinholt, V.M.; Lindecrona, R.H.; Penalvo, J.L.; Sorensen, I.K. Influence of Prevastein®, an Isoflavone-Rich Soy Product, on Mammary Gland Development and Tumorigenesis in Tg.NK (MMTV/c-neu) Mice. *Nutr. Cancer* **2005**, *52*, 176–188, doi:10.1207/s15327914nc5202_8.
90. Ju, Y.H.; Allred, K.F.; Allred, C.D.; Helferich, W.G. Genistein stimulates growth of human breast cancer cells in a novel, postmenopausal animal model, with low plasma estradiol concentrations. *Carcinogenesis* **2006**, *27*, 1292–1299, doi:10.1093/carcin/bgi370.

91. Ju, Y.H.; Fultz, J.; Allred, K.F.; Doerge, D.R.; Helferich, W.G. Effects of dietary daidzein and its metabolite, equol, at physiological concentrations on the growth of estrogen-dependent human breast cancer (MCF-7) tumors implanted in ovariectomized athymic mice. *Carcinogenesis* **2006**, *27*, 856–863, doi:10.1093/carcin/bgi320.
92. Mai, Z.; Blackburn, G.L.; Zhou, J.-R. Soy phytochemicals synergistically enhance the preventive effect of tamoxifen on the growth of estrogen-dependent human breast carcinoma in mice. *Carcinogenesis* **2007**, *28*, 1217–1223, doi:10.1093/carcin/bgm004.
93. Luijten, M.; Verhoef, A.; Dormans, J.; Beems, R.; Cremers, H.; Nagelkerke, N.; Adlercreutz, H.; Penalvo, J.; Piersma, A. Modulation of mammary tumor development in Tg.NK (MMTV/c-neu) mice by dietary fatty acids and life stage-specific exposure to phytoestrogens. *Reprod. Toxicol.* **2007**, *23*, 407–413, doi:10.1016/j.reprotox.2006.12.001.
94. Ju, Y.H.; Doerge, D.R.; Woodling, K.A.; Hartman, J.A.; Kwak, J.; Helferich, W.G. Dietary genistein negates the inhibitory effect of letrozole on the growth of aromatase-expressing estrogen-dependent human breast cancer cells (MCF-7Ca) in vivo. *Carcinogenesis* **2008**, *29*, 2162–2168, doi:10.1093/carcin/bgn161.
95. de Assis, S.; Warri, A.; Benitez, C.; Helferich, W.; Hilakivi-Clarke, L. Protective Effects of Prepubertal Genistein Exposure on Mammary Tumorigenesis Are Dependent on BRCA1 Expression. *Cancer Prev. Res. (Phila. Pa.)* **2011**, *4*, 1436–1448, doi:10.1158/1940-6207.CAPR-10-0346.
96. Zhang, G.; Han, D.; Liu, G.; Gao, S.; Cai, X.; Duan, R.; Feng, X. Effects of Soy Isoflavone and Endogenous Oestrogen on Breast Cancer in MMTV-erbB2 Transgenic Mice. *J. Int. Med. Res.* **2012**, *40*, 2073–2082, doi:10.1177/030006051204000604.
97. Davis, V.L.; Shaikh, F.; Gallagher, K.M.; Villegas, M.; Rea, S.L.; Cline, J.M.; Hughes, C.L. Inhibition of Neu-Induced Mammary Carcinogenesis in Transgenic Mice Expressing ERΔ3, a Dominant Negative Estrogen Receptor α Variant. *Horm. Cancer* **2012**, *3*, 227–239, doi:10.1007/s12672-012-0122-x.
98. Li, Y.; Meeran, S.M.; Patel, S.N.; Chen, H.; Hardy, T.M.; Tollefsbol, T.O. Epigenetic reactivation of estrogen receptor-α (ERα) by genistein enhances hormonal therapy sensitivity in ERα-negative breast cancer. *Mol. Cancer* **2013**, *12*, 9, doi:10.1186/1476-4598-12-9.
99. Rahal, O.M.; Machado, H.L.; Montales, M.T.E.; Pabona, J.M.P.; Heard, M.E.; Nagarajan, S.; Simmen, R.C.M. Dietary suppression of the mammary CD29hiCD24+ epithelial subpopulation and its cytokine/chemokine transcriptional signatures modifies mammary tumor risk in MMTV-Wnt1 transgenic mice. *Stem Cell Res.* **2013**, *11*, 1149–1162, doi:10.1016/j.scr.2013.08.006.
100. Watson, K.L.; Stalker, L.; Jones, R.A.; Moorehead, R.A. High levels of dietary soy decrease mammary tumor latency and increase incidence in MTB-IGFIR transgenic mice. *BMC Cancer* **2015**, *15*, 37, doi:10.1186/s12885-015-1037-z.
101. Paul, B.; Royston, K.J.; Li, Y.; Stoll, M.L.; Skibola, C.F.; Wilson, L.S.; Barnes, S.; Morrow, C.D.; Tollefsbol, T.O. Impact of genistein on the gut microbiome of humanized mice and its role in breast tumor inhibition. *PLOS ONE* **2017**, *12*, e0189756, doi:10.1371/journal.pone.0189756.
102. Paul, B.; Li, Y.; Tollefsbol, T. The Effects of Combinatorial Genistein and Sulforaphane in Breast Tumor Inhibition: Role in Epigenetic Regulation. *Int. J. Mol. Sci.* **2018**, *19*, 1754, doi:10.3390/ijms19061754.
103. Wang, B.; Xu, H.; Hu, X.; Ma, W.; Zhang, J.; Li, Y.; Yu, M.; Zhang, Y.; Li, X.; Ye, X. Synergetic inhibition of daidzein and regular exercise on breast cancer in bearing-4T1 mice by regulating NK cells and apoptosis pathway. *Life Sci.* **2020**, *245*, 117387, doi:10.1016/j.lfs.2020.117387.
104. Hirose, M.; Hoshiya, T.; Akagi, K.; Futakuchi, M.; Ito, N. Inhibition of mammary gland carcinogenesis by green tea catechins and other naturally occurring antioxidants in female Sprague-Dawley rats pretreated with 7,12-dimethylbenz[alpha]anthracene. *Cancer Lett.* **1994**, *83*, 149–156, doi:10.1016/0304-3835(94)90312-3.
105. Hirose, M.; Akagi, K.; Hasegawa, R.; Yaono, M.; Satoh, T.; Hara, Y.; Wakabayashi, K.; Ito, N. Chemoprevention of 2-amino-1-methyl-6-phenylimidazo[4,5-b]-pyridine (PhIP)-induced mammary gland carcinogenesis by antioxidants in F344 female rats. *Carcinogenesis* **1995**, *16*, 217–221, doi:10.1093/carcin/16.2.217.
106. Tanaka, H.; Hirose, M.; Kawabe, M.; Sano, M.; Yasuko Takesada; Hagiwara, A.; Shirai, T. Post-initiation inhibitory effects of green tea catechins on 7,12-dimethylbenz[a]anthracene-induced mammary gland carcinogenesis in female Sprague–Dawley rats. *Cancer Lett.* **1997**, *116*, 47–52, doi:10.1016/S0304-3835(97)04749-6.
107. Rogers, A. Black tea and mammary gland carcinogenesis by 7,12- dimethylbenz[a]anthracene in rats fed control or high fat diets. *Carcinogenesis* **1998**, *19*, 1269–1273, doi:10.1093/carcin/19.7.1269.
108. Kavanagh, K.T.; Hafer, L.J.; Kim, D.W.; Mann, K.K.; Sherr, D.H.; Rogers, A.E.; Sonenshein, G.E. Green tea extracts decrease carcinogen-induced mammary tumor burden in rats and rate of breast cancer cell proliferation in culture. *J. Cell. Biochem.* **2001**, *82*, 387–398, doi:10.1002/jcb.1164.

109. Singletary, K.W.; Meline, B. Effect of Grape Seed Proanthocyanidins on Colon Aberrant Crypts and Breast Tumors in a Rat Dual-Organ Tumor Model. *Nutr. Cancer* **2001**, *39*, 252–258, doi:10.1207/S15327914nc392_15.
110. Hirose, M.; Nishikawa, A.; Shibutani, M.; Imai, T.; Shirai, T. Chemoprevention of heterocyclic amine-induced mammary carcinogenesis in rats. *Environ. Mol. Mutagen.* **2002**, *39*, 271–278, doi:10.1002/em.10066.
111. Whitsett, T.; Carpenter, M.; Lamartiniere, C.A. Resveratrol, but not EGCG, in the diet suppresses DMBA-induced mammary cancer in rats. *J. Carcinog.* **2006**, *5*, 15, doi:10.1186/1477-3163-5-15.
112. Lubet, R.A.; Yang, C.S.; Lee, M.-J.; Hara, Y.; Kapetanovic, I.M.; Crowell, J.A.; Steele, V.E.; Juliana, M.M.; Grubbs, C.J. Preventive effects of Polyphenon E on urinary bladder and mammary cancers in rats and correlations with serum and urine levels of tea polyphenols. *Mol. Cancer Ther.* **2007**, *6*, 2022–2028, doi:10.1158/1535-7163.MCT-07-0058.
113. Baliga, M.S. Growth Inhibitory and Antimetastatic Effect of Green Tea Polyphenols on Metastasis-Specific Mouse Mammary Carcinoma 4T1 Cells In vitro and In vivo Systems. *Clin. Cancer Res.* **2005**, *11*, 1918–1927, doi:10.1158/1078-0432.CCR-04-1976.
114. Kaur, S.; Greaves, P.; Cooke, D.N.; Edwards, R.; Steward, W.P.; Gescher, A.J.; Marczylo, T.H. Breast Cancer Prevention by Green Tea Catechins and Black Tea Theaflavins in the C3(1) SV40 T,t Antigen Transgenic Mouse Model Is Accompanied by Increased Apoptosis and a Decrease in Oxidative DNA Adducts. *J. Agric. Food Chem.* **2007**, *55*, 3378–3385, doi:10.1021/jf0633342.
115. Thangapazham, R.L.; Singh, A.K.; Sharma, A.; Warren, J.; Gaddipati, J.P.; Maheshwari, R.K. Green tea polyphenols and its constituent epigallocatechin gallate inhibits proliferation of human breast cancer cells in vitro and in vivo. *Cancer Lett.* **2007**, *245*, 232–241, doi:10.1016/j.canlet.2006.01.027.
116. Gu, J.-W.; Makey, K.L.; Tucker, K.B.; Chinchar, E.; Mao, X.; Pei, I.; Thomas, E.Y.; Miele, L. EGCG, a major green tea catechin suppresses breast tumor angiogenesis and growth via inhibiting the activation of HIF-1α and NFκB, and VEGF expression. *Vasc. Cell* **2013**, *5*, 9, doi:10.1186/2045-824X-5-9.
117. Luo, K.-W.; Ko, C.-H.; Yue, G.G.-L.; Lee, J.K.-M.; Li, K.-K.; Lee, M.; Li, G.; Fung, K.-P.; Leung, P.-C.; Lau, C.B.-S. Green tea (Camellia sinensis) extract inhibits both the metastasis and osteolytic components of mammary cancer 4T1 lesions in mice. *J. Nutr. Biochem.* **2014**, *25*, 395–403, doi:10.1016/j.jnutbio.2013.11.013.
118. Xu, P.; Yan, F.; Zhao, Y.; Chen, X.; Sun, S.; Wang, Y.; Ying, L. Green Tea Polyphenol EGCG Attenuates MDSCs-mediated Immunosuppression through Canonical and Non-Canonical Pathways in a 4T1 Murine Breast Cancer Model. *Nutrients* **2020**, *12*, 1042, doi:10.3390/nu12041042.
119. Sartippour, M.R.; Pietras, R.; Marquez-Garban, D.C.; Chen, H.-W.; Heber, D.; Henning, S.M.; Sartippour, G.; Zhang, L.; Lu, M.; Weinberg, O.; et al. The combination of green tea and tamoxifen is effective against breast cancer. *Carcinogenesis* **2006**, *27*, 2424–2433, doi:10.1093/carcin/bgl066.
120. Kale, A.; Gawande, S.; Kotwal, S.; Netke, S.; Roomi, M.W.; Ivanov, V.; Niedzwiecki, A.; Rath, M. A combination of green tea extract, specific nutrient mixture and quercetin: An effective intervention treatment for the regression of N-methyl-N-nitrosourea (MNU)-induced mammary tumors in Wistar rats. *Oncol. Lett.* **2010**, *1*, 313–317, doi:10.3892/ol_00000056.
121. Sakata, M.; Ikeda, T.; Imoto, S.; Jinno, H. Prevention of Mammary Carcinogenesis in C3H/OuJ Mice by Green Tea and Tamoxifen. *Asian Pac. J. Cancer Prev.* **2011**, *12*, 5.
122. Bhat, K.P.; Lantvit, D.; Christov, K.; Mehta, R.G.; Moon, R.C.; Pezzuto, J.M. Estrogenic and antiestrogenic properties of resveratrol in mammary tumor models. *Cancer Res.* **2001**, *61*, 7456–7463.
123. Banerjee, S.; Bueso-Ramos, C.; Aggarwal, B.B. Suppression of 7,12-dimethylbenz(a)anthracene-induced mammary carcinogenesis in rats by resveratrol: role of nuclear factor-kappaB, cyclooxygenase 2, and matrix metalloprotease 9. *Cancer Res.* **2002**, *62*, 4945–4954.
124. Chatterjee, M.; Das, S.; Janarthan, M.; Ramachandran, H.K.; Chatterjee, M. Role of 5-lipoxygenase in resveratrol mediated suppression of 7,12-dimethylbenz(α)anthracene-induced mammary carcinogenesis in rats. *Eur. J. Pharmacol.* **2011**, *668*, 99–106, doi:10.1016/j.ejphar.2011.06.039.
125. Kisková, T.; Ekmekcioglu, C.; Garajová, M.; Orendáš, P.; Bojková, B.; Bobrov, N.; Jäger, W.; Kassayová, M.; Thalhammer, T. A combination of resveratrol and melatonin exerts chemopreventive effects in N-methyl-N-nitrosourea-induced rat mammary carcinogenesis: *Eur. J. Cancer Prev.* **2012**, *21*, 163–170, doi:10.1097/CEJ.0b013e32834c9c0f.
126. Qin, W.; Zhang, K.; Clarke, K.; Weiland, T.; Sauter, E.R. Methylation and miRNA Effects of Resveratrol on Mammary Tumors vs. Normal Tissue. *Nutr. Cancer* **2014**, *66*, 270–277, doi:10.1080/01635581.2014.868910.

127. Kisková, T.; Jendželovský, R.; Rentsen, E.; Maier-Salamon, A.; Kokošová, N.; Papčová, Z.; Mikeš, J.; Orendáš, P.; Bojková, B.; Kubatka, P.; et al. Resveratrol enhances the chemopreventive effect of celecoxib in chemically induced breast cancer in rats: *Eur. J. Cancer Prev.* **2014**, *23*, 506–513, doi:10.1097/CEJ.0000000000000083.
128. Papoutsis, A.J.; Selmin, O.I.; Borg, J.L.; Romagnolo, D.F. Gestational exposure to the AhR agonist 2,3,7,8-tetrachlorodibenzo- *p* -dioxin induces BRCA-1 promoter hypermethylation and reduces BRCA-1 expression in mammary tissue of rat offspring: Preventive effects of resveratrol: GESTATIONAL EXPOSURE TO THE AhR AGONIST 2,3,7,8-TETRACHLORODIBENZO-p-DIOXIN. *Mol. Carcinog.* **2015**, *54*, 261–269, doi:10.1002/mc.22095.
129. Jain, S.; Garg, T.; Kushwah, V.; Thanki, K.; Agrawal, A.K.; Dora, C.P. α-Tocopherol as functional excipient for resveratrol and coenzyme Q10-loaded SNEDDS for improved bioavailability and prophylaxis of breast cancer. *J. Drug Target.* **2017**, *25*, 554–565, doi:10.1080/1061186X.2017.1298603.
130. de Lima e Silva, T.C.; da Silveira, L.T.R.; Fragoso, M.F.; da Silva, F.R.M.; Martinez, M.F.; Zapaterini, J.R.; Diniz, O.H.G.; Scarano, W.R.; Barbisan, L.F. Maternal Resveratrol Treatment Reduces the Risk of Mammary Carcinogenesis in Female Offspring Prenatally Exposure to 2,3,7,8-Tetrachlorodibenzo-p-Dioxin. *Horm. Cancer* **2017**, *8*, 286–297, doi:10.1007/s12672-017-0304-7.
131. Kiskova, T.; Demeckova, V.; Jendzelovska, Z.; Kiktava, M.; Venglovska, K.; Bohmdorfer, M.; Jager, W.; Thalhammer, T. Nocturnal resveratrol administration inhibits chemically induced breast cancer formation in rats. *J. Physiol. Pharmacol. Off. J. Pol. Physiol. Soc.* **2017**, *68*, 867–875.
132. Zhou, X.; Zhao, Y.; Wang, J.; Wang, X.; Chen, C.; Yin, D.; Zhao, F.; Yin, J.; Guo, M.; Zhang, L.; et al. Resveratrol represses estrogen-induced mammary carcinogenesis through NRF2-UGT1A8-estrogen metabolic axis activation. *Biochem. Pharmacol.* **2018**, *155*, 252–263, doi:10.1016/j.bcp.2018.07.006.
133. Provinciali, M.; Re, F.; Donnini, A.; Orlando, F.; Bartozzi, B.; Di Stasio, G.; Smorlesi, A. Effect of resveratrol on the development of spontaneous mammary tumors in HER-2/neu transgenic mice. *Int. J. Cancer* **2005**, *115*, 36–45, doi:10.1002/ijc.20874.
134. Castillo-Pichardo, L.; Martínez-Montemayor, M.M.; Martínez, J.E.; Wall, K.M.; Cubano, L.A.; Dharmawardhane, S. Inhibition of mammary tumor growth and metastases to bone and liver by dietary grape polyphenols. *Clin. Exp. Metastasis* **2009**, *26*, 505–516, doi:10.1007/s10585-009-9250-2.
135. Castillo-Pichardo, L.; Dharmawardhane, S.F. Grape Polyphenols Inhibit Akt/Mammalian Target of Rapamycin Signaling and Potentiate the Effects of Gefitinib in Breast Cancer. *Nutr. Cancer* **2012**, *64*, 1058–1069, doi:10.1080/01635581.2012.716898.
136. Castillo-Pichardo, L.; Cubano, L.A.; Dharmawardhane, S. Dietary grape polyphenol resveratrol increases mammary tumor growth and metastasis in immunocompromised mice. *BMC Complement. Altern. Med.* **2013**, *13*, 6, doi:10.1186/1472-6882-13-6.
137. Andreani, C.; Bartolacci, C.; Wijnant, K.; Crinelli, R.; Bianchi, M.; Magnani, M.; Hysi, A.; Iezzi, M.; Amici, A.; Marchini, C. Resveratrol fuels HER2 and ERα-positive breast cancer behaving as proteasome inhibitor. *Aging* **2017**, *9*, 508–523, doi:10.18632/aging.101175.
138. García-Quiroz, J.; García-Becerra, R.; Santos-Cuevas, C.; Ramírez-Nava, G.J.; Morales-Guadarrama, G.; Cárdenas-Ochoa, N.; Segovia-Mendoza, M.; Prado-Garcia, H.; Ordaz-Rosado, D.; Avila, E.; et al. Synergistic Antitumorigenic Activity of Calcitriol with Curcumin or Resveratrol is Mediated by Angiogenesis Inhibition in Triple Negative Breast Cancer Xenografts. *Cancers* **2019**, *11*, 1739, doi:10.3390/cancers11111739.
139. Singletary, K.; MacDonald, C.; Iovinelli, M.; Fisher, C.; Wallig, M. Effect of the beta-diketones diferuloylmethane (curcumin) and dibenzoylmethane on rat mammary DNA adducts and tumors induced by 7,12-dimethylbenz[a]anthracene. *Carcinogenesis* **1998**, *19*, 1039–1043, doi:10.1093/carcin/19.6.1039.
140. Deshpande, S.S.; Ingle, A.D.; Maru, G.B. Chemopreventive efficacy of curcumin-free aqueous turmeric extract in 7,12-dimethylbenz[a]anthracene-induced rat mammary tumorigenesis. *Cancer Lett.* **1998**, *123*, 35–40, doi:10.1016/S0304-3835(97)00400-X.
141. Inano, H.; Onoda, M.; Inafuku, N.; Kubota, M.; Kamada, Y.; Osawa, T.; Kobayashi, H.; Wakabayashi, K. Chemoprevention by curcumin during the promotion stage of tumorigenesis of mammary gland in rats irradiated with gamma-rays. *Carcinogenesis* **1999**, *20*, 1011–1018, doi:10.1093/carcin/20.6.1011.
142. Inano, H. Potent preventive action of curcumin on radiation-induced initiation of mammary tumorigenesis in rats. *Carcinogenesis* **2000**, *21*, 1835–1841, doi:10.1093/carcin/21.10.1835.
143. Somasundaram, S.; Edmund, N.A.; Moore, D.T.; Small, G.W.; Shi, Y.Y.; Orlowski, R.Z. Dietary curcumin inhibits chemotherapy-induced apoptosis in models of human breast cancer. *Cancer Res.* **2002**, *62*, 3868–3875.

144. Aggarwal, B.B. Curcumin Suppresses the Paclitaxel-Induced Nuclear Factor- B Pathway in Breast Cancer Cells and Inhibits Lung Metastasis of Human Breast Cancer in Nude Mice. *Clin. Cancer Res.* **2005**, *11*, 7490–7498, doi:10.1158/1078-0432.CCR-05-1192.
145. Bachmeier, B.; Nerlich, A.; Iancu, C.; Cilli, M.; Schleicher, E.; Vené, R.; Dell'Eva, R.; Jochum, M.; Albini, A.; Pfeffer, U. The Chemopreventive Polyphenol Curcumin Prevents Hematogenous Breast Cancer Metastases in Immunodeficient Mice. *Cell. Physiol. Biochem.* **2007**, *19*, 137–152, doi:10.1159/000099202.
146. Ibrahim, A.; El-Meligy, A.; Fetaih, H.; Dessouki, A.; Stoica, G.; Barhoumi, R. Effect of Curcumin and Meriva on the Lung Metastasis of Murine Mammary Gland Adenocarcinoma. *In Vivo* **2010**, 8.
147. Bimonte, S.; Barbieri, A.; Palma, G.; Rea, D.; Luciano, A.; D'Aiuto, M.; Arra, C.; Izzo, F. Dissecting the Role of Curcumin in Tumour Growth and Angiogenesis in Mouse Model of Human Breast Cancer. *BioMed Res. Int.* **2015**, *2015*, 1–7, doi:10.1155/2015/878134.
148. Somers-Edgar, T.J.; Scandlyn, M.J.; Stuart, E.C.; Nedelec, M.J.L.; Valentine, S.P.; Rosengren, R.J. The combination of epigallocatechin gallate and curcumin suppresses ERα-breast cancer cell growth in vitro and in vivo. *Int. J. Cancer* **2008**, *122*, 1966–1971, doi:10.1002/ijc.23328.
149. Kang, H.J.; Lee, S.H.; Price, J.E.; Kim, L.S. Curcumin suppresses the paclitaxel-induced nuclear factor-kappaB in breast cancer cells and potentiates the growth inhibitory effect of paclitaxel in a breast cancer nude mice model. *Breast J.* **2009**, *15*, 223–229, doi:10.1111/j.1524-4741.2009.00709.x.
150. Sen, G.S.; Mohanty, S.; Hossain, D.M.S.; Bhattacharyya, S.; Banerjee, S.; Chakraborty, J.; Saha, S.; Ray, P.; Bhattacharjee, P.; Mandal, D.; et al. Curcumin Enhances the Efficacy of Chemotherapy by Tailoring p65NFκB-p300 Cross-talk in Favor of p53-p300 in Breast Cancer. *J. Biol. Chem.* **2011**, *286*, 42232–42247, doi:10.1074/jbc.M111.262295.
151. Royt, M.; Mukherjee, S.; Sarkar, R.; Biswas, J. Curcumin sensitizes chemotherapeutic drugs via modulation of PKC, telomerase, NF-kappaB and HDAC in breast cancer. *Ther. Deliv.* **2011**, *2*, 1275–1293, doi:10.4155/tde.11.97.
152. Siddiqui, R.A.; Harvey, K.A.; Walker, C.; Altenburg, J.; Xu, Z.; Terry, C.; Camarillo, I.; Jones-Hall, Y.; Mariash, C. Characterization of synergistic anti-cancer effects of docosahexaenoic acid and curcumin on DMBA-induced mammary tumorigenesis in mice. *BMC Cancer* **2013**, *13*, 418, doi:10.1186/1471-2407-13-418.
153. Choi, Y.E.; Park, E. Curcumin enhances poly(ADP-ribose) polymerase inhibitor sensitivity to chemotherapy in breast cancer cells. *J. Nutr. Biochem.* **2015**, *26*, 1442–1447, doi:10.1016/j.jnutbio.2015.07.015.
154. Mohapatra, P.; Satapathy, S.R.; Siddharth, S.; Das, D.; Nayak, A.; Kundu, C.N. Resveratrol and curcumin synergistically induces apoptosis in cigarette smoke condensate transformed breast epithelial cells through a p21Waf1/Cip1 mediated inhibition of Hh-Gli signaling. *Int. J. Biochem. Cell Biol.* **2015**, *66*, 75–84, doi:10.1016/j.biocel.2015.07.009.
155. Falah, R.R.; Talib, W.H.; Shbailat, S.J. Combination of metformin and curcumin targets breast cancer in mice by angiogenesis inhibition, immune system modulation and induction of p53 independent apoptosis. *Ther. Adv. Med. Oncol.* **2017**, *9*, 235–252, doi:10.1177/1758834016687482.
156. Moghtaderi, H.; Sepehri, H.; Attari, F. Combination of arabinogalactan and curcumin induces apoptosis in breast cancer cells in vitro and inhibits tumor growth via overexpression of p53 level in vivo. *Biomed. Pharmacother. Biomedecine Pharmacother.* **2017**, *88*, 582–594, doi:10.1016/j.biopha.2017.01.072.
157. Masuelli, L.; Granato, M.; Benvenuto, M.; Mattera, R.; Bernardini, R.; Mattei, M.; d'Amati, G.; D'Orazi, G.; Faggioni, A.; Bei, R.; et al. Chloroquine supplementation increases the cytotoxic effect of curcumin against Her2/neu overexpressing breast cancer cells in vitro and in vivo in nude mice while counteracts it in immune competent mice. *Oncoimmunology* **2017**, *6*, e1356151, doi:10.1080/2162402X.2017.1356151.
158. Liu, G.; Khanna, V.; Kirtane, A.; Grill, A.; Panyam, J. Chemopreventive efficacy of oral curcumin: a prodrug hypothesis. *FASEB J.* **2019**, *33*, 9453–9465, doi:10.1096/fj.201900166R.
159. Attia, Y.M.; El-Kersh, D.M.; Ammar, R.A.; Adel, A.; Khalil, A.; Walid, H.; Eskander, K.; Hamdy, M.; Reda, N.; Mohsen, N.E.; et al. Inhibition of aldehyde dehydrogenase-1 and p-glycoprotein-mediated multidrug resistance by curcumin and vitamin D3 increases sensitivity to paclitaxel in breast cancer. *Chem. Biol. Interact.* **2020**, *315*, 108865, doi:10.1016/j.cbi.2019.108865.
160. Serraino, M.; Thompson, L.U. The effect of flaxseed supplementation on the initiation and promotional stages of mammary tumorigenesis. *Nutr. Cancer* **1992**, *17*, 153–159, doi:10.1080/01635589209514182.
161. Serraino, M.R.; Thompson, L.U.; Cunnane, S.C. Effect of low level flaxseed suplementation on the fatty acid composition of mammary glands and tumors in rats. *Nutr. Res.* **1992**, *12*, 767–772, doi:10.1016/S0271-5317(05)80573-1.

162. Thompson, L.U.; Rickard, S.E.; Orcheson, L.J.; Seidl, M.M. SHORT COMMUNICATION: Flaxseed and its lignan and oil components reduce mammary tumor growth at a late stage of carcinogenesis. *Carcinogenesis* **1996**, *17*, 1373–1376, doi:10.1093/carcin/17.6.1373.
163. Rickard, S.E.; Yuan, Y.V.; Chen, J.; Thompson, L.U. Dose Effects of Flaxseed and Its Lignan on N-Methyl-N-Nitrosourea-Induced Mammary Tumorigenesis in Rats. *Nutr. Cancer* **1999**, *35*, 50–57, doi:10.1207/S1532791450-57.
164. Rickard, S.E.; Yuan, Y.V.; Thompson, L.U. Plasma insulin-like growth factor I levels in rats are reduced by dietary supplementation of ¯axseed or its lignan secoisolariciresinol diglycoside. *Cancer Lett.* **2000**, 9.
165. Chen, J.; Tan, K.P.; Ward, W.E.; Thompson, L.U. Exposure to Flaxseed or Its Purified Lignan during Suckling Inhibits Chemically Induced Rat Mammary Tumorigenesis. *Exp. Biol. Med.* **2003**, *228*, 951–958, doi:10.1177/153537020322800811.
166. Yu, B.; Khan, G.; Foxworth, A.; Huang, K.; Hilakivi-Clarke, L. Maternal dietary exposure to fiber during pregnancy and mammary tumorigenesis among rat offspring. *Int. J. Cancer* **2006**, *119*, 2279–2286, doi:10.1002/ijc.22201.
167. Khan, G.; Penttinen, P.; Cabanes, A.; Foxworth, A.; Chezek, A.; Mastropole, K.; Yu, B.; Smeds, A.; Halttunen, T.; Good, C. Maternal flaxseed diet during pregnancy or lactation increases female rat offspring's susceptibility to carcinogen-induced mammary tumorigenesis. *Reprod. Toxicol.* **2007**, *23*, 397–406, doi:10.1016/j.reprotox.2007.02.002.
168. Mabrok, H.B.; Klopfleisch, R.; Ghanem, K.Z.; Clavel, T.; Blaut, M.; Loh, G. Lignan transformation by gut bacteria lowers tumor burden in a gnotobiotic rat model of breast cancer. *Carcinogenesis* **2012**, *33*, 203–208, doi:10.1093/carcin/bgr256.
169. Delman, D.M.; Fabian, C.J.; Kimler, B.F.; Yeh, H.; Petroff, B.K. Effects of Flaxseed Lignan Secoisolariciresinol Diglucosideon Preneoplastic Biomarkers of Cancer Progression in a Model of Simultaneous Breast and Ovarian Cancer Development. *Nutr. Cancer* **2015**, *67*, 857–864, doi:10.1080/01635581.2015.1042549.
170. Rao, G.N.; Ney, E.; Herbert, R.A. Effect of Melatonin and Linolenic Acid on Mammary Cancer in Transgenic Mice with c-neu Breast Cancer Oncogene. *Breast Cancer Res. Treat.* **2000**, *64*, 287–296, doi:10.1023/A:1026552405042.
171. Chen, J.; Stavro, P.M.; Thompson, L.U. Dietary Flaxseed Inhibits Human Breast Cancer Growth and Metastasis and Downregulates Expression of Insulin-Like Growth Factor and Epidermal Growth Factor Receptor. *Nutr. Cancer* **2002**, *43*, 187–192, doi:10.1207/S15327914NC432_9.
172. Dabrosin, C.; Chen, J.; Wang, L.; Thompson, L.U. Flaxseed inhibits metastasis and decreases extracellular vascular endothelial growth factor in human breast cancer xenografts. *Cancer Lett.* **2002**, *185*, 31–37, doi:10.1016/S0304-3835(02)00239-2.
173. Chen, J. Dietary Flaxseed Enhances the Inhibitory Effect of Tamoxifen on the Growth of Estrogen-Dependent Human Breast Cancer (MCF-7) in Nude Mice. *Clin. Cancer Res.* **2004**, *10*, 7703–7711, doi:10.1158/1078-0432.CCR-04-1130.
174. Wang, L.; Chen, J.; Thompson, L.U. The inhibitory effect of flaxseed on the growth and metastasisof estrogen receptor negative human breast cancer xenograftsis attributed to both its lignan and oil components. *Int. J. Cancer* **2005**, *116*, 793–798, doi:10.1002/ijc.21067.
175. Chen, J.; Wang, L.; Thompson, L.U. Flaxseed and its components reduce metastasis after surgical excision of solid human breast tumor in nude mice. *Cancer Lett.* **2006**, *234*, 168–175, doi:10.1016/j.canlet.2005.03.056.
176. Saarinen, N.M.; Power, K.; Chen, J.; Thompson, L.U. Flaxseed attenuates the tumor growth stimulating effect of soy protein in ovariectomized athymic mice with MCF-7 human breast cancer xenografts. *Int. J. Cancer* **2006**, *119*, 925–931, doi:10.1002/ijc.21898.
177. Bergman Jungestrom, M.; Thompson, L.U.; Dabrosin, C. Flaxseed and Its Lignans Inhibit Estradiol-Induced Growth, Angiogenesis, and Secretion of Vascular Endothelial Growth Factor in Human Breast Cancer Xenografts In vivo. *Clin. Cancer Res.* **2007**, *13*, 1061–1067, doi:10.1158/1078-0432.CCR-06-1651.
178. Chen, J.; Power, K.A.; Mann, J.; Cheng, A.; Thompson, L.U. Dietary Flaxseed Interaction With Tamoxifen Induced Tumor Regression in Athymic Mice With MCF-7 Xenografts by Downregulating the Expression of Estrogen Related Gene Products and Signal Transduction Pathways. *Nutr. Cancer* **2007**, *58*, 162–170, doi:10.1080/01635580701328271.
179. Chen, J.; Power, K.A.; Mann, J.; Cheng, A.; Thompson, L.U. Flaxseed Alone or in Combination with Tamoxifen Inhibits MCF-7 Breast Tumor Growth in Ovariectomized Athymic Mice with High Circulating Levels of Estrogen. *Exp. Biol. Med.* **2007**, *232*, 1071–1080, doi:10.3181/0702-RM-36.
180. Power, K.A.; Chen, J.M.; Saarinen, N.M.; Thompson, L.U. Changes in biomarkers of estrogen receptor and growth factor signaling pathways in MCF-7 tumors after short- and long-term treatment with soy and flaxseed. *J. Steroid Biochem. Mol. Biol.* **2008**, *112*, 13–19, doi:10.1016/j.jsbmb.2008.07.003.

181. Zhang, L.; Wang, X.; Sun, D.; Liu, X.; Hu, X.; Kong, F. Regulation of zinc transporters by dietary flaxseed lignan in human breast cancer xenografts. *Mol. Biol. Rep.* **2008**, *35*, 595–600, doi:10.1007/s11033-007-9129-8.
182. Chen, J.; Saggar, J.K.; Corey, P.; Thompson, L.U. Flaxseed and Pure Secoisolariciresinol Diglucoside, but Not Flaxseed Hull, Reduce Human Breast Tumor Growth (MCF-7) in Athymic Mice. *J. Nutr.* **2009**, *139*, 2061–2066, doi:10.3945/jn.109.112508.
183. Zhou, Y.; Liu, Y.E.; Cao, J.; Zeng, G.; Shen, C.; Li, Y.; Zhou, M.; Chen, Y.; Pu, W.; Potters, L.; et al. Vitexins, Nature-Derived Lignan Compounds, Induce Apoptosis and Suppress Tumor Growth. *Clin. Cancer Res.* **2009**, *15*, 5161–5169, doi:10.1158/1078-0432.CCR-09-0661.
184. Mason, J.K.; Chen, J.; Thompson, L.U. Flaxseed oil–trastuzumab interaction in breast cancer. *Food Chem. Toxicol.* **2010**, *48*, 2223–2226, doi:10.1016/j.fct.2010.05.052.
185. Saggar, J.K.; Chen, J.; Corey, P.; Thompson, L.U. Dietary flaxseed lignan or oil combined with tamoxifen treatment affects MCF-7 tumor growth through estrogen receptor- and growth factor-signaling pathways. *Mol. Nutr. Food Res.* **2010**, *54*, 415–425, doi:10.1002/mnfr.200900068.
186. Saggar, J.K.; Chen, J.; Corey, P.; Thompson, L.U. The Effect of Secoisolariciresinol Diglucoside and Flaxseed Oil, Alone and in Combination, on MCF-7 Tumor Growth and Signaling Pathways. *Nutr. Cancer* **2010**, *62*, 533–542, doi:10.1080/01635580903532440.
187. Truan, J.S.; Chen, J.-M.; Thompson, L.U. Flaxseed oil reduces the growth of human breast tumors (MCF-7) at high levels of circulating estrogen. *Mol. Nutr. Food Res.* **2010**, *54*, 1414–1421, doi:10.1002/mnfr.200900521.
188. Birkved, F.K.; Mortensen, A.; Peñalvo, J.L.; Lindecrona, R.H.; Sørensen, I.K. Investigation into the cancer protective effect of flaxseed in Tg.NK (MMTV/c-neu) mice, a murine mammary tumor model. *Genes Nutr.* **2011**, *6*, 403–411, doi:10.1007/s12263-011-0214-1.
189. Chen, J.; Saggar, J.K.; Corey, P.; Thompson, L.U. Flaxseed cotyledon fraction reduces tumour growth and sensitises tamoxifen treatment of human breast cancer xenograft (MCF-7) in athymic mice. *Br. J. Nutr.* **2011**, *105*, 339–347, doi:10.1017/S0007114510003557.
190. Lindahl, G.; Saarinen, N.; Abrahamsson, A.; Dabrosin, C. Tamoxifen, Flaxseed, and the Lignan Enterolactone Increase Stroma- and Cancer Cell-Derived IL-1Ra and Decrease Tumor Angiogenesis in Estrogen-Dependent Breast Cancer. *Cancer Res.* **2011**, *71*, 51–60, doi:10.1158/0008-5472.CAN-10-2289.
191. Truan, J.S.; Chen, J.-M.; Thompson, L.U. Comparative Effects of Sesame Seed Lignan and Flaxseed Lignan in Reducing the Growth of Human Breast Tumors (MCF-7) at High Levels of Circulating Estrogen in Athymic Mice. *Nutr. Cancer* **2012**, *64*, 65–71, doi:10.1080/01635581.2012.630165.
192. Mason, J.K.; Fu, M.-H.; Chen, J.; Yu, Z.; Thompson, L.U. Dietary Flaxseed–Trastuzumab Interactive Effects on the Growth of HER2-Overexpressing Human Breast Tumors (BT-474). *Nutr. Cancer* **2013**, *65*, 451–459, doi:10.1080/01635581.2013.756921.
193. Mason, J.K.; Fu, M.; Chen, J.; Thompson, L.U. Flaxseed oil enhances the effectiveness of trastuzumab in reducing the growth of HER2-overexpressing human breast tumors (BT-474). *J. Nutr. Biochem.* **2015**, *26*, 16–23, doi:10.1016/j.jnutbio.2014.08.001.
194. Ezzat, S.M.; Shouman, S.A.; Elkhoely, A.; Attia, Y.M.; Elsesy, M.S.; El Senousy, A.S.; Choucry, M.A.; El Gayed, S.H.; El Sayed, A.A.; Sattar, E.A.; et al. Anticancer potentiality of lignan rich fraction of six Flaxseed cultivars. *Sci. Rep.* **2018**, *8*, 544, doi:10.1038/s41598-017-18944-0.
195. Li, J.; Li, K.; Gao, J.; Guo, X.; Lu, M.; Li, Z.; Li, D. Maternal exposure to an n-3 polyunsaturated fatty acid diet decreases mammary cancer risk of female offspring in adulthood. *Food Funct.* **2018**, *9*, 5768–5777, doi:10.1039/C8FO01006D.
196. Bowers, L.W.; Lineberger, C.G.; Ford, N.A.; Rossi, E.L.; Punjala, A.; Camp, K.K.; Kimler, B.K.; Fabian, C.J.; Hursting, S.D. The flaxseed lignan secoisolariciresinol diglucoside decreases local inflammation, suppresses NFκB signaling, and inhibits mammary tumor growth. *Breast Cancer Res. Treat.* **2019**, *173*, 545–557, doi:10.1007/s10549-018-5021-6.
197. Malewicz, B. Enhancement of mammary carcinogenesis in two rodent models by silymarin dietary supplements. *Carcinogenesis* **2006**, *27*, 1739–1747, doi:10.1093/carcin/bgl032.
198. Provinciali, M.; Papalini, F.; Orlando, F.; Pierpaoli, S.; Donnini, A.; Morazzoni, P.; Riva, A.; Smorlesi, A. Effect of the Silybin-Phosphatidylcholine Complex (IdB 1016) on the Development of Mammary Tumors in HER-2/neu Transgenic Mice. *Cancer Res.* **2007**, *67*, 2022–2029, doi:10.1158/0008-5472.CAN-06-2601.

199. Verschoyle, R.D.; Brown, K.; Steward, W.P.; Gescher, A.J. Consumption of silibinin, a flavonolignan from milk thistle, and mammary cancer development in the C3(1) SV40 T,t antigen transgenic multiple mammary adenocarcinoma (TAg) mouse. *Cancer Chemother. Pharmacol.* **2008**, *62*, 369–372, doi:10.1007/s00280-007-0611-8.
200. Forghani, P.; Khorramizadeh, M.R.; Waller, E.K. Silibinin inhibits accumulation of myeloid-derived suppressor cells and tumor growth of murine breast cancer. *Cancer Med.* **2014**, *3*, 215–224, doi:10.1002/cam4.186.
201. Kil, W.H.; Kim, S.M.; Lee, J.E.; Park, K.S.; Nam, S.J. Anticancer effect of silibinin on the xenograft model using MDA-MB-468 breast cancer cells. *Ann. Surg. Treat. Res.* **2014**, *87*, 167, doi:10.4174/astr.2014.87.4.167.
202. Ezzat, A.; Abdelhamid, A.O.; El Awady, M.K.; Abd El Azeem, A.S.; Mohammed, D.M. The biochemical effects of nano tamoxifen and some bioactive components in experimental breast cancer. *Biomed. Pharmacother.* **2017**, *95*, 571–576, doi:10.1016/j.biopha.2017.08.099.
203. Feldman, N.B.; Gromovykh, T.I.; Sedyakina, N.E.; Krasnyuk, I.I.; Lutsenko, S.V. Cytotoxic and Antitumor Activity of Liposomal Silibinin. *BioNanoScience* **2018**, *8*, 971–976, doi:10.1007/s12668-018-0556-x.
204. Ip, C.; Ganther, H.E. Combination of blocking agents and suppressing agents in cancer prevention. *Carcinogenesis* **1991**, *12*, 365–367, doi:10.1093/carcin/12.2.365.
205. Aiyer, H.S.; Gupta, R.C. Berries and Ellagic Acid Prevent Estrogen-Induced Mammary Tumorigenesis by Modulating Enzymes of Estrogen Metabolism. *Cancer Prev. Res. (Phila. Pa.)* **2010**, *3*, 727–737, doi:10.1158/1940-6207.CAPR-09-0260.
206. Munagala, R.; Aqil, F.; Vadhanam, M.V.; Gupta, R.C. MicroRNA 'signature' during estrogen-mediated mammary carcinogenesis and its reversal by ellagic acid intervention. *Cancer Lett.* **2013**, *339*, 175–184, doi:10.1016/j.canlet.2013.06.012.
207. Mandal, A.; Bishayee, A. Mechanism of Breast Cancer Preventive Action of Pomegranate: Disruption of Estrogen Receptor and Wnt/β-Catenin Signaling Pathways. *Molecules* **2015**, *20*, 22315–22328, doi:10.3390/molecules201219853.
208. Bishayee, A.; Mandal, A.; Bhattacharyya, P.; Bhatia, D. Pomegranate exerts chemoprevention of experimentally induced mammary tumorigenesis by suppression of cell proliferation and induction of apoptosis. *Nutr. Cancer* **2016**, *68*, 120–130, doi:10.1080/01635581.2016.1115094.
209. Chai, Y.; Lee, H.-J.; Shaik, A.A.; Nkhata, K.; Xing, C.; Zhang, J.; Jeong, S.-J.; Kim, S.-H.; Lu, J. Penta-O-galloyl-beta-D-glucose induces G1 arrest and DNA replicative S-phase arrest independently of cyclin-dependent kinase inhibitor 1A, cyclin-dependent kinase inhibitor 1B and P53 in human breast cancer cells and is orally active against triple negative xenograft growth. *Breast Cancer Res. BCR* **2010**, *12*, R67, doi:10.1186/bcr2634.
210. Lee, H.-J.; Seo, N.-J.; Jeong, S.-J.; Park, Y.; Jung, D.-B.; Koh, W.; Lee, H.-J.; Lee, E.-O.; Ahn, K.S.; Ahn, K.S.; et al. Oral administration of penta-O-galloyl- -D-glucose suppresses triple-negative breast cancer xenograft growth and metastasis in strong association with JAK1-STAT3 inhibition. *Carcinogenesis* **2011**, *32*, 804–811, doi:10.1093/carcin/bgr015.
211. Zhao, T.; Sun, Q.; del Rincon, S.V.; Lovato, A.; Marques, M.; Witcher, M. Gallotannin Imposes S Phase Arrest in Breast Cancer Cells and Suppresses the Growth of Triple-Negative Tumors In Vivo. *PLoS ONE* **2014**, *9*, e92853, doi:10.1371/journal.pone.0092853.
212. Yokdang, N.; Nordmeier, S.; Speirs, K.; R. Burkin, H.; L. O. Buxton, I. Blockade of extracellular NM23 or its endothelial target slows breast cancer growth and metastasis. *Integr. Cancer Sci. Ther.* **2015**, *2*, doi:10.15761/ICST.1000139.
213. Wang, N.; Wang, Q.; Tang, H.; Zhang, F.; Zheng, Y.; Wang, S.; Zhang, J.; Wang, Z.; Xie, X. Direct inhibition of ACTN4 by ellagic acid limits breast cancer metastasis via regulation of β-catenin stabilization in cancer stem cells. *J. Exp. Clin. Cancer Res.* **2017**, *36*, 172, doi:10.1186/s13046-017-0635-9.
